# Supplementary material for: In Silico Pleiotropy Analysis in KEGG Signaling Networks Using a Boolean Network Model
Source: Biomolecules. 2022 Aug 18;12(8):1139. doi: 10.3390/biom12081139 (PMC9406064; doi:10.3390/biom12081139)
Supplement: Supplementary file 1 [file biomolecules-12-01139-s001.zip › biomolecules-1849293-supplementary.pdf]

Supplementary Materials

In silico Pleiotropy Analysis in KEGG Signaling Networks Using a Boolean Network Model

Maulida Mazaya [1,†] and Yung-Keun Kwon [2,*]

[1] Research Center for Computing, National Research and Innovation Agency (BRIN), Cibinong Science Center, Jl. Raya Jakarta - Bogor KM 46, Cibinong 16911, West Java, Indonesia

[2] School of IT Convergence, University of Ulsan, 93 Daehak-ro, Nam-gu, Ulsan 44610, Korea

* Correspondence: kwonyk@ulsan.ac.kr

† This author is the main contributor to this work.

Supporting Text

Nested Canalyzing Function

The implementation of a *Nested Canalyzing Functions* (NCFs) model [1,2] was to describe Boolean function update rules. Given a Boolean network $G(V,A)$, the value of each variable v_i at time $t+1$ is determined by the values of k_i other variables $v_{i_1}, v_{i_2}, \ldots, v_{i_{k_i}}$ with a link to v_i at time t by the Boolean function f_i: $v_i(t+1) = f_i(v_{i_1}(t), v_{i_2}(t), \ldots, v_{i_{k_i}}(t))$. The rule f_i is called *canalyzing* on the input variable v_{i_m} if there exist Boolean values, I_m and O_m, such that

$$v_{i_m}(t) = I_m \quad \rightarrow \quad v_i(t+1) = O_m. \qquad (1)$$

Then, I_m and O_m are called the canalyzing and canalyzed values for the output variable v_i, respectively. Note that NCFs are a natural subset of canalyzing rules. It was inspired by a question of what happens in the non-canalyzing case: *When a rule is not canalyzed by the value of the first input variable, is it canalyzed by one of the remaining input variables?* The answer is this consecutive canalization test can be repeated for all inputs. Therefore, an NCF to update v_i can be represented as follows:

$$f_i\left(v_{i_1}(t), v_{i_2}(t), \ldots, v_{i_{k_i}}(t)\right)$$
$$= \begin{cases} O_1 & if\ v_{i_1}(t) = I_1 \\ O_2 & if v_{i_1}(t) \neq I_1 \text{ and } v_{i_2}(t) = I_2 \\ O_3 & if\ v_{i_1}(t) \neq I_1 \text{ and } v_{i_2}(t) \neq I_2 \text{ and } v_{i_3}(t) = I_3 \\ & \vdots \\ O_{k_i} & if\ v_{i_1}(t) \neq I_1 \cdots v_{i_{k_i-1}}(t) \neq I_{k_i-1} \text{ and } v_{i_{k_i}}(t) = I_{k_i} \\ O_{def} & otherwise \end{cases} \qquad (2)$$

where all I_m and O_m $(m = 1,2,\cdots,k_i)$ denote the canalyzing and canalyzed Boolean values, respectively, and O_{def} is generally set to $1 - O_{k_i}$. In this works, each NCF is randomized by specifying every I_m and O_m between 0 and 1 uniformly at random. In addition, we are independently and randomly specified $O_1, \cdots, O_{k_i}$ values with the probabilities

$$P(O_m = 1) = \frac{\exp(-2^{-m}\theta)}{1 + \exp(-2^{-m}\theta)} \qquad (3)$$

where θ is a constant. On the other hand, the value of I_m is deterministically specified by the value of O_m and the sign of the interaction from v_{i_m} to v_i $(m = 1, \cdots, k_i)$ that can be seen in Table 2.1.

Table 2.1 NCFs specification of I_m and O_m

O_m	*Sign of the interaction from* v_{i_m} *to* v_i	I_m
1	Positive ($v_{i_m} \rightarrow v_i$)	1
1	Negative ($v_{i_m} \dashv v_i$)	0
0	Positive ($v_{i_m} \rightarrow v_i$)	0
0	Negative ($v_{i_m} \dashv v_i$)	1

We note that NCFs have been shown to properly fit real biological experimental data [2,3] and many molecular interactions and analysis were successfully represented by NCFs [4-7]. For example, 133 out of 139 rules compiled from a dataset about a transcriptional regulatory network [5] or 39 out of 42 rules inferred from a signaling pathway dataset [4]. In addition, NCFs also support gene-gene pleiotropy analysis [8]. Those imply that NCFs-embedded random networks can describe the network dynamics considerably similarly to those real biological networks.

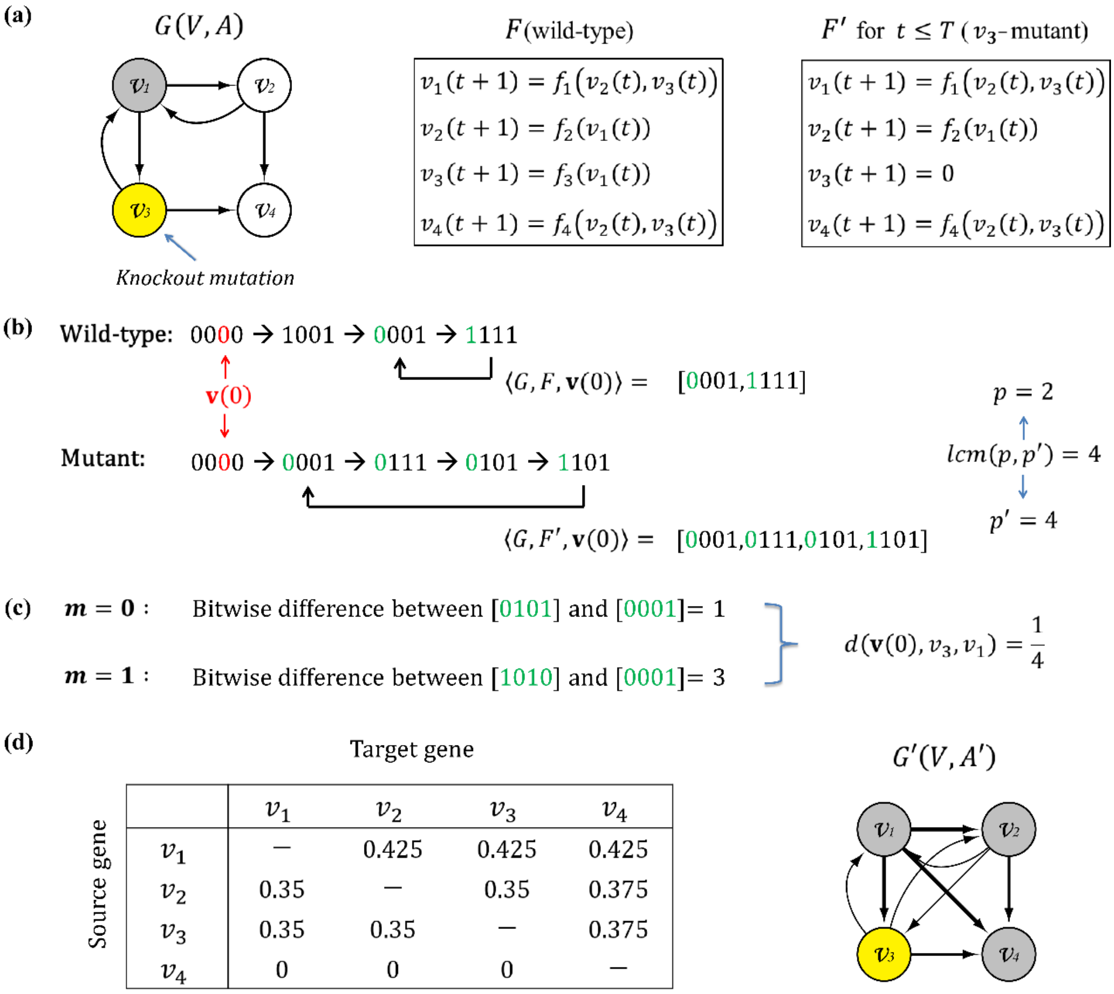

Figure S1. Dynamics influence computation example [9].

(a) An example GMI network. Given a network $G(V, A)$ with a set of update rules F, let v_3 a node subjected to the knockout mutation for $t \leq T$. The knockout mutation changes F into F' where the state value of v_3 is frozen to 0 for $t \leq T$. **(b)** Identification of wild-type and mutant attractors. Let $[0000] \in S$ be an initial state considered in this example. By examining two state trajectories along with F and F', respectively, obtain two corresponding attractors, $\langle G, F, \mathrm{v}(0)\rangle$ and $\langle G, F', \mathrm{v}(0)\rangle$ of which the *least common multiple* of the lengths is four. **(c)** Computation of a distance between wild-type and mutant attractors. Since the *greatest common divisor* of the lengths of two attractors is two, examine two different alignments of the state sequences of v_1 in those attractors. The number of different bits between $[0101]$ and $[0001]$ is 1 in case 1 ($m = 0$), whereas that between $[1010]$ and $[0001]$ is 3 in case 2 ($m = 1$). Accordingly, the minimum bitwise difference is 1, and hence $d(\mathrm{v}(0), v_3, v_1) = \frac{1}{4}$. Finally, compute $\mu(v_3, v_1)$ by averaging out $d(\mathrm{v}(0), v_3, v_1)$ over the set of initial states. **(d)** The resultant GDI network. The left matrix shows the dynamics influence value for every ordered pair of genes, and the right graph shows the resultant GDI network with nine positive dynamics influence relations.

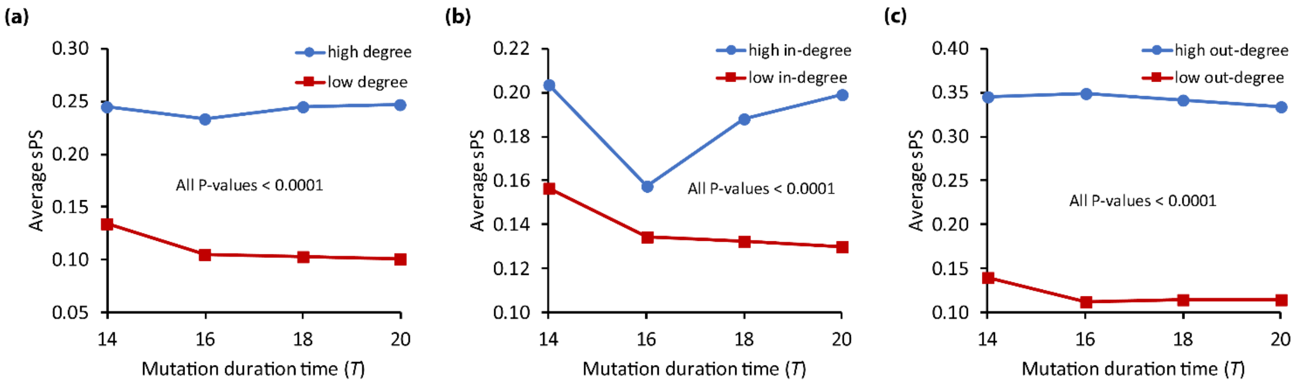

Figure S2. Relations of sPS and degree of nodes in KEGG network. All genes were classified into 'high/low degree/in-degree/out-degree' groups if any gene having high/low number of nodes' degree/in-degree/out-degree (blue/red lines). Y-axis means the average sPS for each group. Mutation duration time *T* was set from 14 to 20.

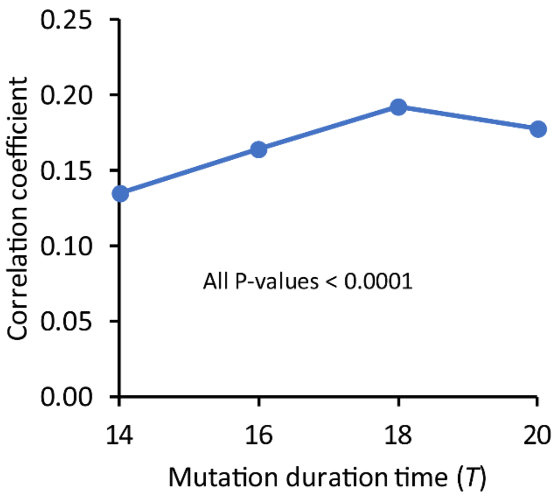

Figure S3. Relations of sPS values to feedback loops in KEGG network. All pairs of nodes were classified into 'FBL' and 'Non-FBL' groups such that any gene in the pair is involved with feedback loops or not. Y-axis values mean the correlation coefficient between $\boldsymbol{sPS}$ values and feedback loop. Mutation duration time was set to T=14-20.

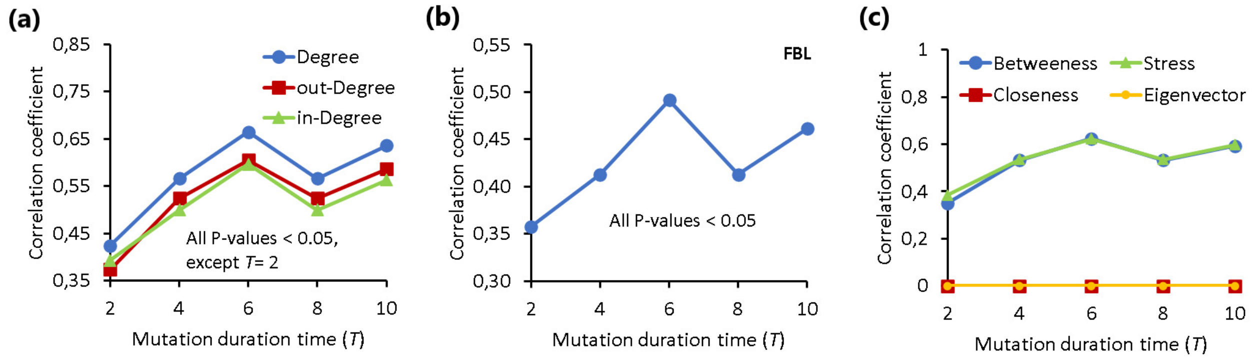

Figure S4. Relations of sPS value in random BA network.

A total of 250 BA random networks with $|V|$ = 50 and $|A|$ = 100 were generated. The time gap (T) was set to 2-10. (a) Relations to the degree of nodes. Y-axis values mean correlation coefficients between sPS and the number of nodes' degree, in-degree, and out-degree. (b) Relations to the feedback loops. All genes were classified into 'FBL' and 'No FBL' where gene is involving feedback loops or not, respectively. Y-axis values mean the correlation coefficient between $\boldsymbol{sPS}$ values and feedback loop. (c) Relations to the centrality measures including betweenness, stress, closeness, and eigenvector. Y-axis values mean the correlation coefficients between sPS and each centrality measures.

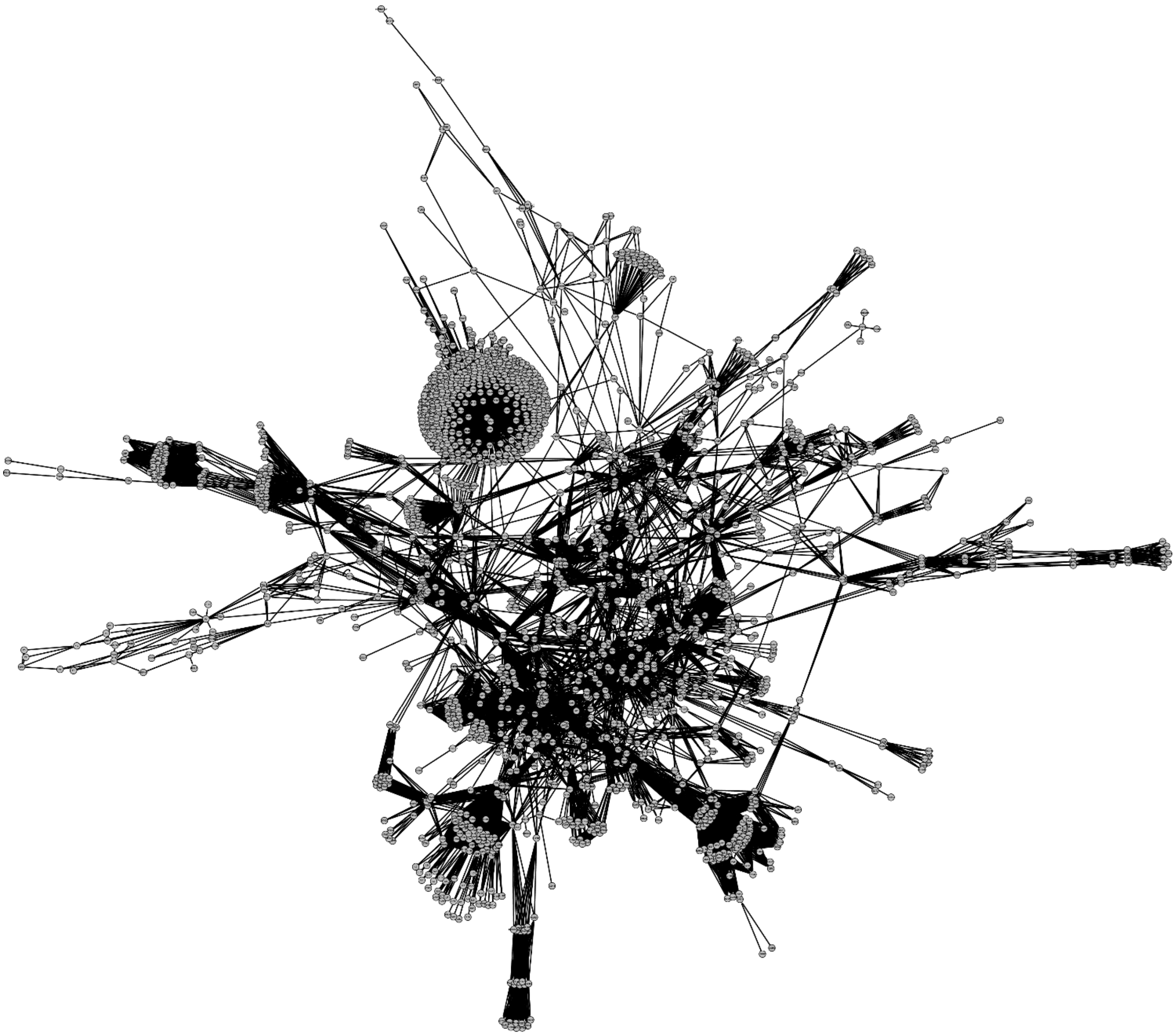

Figure S5. Original KEGG network with $|V|$=1659, $|A|$=7964.

Table S1. KEGG network dataset consists of 1659 nodes and 7964 interactions.

GeneID (source)	Interaction	GeneID (target)
9655	-1	7297
9655	-1	3718
9655	-1	3717
9655	-1	3716
9655	-1	9180
9655	-1	64109
9655	-1	58985
9655	-1	5618
9655	-1	53833
9655	-1	53832
9655	-1	50615
9655	-1	4352
9655	-1	3977
9655	-1	3953
9655	-1	3601
9655	-1	3598
9655	-1	3597
9655	-1	3595
9655	-1	3594
9655	-1	3590
9655	-1	3588
9655	-1	3587
9655	-1	3581
9655	-1	3575
9655	-1	3572
9655	-1	3570
9655	-1	3568
9655	-1	3566
9655	-1	3563
9655	-1	3561
9655	-1	3560
9655	-1	3559
9655	-1	3460
9655	-1	3459
9655	-1	3455
9655	-1	3454
9655	-1	2690
9655	-1	2057
9655	-1	163702
9655	-1	149233
9655	-1	1441
9655	-1	1439
9655	-1	1438
9655	-1	1271
9655	-1	116379
30837	-1	7297
30837	-1	3718
30837	-1	3717
30837	-1	3716
30837	-1	9180
30837	-1	64109

30837	-1	58985
30837	-1	5618
30837	-1	53833
30837	-1	53832
30837	-1	50615
30837	-1	4352
30837	-1	3977
30837	-1	3953
30837	-1	3601
30837	-1	3598
30837	-1	3597
30837	-1	3595
30837	-1	3594
30837	-1	3590
30837	-1	3588
30837	-1	3587
30837	-1	3581
30837	-1	3575
30837	-1	3572
30837	-1	3570
30837	-1	3568
30837	-1	3566
30837	-1	3563
30837	-1	3561
30837	-1	3560
30837	-1	3559
30837	-1	3460
30837	-1	3459
30837	-1	3455
30837	-1	3454
30837	-1	2690
30837	-1	2057
30837	-1	163702
30837	-1	149233
30837	-1	1441
30837	-1	1439
30837	-1	1438
30837	-1	1271
30837	-1	116379
5571	-1	32
5571	-1	31
5565	-1	32
5565	-1	31
5564	-1	32
5564	-1	31
53632	-1	32
53632	-1	31
51422	-1	32
51422	-1	31
157	-1	8590
157	-1	8392
157	-1	8390
157	-1	8388
157	-1	8387
157	-1	8386

157	-1	8385
157	-1	8383
157	-1	81797
157	-1	81697
157	-1	81696
157	-1	81472
157	-1	81470
157	-1	81469
157	-1	81448
157	-1	81442
157	-1	81399
157	-1	81392
157	-1	81328
157	-1	81327
157	-1	81318
157	-1	81309
157	-1	81300
157	-1	81285
157	-1	81282
157	-1	81168
157	-1	81127
157	-1	81099
157	-1	81061
157	-1	81050
157	-1	79544
157	-1	79541
157	-1	79501
157	-1	79473
157	-1	79345
157	-1	79339
157	-1	79324
157	-1	7932
157	-1	79317
157	-1	79310
157	-1	79295
157	-1	79290
157	-1	56656
157	-1	504189
157	-1	4995
157	-1	4994
157	-1	4993
157	-1	4992
157	-1	4991
157	-1	442361
157	-1	442194
157	-1	442191
157	-1	442186
157	-1	441933
157	-1	441911
157	-1	441670
157	-1	441669
157	-1	441639
157	-1	441608
157	-1	402317
157	-1	402135

157	-1	401994
157	-1	401993
157	-1	401992
157	-1	401667
157	-1	401666
157	-1	401665
157	-1	401427
157	-1	393046
157	-1	392392
157	-1	392391
157	-1	392390
157	-1	392376
157	-1	392309
157	-1	392138
157	-1	391211
157	-1	391196
157	-1	391195
157	-1	391194
157	-1	391192
157	-1	391191
157	-1	391190
157	-1	391189
157	-1	391114
157	-1	391112
157	-1	391109
157	-1	391107
157	-1	390892
157	-1	390883
157	-1	390882
157	-1	390649
157	-1	390648
157	-1	390538
157	-1	390445
157	-1	390442
157	-1	390439
157	-1	390437
157	-1	390436
157	-1	390433
157	-1	390431
157	-1	390429
157	-1	390327
157	-1	390326
157	-1	390323
157	-1	390321
157	-1	390275
157	-1	390265
157	-1	390264
157	-1	390261
157	-1	390260
157	-1	390201
157	-1	390199
157	-1	390197
157	-1	390195
157	-1	390191
157	-1	390181

157	-1	390174
157	-1	390168
157	-1	390167
157	-1	390162
157	-1	390157
157	-1	390155
157	-1	390154
157	-1	390152
157	-1	390151
157	-1	390144
157	-1	390142
157	-1	390113
157	-1	390093
157	-1	390084
157	-1	390083
157	-1	390081
157	-1	390079
157	-1	390078
157	-1	390077
157	-1	390075
157	-1	390072
157	-1	390067
157	-1	390066
157	-1	390064
157	-1	390063
157	-1	390061
157	-1	390059
157	-1	390058
157	-1	390054
157	-1	390038
157	-1	390037
157	-1	390036
157	-1	389090
157	-1	387748
157	-1	347468
157	-1	347169
157	-1	347168
157	-1	346528
157	-1	346525
157	-1	346517
157	-1	343563
157	-1	343406
157	-1	343173
157	-1	343172
157	-1	343171
157	-1	343169
157	-1	341799
157	-1	341568
157	-1	341418
157	-1	341416
157	-1	341276
157	-1	341152
157	-1	340980
157	-1	338755
157	-1	338751

157	-1	338675
157	-1	338674
157	-1	338662
157	-1	286365
157	-1	286362
157	-1	285659
157	-1	284532
157	-1	284521
157	-1	284433
157	-1	284383
157	-1	283694
157	-1	283365
157	-1	283297
157	-1	283189
157	-1	283162
157	-1	283160
157	-1	283159
157	-1	283111
157	-1	283093
157	-1	283092
157	-1	282775
157	-1	282770
157	-1	282763
157	-1	26740
157	-1	26737
157	-1	26735
157	-1	26716
157	-1	26707
157	-1	26696
157	-1	26692
157	-1	26689
157	-1	26686
157	-1	26683
157	-1	26682
157	-1	26664
157	-1	26659
157	-1	26658
157	-1	26539
157	-1	26538
157	-1	26534
157	-1	26533
157	-1	26532
157	-1	26531
157	-1	26529
157	-1	26496
157	-1	26494
157	-1	26493
157	-1	26492
157	-1	26476
157	-1	26339
157	-1	26338
157	-1	26333
157	-1	26248
157	-1	26246
157	-1	26245

157	-1	26219
157	-1	26212
157	-1	26211
157	-1	26189
157	-1	26188
157	-1	256892
157	-1	256148
157	-1	256144
157	-1	255725
157	-1	254973
157	-1	254879
157	-1	254786
157	-1	254783
157	-1	23538
157	-1	219986
157	-1	219983
157	-1	219982
157	-1	219981
157	-1	219968
157	-1	219965
157	-1	219960
157	-1	219959
157	-1	219958
157	-1	219957
157	-1	219956
157	-1	219954
157	-1	219952
157	-1	219875
157	-1	219874
157	-1	219873
157	-1	219870
157	-1	219869
157	-1	219865
157	-1	219858
157	-1	219493
157	-1	219487
157	-1	219484
157	-1	219482
157	-1	219479
157	-1	219477
157	-1	219473
157	-1	219469
157	-1	219464
157	-1	219453
157	-1	219447
157	-1	219438
157	-1	219437
157	-1	219436
157	-1	219432
157	-1	219431
157	-1	219429
157	-1	219428
157	-1	219417
157	-1	196335
157	-1	162998

157	-1	158131
157	-1	150681
157	-1	144125
157	-1	144124
157	-1	143503
157	-1	143502
157	-1	143496
157	-1	138883
157	-1	138882
157	-1	138881
157	-1	138805
157	-1	138804
157	-1	138803
157	-1	138802
157	-1	138799
157	-1	135948
157	-1	135946
157	-1	135941
157	-1	135924
157	-1	134083
157	-1	130075
157	-1	128372
157	-1	128371
157	-1	128368
157	-1	128367
157	-1	128360
157	-1	127623
157	▯ ą	127385
157	-1	127077
157	-1	127074
157	-1	127069
157	-1	127068
157	-1	127066
157	-1	127064
157	-1	127062
157	-1	127059
157	-1	126541
157	-1	126370
157	-1	125963
157	-1	125962
157	-1	125958
157	-1	124538
157	-1	122748
157	-1	122742
157	-1	122740
157	-1	121364
157	-1	121275
157	-1	121130
157	-1	120796
157	-1	120793
157	-1	120787
157	-1	120776
157	-1	120775
157	-1	120586
157	-1	120066

157	-1	120065
157	-1	119774
157	-1	119772
157	-1	119765
157	-1	119764
157	-1	119749
157	-1	119695
157	-1	119694
157	-1	119692
157	-1	119687
157	-1	119682
157	-1	119679
157	-1	119678
157	-1	10798
5598	-1	57369
5598	-1	2697
53944	-1	2737
53944	-1	2736
53944	-1	2735
1456	-1	2737
1456	-1	2736
1456	-1	2735
1455	-1	2737
1455	-1	2736
1455	-1	2735
1452	-1	2737
1452	-1	2736
1452	-1	2735
122011	-1	2737
122011	-1	2736
122011	-1	2735
51701	-1	83439
51701	-1	6934
51701	-1	6932
51701	-1	51176
6197	-1	7249
6196	-1	7249
6195	-1	7249
27330	-1	7249
5563	-1	32
5563	-1	31
5563	-1	2475
5562	-1	32
5562	-1	31
5562	-1	2475
5347	-1	9088
9088	-1	983
7465	-1	983
55844	-1	5590
55844	-1	5584
5522	-1	5590
5522	-1	5584
5521	-1	5590
5521	-1	5584
5520	-1	5590

5520	-1	5584
5495	-1	6885
5536	-1	4217
5494	-1	6416
5494	-1	5609
5494	-1	5608
5494	-1	650832
5494	-1	5606
80824	-1	6300
80824	-1	5603
80824	-1	5600
80824	-1	1432
80824	-1	5602
80824	-1	5601
80824	-1	5599
80824	-1	5595
80824	-1	5594
1852	-1	6300
1852	-1	5603
1852	-1	5600
1852	-1	1432
1852	-1	5602
1852	-1	5601
1852	-1	5599
1852	-1	5595
1852	-1	5594
1850	-1	6300
1850	-1	5603
1850	-1	5600
1850	-1	1432
1850	-1	5602
1850	-1	5601
1850	-1	5599
1850	-1	5595
1850	-1	5594
1849	-1	6300
1849	-1	5603
1849	-1	5600
1849	-1	1432
1849	-1	5602
1849	-1	5601
1849	-1	5599
1849	-1	5595
1849	-1	5594
1848	-1	6300
1848	-1	5603
1848	-1	5600
1848	-1	1432
1848	-1	5602
1848	-1	5601
1848	-1	5599
1848	-1	5595
1848	-1	5594
1847	-1	6300
1847	-1	5603

1847	-1	5600
1847	-1	1432
1847	-1	5602
1847	-1	5601
1847	-1	5599
1847	-1	5595
1847	-1	5594
1846	-1	6300
1846	-1	5603
1846	-1	5600
1846	-1	1432
1846	-1	5602
1846	-1	5601
1846	-1	5599
1846	-1	5595
1846	-1	5594
1845	-1	6300
1845	-1	5603
1845	-1	5600
1845	-1	1432
1845	-1	5602
1845	-1	5601
1845	-1	5599
1845	-1	5595
1845	-1	5594
1844	-1	6300
1844	-1	5603
1844	-1	5600
1844	-1	1432
1844	-1	5602
1844	-1	5601
1844	-1	5599
1844	-1	5595
1844	-1	5594
1843	-1	6300
1843	-1	5603
1843	-1	5600
1843	-1	1432
1843	-1	5602
1843	-1	5601
1843	-1	5599
1843	-1	5595
1843	-1	5594
11221	-1	6300
11221	-1	5603
11221	-1	5600
11221	-1	1432
11221	-1	5602
11221	-1	5601
11221	-1	5599
11221	-1	5595
11221	-1	5594
11072	-1	6300
11072	-1	5603
11072	-1	5600

11072	-1	1432
11072	-1	5602
11072	-1	5601
11072	-1	5599
11072	-1	5595
11072	-1	5594
84867	-1	6300
84867	-1	5603
84867	-1	5600
84867	-1	1432
84867	-1	5602
84867	-1	5601
84867	-1	5599
84867	-1	5595
84867	-1	5594
5801	-1	6300
5801	-1	5603
5801	-1	5600
5801	-1	1432
5801	-1	5602
5801	-1	5601
5801	-1	5599
5801	-1	5595
5801	-1	5594
5778	-1	6300
5778	-1	5603
5778	-1	5600
5778	-1	1432
5778	-1	5602
5778	-1	5601
5778	-1	5599
5778	-1	5595
5778	-1	5594
9586	-1	2033
9586	-1	1387
4149	-1	9063
4149	-1	8554
4149	-1	51588
4149	-1	10401
862	-1	1050
862	-1	6688
652671	-1	6688
652346	-1	6688
5914	-1	6688
5371	-1	6688
6647	-1	4747
6647	-1	79139
6647	-1	84134
6647	-1	10452
6647	-1	596
8835	-1	7297
8835	-1	3718
8835	-1	3717
8835	-1	3716
8835	-1	9180

8835	-1	64109
8835	-1	58985
8835	-1	5618
8835	-1	53833
8835	-1	53832
8835	-1	50615
8835	-1	4352
8835	-1	3977
8835	-1	3953
8835	-1	3601
8835	-1	3598
8835	-1	3597
8835	-1	3595
8835	-1	3594
8835	-1	3590
8835	-1	3588
8835	-1	3587
8835	-1	3581
8835	-1	3575
8835	-1	3572
8835	-1	3570
8835	-1	3568
8835	-1	3566
8835	-1	3563
8835	-1	3561
8835	-1	3560
8835	-1	3559
8835	-1	3460
8835	-1	3459
8835	-1	3455
8835	-1	3454
8835	-1	2690
8835	-1	2057
8835	-1	163702
8835	-1	149233
8835	-1	1441
8835	-1	1439
8835	-1	1438
8835	-1	1271
8835	-1	116379
8835	-1	3643
8835	-1	8660
8835	-1	8471
8835	-1	3667
8651	-1	7297
8651	-1	3718
8651	-1	3717
8651	-1	3716
8651	-1	9180
8651	-1	64109
8651	-1	58985
8651	-1	5618
8651	-1	53833
8651	-1	53832
8651	-1	50615

8651	-1	4352
8651	-1	3977
8651	-1	3953
8651	-1	3601
8651	-1	3598
8651	-1	3597
8651	-1	3595
8651	-1	3594
8651	-1	3590
8651	-1	3588
8651	-1	3587
8651	-1	3581
8651	-1	3575
8651	-1	3572
8651	-1	3570
8651	-1	3568
8651	-1	3566
8651	-1	3563
8651	-1	3561
8651	-1	3560
8651	-1	3559
8651	-1	3460
8651	-1	3459
8651	-1	3455
8651	-1	3454
8651	-1	2690
8651	-1	2057
8651	-1	163702
8651	-1	149233
8651	-1	1441
8651	-1	1439
8651	-1	1438
8651	-1	1271
8651	-1	116379
8651	-1	3643
8651	-1	8660
8651	-1	8471
8651	-1	3667
122809	-1	7297
122809	-1	3718
122809	-1	3717
122809	-1	3716
122809	-1	9180
122809	-1	64109
122809	-1	58985
122809	-1	5618
122809	-1	53833
122809	-1	53832
122809	-1	50615
122809	-1	4352
122809	-1	3977
122809	-1	3953
122809	-1	3601
122809	-1	3598
122809	-1	3597

122809	-1	3595
122809	-1	3594
122809	-1	3590
122809	-1	3588
122809	-1	3587
122809	-1	3581
122809	-1	3575
122809	-1	3572
122809	-1	3570
122809	-1	3568
122809	-1	3566
122809	-1	3563
122809	-1	3561
122809	-1	3560
122809	-1	3559
122809	-1	3460
122809	-1	3459
122809	-1	3455
122809	-1	3454
122809	-1	2690
122809	-1	2057
122809	-1	163702
122809	-1	149233
122809	-1	1441
122809	-1	1439
122809	-1	1438
122809	-1	1271
122809	-1	116379
122809	-1	3643
122809	-1	8660
122809	-1	8471
122809	-1	3667
9021	-1	7297
9021	-1	3718
9021	-1	3717
9021	-1	3716
9021	-1	9180
9021	-1	64109
9021	-1	58985
9021	-1	5618
9021	-1	53833
9021	-1	53832
9021	-1	50615
9021	-1	4352
9021	-1	3977
9021	-1	3953
9021	-1	3601
9021	-1	3598
9021	-1	3597
9021	-1	3595
9021	-1	3594
9021	-1	3590
9021	-1	3588
9021	-1	3587
9021	-1	3581

9021	-1	3575
9021	-1	3572
9021	-1	3570
9021	-1	3568
9021	-1	3566
9021	-1	3563
9021	-1	3561
9021	-1	3560
9021	-1	3559
9021	-1	3460
9021	-1	3459
9021	-1	3455
9021	-1	3454
9021	-1	2690
9021	-1	2057
9021	-1	163702
9021	-1	149233
9021	-1	1441
9021	-1	1439
9021	-1	1438
9021	-1	1271
9021	-1	116379
9021	-1	3643
9021	-1	8660
9021	-1	8471
9021	-1	3667
434	-1	4157
7248	-1	6009
26999	-1	55845
26999	-1	8936
23191	-1	55845
23191	-1	8936
3071	-1	55845
3071	-1	8936
10787	-1	55845
10787	-1	8936
10152	-1	55845
10152	-1	8936
8396	-1	85477
8396	-1	2934
8395	-1	85477
8395	-1	2934
8394	-1	85477
8394	-1	2934
79837	-1	85477
79837	-1	2934
5305	-1	85477
5305	-1	2934
200576	-1	85477
200576	-1	2934
409	-1	8590
409	-1	8392
409	-1	8390
409	-1	8388
409	-1	8387

409	-1	8386
409	-1	8385
409	-1	8383
409	-1	81797
409	-1	81697
409	-1	81696
409	-1	81472
409	-1	81470
409	-1	81469
409	-1	81448
409	-1	81442
409	-1	81399
409	-1	81392
409	-1	81328
409	-1	81327
409	-1	81318
409	-1	81309
409	-1	81300
409	-1	81285
409	-1	81282
409	-1	81168
409	-1	81127
409	-1	81099
409	-1	81061
409	-1	81050
409	-1	79544
409	-1	79541
409	-1	79501
409	-1	79473
409	-1	79345
409	-1	79339
409	-1	79324
409	-1	7932
409	-1	79317
409	-1	79310
409	-1	79295
409	-1	79290
409	-1	56656
409	-1	504189
409	-1	4995
409	-1	4994
409	-1	4993
409	-1	4992
409	-1	4991
409	-1	442361
409	-1	442194
409	-1	442191
409	-1	442186
409	-1	441933
409	-1	441911
409	-1	441670
409	-1	441669
409	-1	441639
409	-1	441608
409	-1	402317

409	-1	402135
409	-1	401994
409	-1	401993
409	-1	401992
409	-1	401667
409	-1	401666
409	-1	401665
409	-1	401427
409	-1	393046
409	-1	392392
409	-1	392391
409	-1	392390
409	-1	392376
409	-1	392309
409	-1	392138
409	-1	391211
409	-1	391196
409	-1	391195
409	-1	391194
409	-1	391192
409	-1	391191
409	-1	391190
409	-1	391189
409	-1	391114
409	-1	391112
409	-1	391109
409	-1	391107
409	-1	390892
409	-1	390883
409	-1	390882
409	-1	390649
409	-1	390648
409	-1	390538
409	-1	390445
409	-1	390442
409	-1	390439
409	-1	390437
409	-1	390436
409	-1	390433
409	-1	390431
409	-1	390429
409	-1	390327
409	-1	390326
409	-1	390323
409	-1	390321
409	-1	390275
409	-1	390265
409	-1	390264
409	-1	390261
409	-1	390260
409	-1	390201
409	-1	390199
409	-1	390197
409	-1	390195
409	-1	390191

409	-1	390181
409	-1	390174
409	-1	390168
409	-1	390167
409	-1	390162
409	-1	390157
409	-1	390155
409	-1	390154
409	-1	390152
409	-1	390151
409	-1	390144
409	-1	390142
409	-1	390113
409	-1	390093
409	-1	390084
409	-1	390083
409	-1	390081
409	-1	390079
409	-1	390078
409	-1	390077
409	-1	390075
409	-1	390072
409	-1	390067
409	-1	390066
409	-1	390064
409	-1	390063
409	-1	390061
409	-1	390059
409	-1	390058
409	-1	390054
409	-1	390038
409	-1	390037
409	-1	390036
409	-1	389090
409	-1	387748
409	-1	347468
409	-1	347169
409	-1	347168
409	-1	346528
409	-1	346525
409	-1	346517
409	-1	343563
409	-1	343406
409	-1	343173
409	-1	343172
409	-1	343171
409	-1	343169
409	-1	341799
409	-1	341568
409	-1	341418
409	-1	341416
409	-1	341276
409	-1	341152
409	-1	340980
409	-1	338755

409	-1	338751
409	-1	338675
409	-1	338674
409	-1	338662
409	-1	286365
409	-1	286362
409	-1	285659
409	-1	284532
409	-1	284521
409	-1	284433
409	-1	284383
409	-1	283694
409	-1	283365
409	-1	283297
409	-1	283189
409	-1	283162
409	-1	283160
409	-1	283159
409	-1	283111
409	-1	283093
409	-1	283092
409	-1	282775
409	-1	282770
409	-1	282763
409	-1	26740
409	-1	26737
409	-1	26735
409	-1	26716
409	-1	26707
409	-1	26696
409	-1	26692
409	-1	26689
409	-1	26686
409	-1	26683
409	-1	26682
409	-1	26664
409	-1	26659
409	-1	26658
409	-1	26539
409	-1	26538
409	-1	26534
409	-1	26533
409	-1	26532
409	-1	26531
409	-1	26529
409	-1	26496
409	-1	26494
409	-1	26493
409	-1	26492
409	-1	26476
409	-1	26339
409	-1	26338
409	-1	26333
409	-1	26248
409	-1	26246

409	-1	26245
409	-1	26219
409	-1	26212
409	-1	26211
409	-1	26189
409	-1	26188
409	-1	256892
409	-1	256148
409	-1	256144
409	-1	255725
409	-1	254973
409	-1	254879
409	-1	254786
409	-1	254783
409	-1	23538
409	-1	219986
409	-1	219983
409	-1	219982
409	-1	219981
409	-1	219968
409	-1	219965
409	-1	219960
409	-1	219959
409	-1	219958
409	-1	219957
409	-1	219956
409	-1	219954
409	-1	219952
409	-1	219875
409	-1	219874
409	-1	219873
409	-1	219870
409	-1	219869
409	-1	219865
409	-1	219858
409	-1	219493
409	-1	219487
409	-1	219484
409	-1	219482
409	-1	219479
409	-1	219477
409	-1	219473
409	-1	219469
409	-1	219464
409	-1	219453
409	-1	219447
409	-1	219438
409	-1	219437
409	-1	219436
409	-1	219432
409	-1	219431
409	-1	219429
409	-1	219428
409	-1	219417
409	-1	196335

409	-1	162998
409	-1	158131
409	-1	150681
409	-1	144125
409	-1	144124
409	-1	143503
409	-1	143502
409	-1	143496
409	-1	138883
409	-1	138882
409	-1	138881
409	-1	138805
409	-1	138804
409	-1	138803
409	-1	138802
409	-1	138799
409	-1	135948
409	-1	135946
409	-1	135941
409	-1	135924
409	-1	134083
409	-1	130075
409	-1	128372
409	-1	128371
409	-1	128368
409	-1	128367
409	-1	128360
409	-1	127623
409	-1	127385
409	-1	127077
409	-1	127074
409	-1	127069
409	-1	127068
409	-1	127066
409	-1	127064
409	-1	127062
409	-1	127059
409	-1	126541
409	-1	126370
409	-1	125963
409	-1	125962
409	-1	125958
409	-1	124538
409	-1	122748
409	-1	122742
409	-1	122740
409	-1	121364
409	-1	121275
409	-1	121130
409	-1	120796
409	-1	120793
409	-1	120787
409	-1	120776
409	-1	120775
409	-1	120586

409	-1	120066
409	-1	120065
409	-1	119774
409	-1	119772
409	-1	119765
409	-1	119764
409	-1	119749
409	-1	119695
409	-1	119694
409	-1	119692
409	-1	119687
409	-1	119682
409	-1	119679
409	-1	119678
409	-1	10798
4659	-1	93408
4659	-1	58498
4659	-1	4636
4659	-1	4633
4659	-1	29895
4659	-1	10627
4659	-1	10398
4659	-1	103910
4659	-1	818
4659	-1	817
4659	-1	816
4659	-1	815
8864	-1	9575
8864	-1	4862
8864	-1	406
8863	-1	9575
8863	-1	4862
8863	-1	406
5187	-1	9575
5187	-1	4862
5187	-1	406
1454	-1	2737
1454	-1	2736
1454	-1	2735
1454	-1	9575
1454	-1	4862
1454	-1	406
1408	-1	9575
1408	-1	4862
1408	-1	406
1407	-1	9575
1407	-1	4862
1407	-1	406
6494	-1	5908
6494	-1	5906
7070	-1	394
7070	-1	2909
9063	-1	6778
9063	-1	6777
9063	-1	6776

9063	-1	6775
9063	-1	6774
9063	-1	6773
9063	-1	6772
8554	-1	6778
8554	-1	6777
8554	-1	6776
8554	-1	6775
8554	-1	6774
8554	-1	6773
8554	-1	6772
51588	-1	6778
51588	-1	6777
51588	-1	6776
51588	-1	6775
51588	-1	6774
51588	-1	6773
51588	-1	6772
10401	-1	6778
10401	-1	6777
10401	-1	6776
10401	-1	6775
10401	-1	6774
10401	-1	6773
10401	-1	6772
1154	-1	6778
1154	-1	6777
1154	-1	6776
1154	-1	6775
1154	-1	6774
1154	-1	6773
1154	-1	6772
5265	-1	5340
2	-1	2147
2	-1	5624
2	-1	5627
2	-1	5340
5104	-1	5624
5104	-1	5627
5104	-1	2147
462	-1	2147
462	-1	2159
462	-1	2158
462	-1	2160
5345	-1	5340
3053	-1	2147
7035	-1	2159
7035	-1	2153
7035	-1	2152
7035	-1	2155
5627	-1	2147
5627	-1	2157
5627	-1	2153
394	-1	387
2909	-1	387

29984	-1	27289
64221	-1	6091
5998	-1	2773
5998	-1	2771
5998	-1	2770
64750	-1	91
64750	-1	7046
64750	-1	130399
64750	-1	7048
57154	-1	91
57154	-1	7046
57154	-1	130399
57154	-1	7048
10468	-1	83729
10468	-1	3626
10468	-1	3625
10468	-1	3624
7044	-1	4838
7044	-1	7043
7044	-1	7042
7044	-1	7040
10637	-1	4838
10637	-1	7043
10637	-1	7042
10637	-1	7040
1634	-1	7043
1634	-1	7042
1634	-1	7040
4052	-1	7043
4052	-1	7042
4052	-1	7040
9241	-1	8200
9241	-1	656
9241	-1	655
9241	-1	654
9241	-1	653
9241	-1	652
9241	-1	650
9241	-1	392255
9241	-1	353500
9241	-1	268
9241	-1	151449
8646	-1	8200
8646	-1	656
8646	-1	655
8646	-1	654
8646	-1	653
8646	-1	652
8646	-1	650
8646	-1	392255
8646	-1	353500
8646	-1	268
8646	-1	151449
4092	-1	4093
4092	-1	4090

4092	-1	4086
4092	-1	4088
4092	-1	4087
4091	-1	4093
4091	-1	4090
4091	-1	4086
4091	-1	4088
4091	-1	4087
728622	-1	4088
728622	-1	4087
6500	-1	4088
6500	-1	4087
9978	-1	4088
9978	-1	4087
8454	-1	4088
8454	-1	4087
4609	-1	9063
4609	-1	8554
4609	-1	51588
4609	-1	10401
4609	-1	1030
8945	-1	2737
8945	-1	2736
8945	-1	2735
23291	-1	2737
23291	-1	2736
23291	-1	2735
9612	-1	3516
9612	-1	11317
9541	-1	3516
9541	-1	11317
9253	-1	4855
9253	-1	4854
9253	-1	4853
9253	-1	4851
8650	-1	4855
8650	-1	4854
8650	-1	4853
8650	-1	4851
3714	-1	4855
3714	-1	4854
3714	-1	4853
3714	-1	4851
182	-1	4855
182	-1	4854
182	-1	4853
182	-1	4851
57680	-1	1499
64321	-1	83439
64321	-1	6934
64321	-1	6932
64321	-1	51176
56998	-1	1499
59343	-1	1857
59343	-1	1856

59343	-1	1855
80319	-1	1857
80319	-1	1856
80319	-1	1855
11197	-1	89780
11197	-1	81029
11197	-1	80326
11197	-1	7484
11197	-1	7483
11197	-1	7482
11197	-1	7481
11197	-1	7480
11197	-1	7479
11197	-1	7478
11197	-1	7477
11197	-1	7476
11197	-1	7475
11197	-1	7474
11197	-1	7473
11197	-1	7472
11197	-1	7471
11197	-1	54361
11197	-1	51384
9350	-1	89780
9350	-1	81029
9350	-1	80326
9350	-1	7484
9350	-1	7483
9350	-1	7482
9350	-1	7481
9350	-1	7480
9350	-1	7479
9350	-1	7478
9350	-1	7477
9350	-1	7476
9350	-1	7475
9350	-1	7474
9350	-1	7473
9350	-1	7472
9350	-1	7471
9350	-1	54361
9350	-1	51384
6425	-1	8326
6425	-1	8325
6425	-1	8324
6425	-1	8323
6425	-1	8322
6425	-1	8321
6425	-1	7976
6425	-1	7855
6425	-1	2535
6425	-1	11211
6425	-1	89780
6425	-1	81029
6425	-1	80326

6425	-1	7484
6425	-1	7483
6425	-1	7482
6425	-1	7481
6425	-1	7480
6425	-1	7479
6425	-1	7478
6425	-1	7477
6425	-1	7476
6425	-1	7475
6425	-1	7474
6425	-1	7473
6425	-1	7472
6425	-1	7471
6425	-1	54361
6425	-1	51384
6424	-1	8326
6424	-1	8325
6424	-1	8324
6424	-1	8323
6424	-1	8322
6424	-1	8321
6424	-1	7976
6424	-1	7855
6424	-1	2535
6424	-1	11211
6424	-1	89780
6424	-1	81029
6424	-1	80326
6424	-1	7484
6424	-1	7483
6424	-1	7482
6424	-1	7481
6424	-1	7480
6424	-1	7479
6424	-1	7478
6424	-1	7477
6424	-1	7476
6424	-1	7475
6424	-1	7474
6424	-1	7473
6424	-1	7472
6424	-1	7471
6424	-1	54361
6424	-1	51384
6423	-1	[illegible]
6423	-1	8325
6423	-1	8324
6423	-1	8323
6423	-1	8322
6423	-1	8321
6423	-1	7976
6423	-1	7855
6423	-1	2535
6423	-1	11211

6423	-1	89780
6423	-1	81029
6423	-1	80326
6423	-1	7484
6423	-1	7483
6423	-1	7482
6423	-1	7481
6423	-1	7480
6423	-1	7479
6423	-1	7478
6423	-1	7477
6423	-1	7476
6423	-1	7475
6423	-1	7474
6423	-1	7473
6423	-1	7472
6423	-1	7471
6423	-1	54361
6423	-1	51384
6422	-1	8326
6422	-1	8325
6422	-1	8324
6422	-1	8323
6422	-1	8322
6422	-1	8321
6422	-1	7976
6422	-1	7855
6422	-1	2535
6422	-1	11211
6422	-1	89780
6422	-1	81029
6422	-1	80326
6422	-1	7484
6422	-1	7483
6422	-1	7482
6422	-1	7481
6422	-1	7480
6422	-1	7479
6422	-1	7478
6422	-1	7477
6422	-1	7476
6422	-1	7475
6422	-1	7474
6422	-1	7473
6422	-1	7472
6422	-1	7471
6422	-1	54361
6422	-1	51384
8837	-1	8717
8837	-1	8772
596	-1	7157
331	-1	842
331	-1	840
331	-1	836
331	-1	839

330	-1	842
330	-1	840
330	-1	836
330	-1	839
329	-1	842
329	-1	840
329	-1	836
329	-1	839
7249	-1	6009
1978	-1	9470
1978	-1	253314
1978	-1	1977
3486	-1	3479
2810	-1	983
2810	-1	9133
2810	-1	891
2810	-1	85417
1032	-1	896
1032	-1	894
1032	-1	595
1032	-1	1021
1032	-1	1019
1031	-1	896
1031	-1	894
1031	-1	595
1031	-1	1021
1031	-1	1019
1030	-1	896
1030	-1	894
1030	-1	595
1030	-1	1021
1030	-1	1019
1029	-1	4193
1029	-1	896
1029	-1	894
1029	-1	595
1029	-1	1021
1029	-1	1019
9134	-1	5925
9134	-1	1028
9134	-1	1027
898	-1	5925
898	-1	1028
898	-1	1027
1026	-1	8900
1026	-1	890
1026	-1	9134
1026	-1	898
1026	-1	1017
1026	-1	896
1026	-1	894
1026	-1	595
1026	-1	1021
1026	-1	1019
8900	-1	1871

8900	-1	1870
8900	-1	1869
8900	-1	990
8900	-1	5925
890	-1	1871
890	-1	1870
890	-1	1869
890	-1	990
890	-1	5925
1017	-1	1871
1017	-1	1870
1017	-1	1869
1017	-1	990
1017	-1	5925
1017	-1	1028
1017	-1	1027
1028	-1	8900
1028	-1	890
1028	-1	1017
1028	-1	896
1028	-1	894
1028	-1	595
1028	-1	1021
1028	-1	1019
896	-1	5934
896	-1	5933
896	-1	5925
894	-1	5934
894	-1	5933
894	-1	5925
595	-1	5934
595	-1	5933
595	-1	5925
1021	-1	5934
1021	-1	5933
1021	-1	5925
1019	-1	5934
1019	-1	5933
1019	-1	5925
1027	-1	9134
1027	-1	898
1027	-1	8900
1027	-1	890
1027	-1	1017
1027	-1	896
1027	-1	894
1027	-1	595
1027	-1	1021
1027	-1	1019
5350	-1	489
5350	-1	488
5350	-1	487
5350	-1	1E+08
3312	-1	5602
3312	-1	5601

3312	-1	5599
3310	-1	5602
3310	-1	5601
3310	-1	5599
3306	-1	5602
3306	-1	5601
3306	-1	5599
3305	-1	5602
3305	-1	5601
3305	-1	5599
3304	-1	5602
3304	-1	5601
3304	-1	5599
3303	-1	5602
3303	-1	5601
3303	-1	5599
4763	-1	6237
4763	-1	4893
4763	-1	3845
4763	-1	3265
4763	-1	22808
4763	-1	22800
3784	1	115
3784	1	109
3784	1	6558
3784	1	5616
3784	1	5613
3784	1	5568
3784	1	5567
3784	1	5566
6558	1	115
6558	1	109
6558	1	5616
6558	1	5613
6558	1	5568
6558	1	5567
6558	1	5566
90993	-1	2033
90993	-1	1387
84699	-1	2033
84699	-1	1387
64764	-1	2033
64764	-1	1387
148327	-1	2033
148327	-1	1387
10488	-1	2033
10488	-1	1387
4215	1	650832
4215	1	5608
4215	1	5606
468	-1	2033
468	-1	1387
8569	1	9470
8569	1	253314
8569	1	1977

2872	1	9470
2872	1	253314
2872	1	1977
5577	-1	572
5577	1	3991
5576	-1	572
5576	1	3991
5575	-1	572
5575	1	3991
5573	-1	572
5573	1	3991
5261	1	5837
5261	1	5836
5261	1	5834
5260	1	5837
5260	1	5836
5260	1	5834
5257	1	5837
5257	1	5836
5257	1	5834
5256	1	5837
5256	1	5836
5256	1	5834
5255	1	5837
5255	1	5836
5255	1	5834
5502	-1	5501
5502	-1	5500
5502	-1	5499
5502	-1	4659
695	1	5335
3932	1	7305
1453	-1	2737
1453	-1	2736
1453	-1	2735
1453	-1	9575
1453	-1	4862
1453	-1	406
1453	1	2697
5590	-1	8660
5590	-1	8471
5590	-1	3667
5590	1	56288
5584	1	56288
2241	-1	1500
2241	-1	1499
7525	-1	1500
7525	1	2241
7525	1	2534
1460	1	1499
1459	1	1499
1457	1	1499
3985	-1	1073
3985	-1	1072
3984	-1	1073

3984	-1	1072
2242	1	55558
2242	1	5362
2242	1	5361
2242	1	1808
2932	-1	9586
2932	-1	90993
2932	-1	84699
2932	-1	64764
2932	-1	468
2932	-1	148327
2932	-1	1385
2932	-1	10488
2932	-1	2998
2932	-1	2997
2932	-1	4776
2932	-1	4775
2932	-1	4773
2932	-1	4772
2932	-1	10725
2932	-1	2737
2932	-1	2736
2932	-1	2735
2932	-1	1499
2932	-1	896
2932	-1	894
2932	-1	595
2932	1	1808
983	-1	57369
983	-1	2697
1022	1	983
5605	1	5294
5605	1	5293
5605	1	5291
5605	1	5290
5604	-1	572
5604	1	5294
5604	1	5293
5604	1	5291
5604	1	5290
5604	1	5602
5604	1	5601
5604	1	5599
79660	-1	810
79660	-1	808
79660	-1	805
79660	-1	801
79660	-1	5261
79660	-1	5260
79660	-1	5257
79660	-1	5256
79660	-1	5255
79660	-1	51806
79660	-1	163688
79660	1	2998

79660	1	2997
5509	-1	810
5509	-1	808
5509	-1	805
5509	-1	801
5509	-1	5261
5509	-1	5260
5509	-1	5257
5509	-1	5256
5509	-1	5255
5509	-1	51806
5509	-1	163688
5509	1	2998
5509	1	2997
5507	-1	810
5507	-1	808
5507	-1	805
5507	-1	801
5507	-1	5261
5507	-1	5260
5507	-1	5257
5507	-1	5256
5507	-1	5255
5507	-1	51806
5507	-1	163688
5507	1	2998
5507	1	2997
5506	-1	810
5506	-1	808
5506	-1	805
5506	-1	801
5506	-1	5261
5506	-1	5260
5506	-1	5257
5506	-1	5256
5506	-1	5255
5506	-1	51806
5506	-1	163688
5506	1	2998
5506	1	2997
5501	-1	810
5501	-1	808
5501	-1	805
5501	-1	801
5501	-1	5261
5501	-1	5260
5501	-1	5257
5501	-1	5256
5501	-1	5255
5501	-1	51806
5501	-1	163688
5501	-1	93408
5501	-1	58498
5501	-1	4636
5501	-1	4633

5501	-1	29895
5501	-1	10627
5501	-1	10398
5501	-1	103910
5501	-1	818
5501	-1	817
5501	-1	816
5501	-1	815
5501	1	2998
5501	1	2997
5500	-1	810
5500	-1	808
5500	-1	805
5500	-1	801
5500	-1	5261
5500	-1	5260
5500	-1	5257
5500	-1	5256
5500	-1	5255
5500	-1	51806
5500	-1	163688
5500	-1	93408
5500	-1	58498
5500	-1	4636
5500	-1	4633
5500	-1	29895
5500	-1	10627
5500	-1	10398
5500	-1	103910
5500	-1	818
5500	-1	817
5500	-1	816
5500	-1	815
5500	1	2998
5500	1	2997
5499	-1	810
5499	-1	808
5499	-1	805
5499	-1	801
5499	-1	5261
5499	-1	5260
5499	-1	5257
5499	-1	5256
5499	-1	5255
5499	-1	51806
5499	-1	163688
5499	-1	93408
5499	-1	58498
5499	-1	4636
5499	-1	4633
5499	-1	29895
5499	-1	10627
5499	-1	10398
5499	-1	103910
5499	-1	818

5499	-1	817
5499	-1	816
5499	-1	815
5499	1	2998
5499	1	2997
5795	1	1500
5770	-1	8660
5770	-1	8471
5770	-1	3667
5770	-1	3643
5770	1	1499
5792	-1	8660
5792	-1	8471
5792	-1	3667
5792	-1	3643
5792	1	1499
5787	1	1499
5797	1	1500
5797	1	1499
52	1	1499
572	-1	598
5170	1	6199
5170	1	6198
208	-1	2309
208	-1	2308
208	-1	842
208	-1	7249
208	-1	1026
208	-1	1027
208	-1	2932
208	-1	572
208	-1	6416
208	-1	4217
208	1	4193
208	1	5140
208	1	5139
208	1	4846
208	1	8517
208	1	3551
208	1	1147
208	1	2475
208	1	598
207	-1	2309
207	-1	2308
207	-1	842
207	-1	7249
207	-1	1026
207	-1	1027
207	-1	2932
207	-1	572
207	-1	6416
207	-1	4217
207	1	4193
207	1	5140
207	1	5139

207	1	4846
207	1	8517
207	1	3551
207	1	1147
207	1	2475
207	1	598
10000	-1	2309
10000	-1	2308
10000	-1	842
10000	-1	7249
10000	-1	1026
10000	-1	1027
10000	-1	2932
10000	-1	572
10000	-1	6416
10000	-1	4217
10000	1	4193
10000	1	5140
10000	1	5139
10000	1	4846
10000	1	8517
10000	1	3551
10000	1	1147
10000	1	2475
10000	1	598
861	-1	1050
861	-1	6688
861	1	3066
861	1	3065
861	1	1488
861	1	1487
3066	-1	3516
3066	-1	11317
3065	-1	3516
3065	-1	11317
1488	-1	3516
1488	-1	11317
1488	-1	83439
1488	-1	6934
1488	-1	6932
1488	-1	51176
1487	-1	3516
1487	-1	11317
1487	-1	83439
1487	-1	6934
1487	-1	6932
1487	-1	51176
2122	-1	5602
2122	-1	5601
2122	-1	5599
2122	1	3066
2122	1	3065
2122	1	1488
2122	1	1487
10928	-1	998

10928	-1	5881
10928	-1	5880
10928	-1	5879
5899	1	5337
5899	1	10928
5898	1	5337
5898	1	10928
5900	1	5881
5900	1	5880
5900	1	5879
5900	1	387
5900	1	5899
5900	1	5898
613	1	6464
613	1	53358
613	1	399694
613	1	25759
613	1	868
613	1	867
613	1	23624
613	1	1399
613	1	1398
613	1	2885
613	1	6777
613	1	6776
868	-1	7535
868	-1	2534
868	-1	3932
868	-1	7297
868	-1	3718
868	-1	3717
868	-1	3716
868	-1	9180
868	-1	64109
868	-1	58985
868	-1	5618
868	-1	53833
868	-1	53832
868	-1	50615
868	-1	4352
868	-1	3977
868	-1	3953
868	-1	3601
868	-1	3598
868	-1	3597
868	-1	3595
868	-1	3594
868	-1	3590
868	-1	3588
868	-1	3587
868	-1	3581
868	-1	3575
868	-1	3572
868	-1	3570
868	-1	3568

868	-1	3566
868	-1	3563
868	-1	3561
868	-1	3560
868	-1	3559
868	-1	3460
868	-1	3459
868	-1	3455
868	-1	3454
868	-1	2690
868	-1	2057
868	-1	163702
868	-1	149233
868	-1	1441
868	-1	1439
868	-1	1438
868	-1	1271
868	-1	116379
868	1	8503
868	1	5296
868	1	5295
868	1	5294
868	1	5293
868	1	5291
868	1	5290
868	1	23533
867	-1	7535
867	-1	2534
867	-1	3932
867	-1	7297
867	-1	3718
867	-1	3717
867	-1	3716
867	-1	9180
867	-1	64109
867	-1	58985
867	-1	5618
867	-1	53833
867	-1	53832
867	-1	50615
867	-1	4352
867	-1	3977
867	-1	3953
867	-1	3601
867	-1	3598
867	-1	3597
867	-1	3595
867	-1	3594
867	-1	3590
867	-1	3588
867	-1	3587
867	-1	3581
867	-1	3575
867	-1	3572
867	-1	3570

867	-1	3568
867	-1	3566
867	-1	3563
867	-1	3561
867	-1	3560
867	-1	3559
867	-1	3460
867	-1	3459
867	-1	3455
867	-1	3454
867	-1	2690
867	-1	2057
867	-1	163702
867	-1	149233
867	-1	1441
867	-1	1439
867	-1	1438
867	-1	1271
867	-1	116379
867	1	8503
867	1	5296
867	1	5295
867	1	5294
867	1	5293
867	1	5291
867	1	5290
867	1	23533
23624	-1	7535
23624	-1	2534
23624	-1	3932
23624	-1	7297
23624	-1	3718
23624	-1	3717
23624	-1	3716
23624	-1	9180
23624	-1	64109
23624	-1	58985
23624	-1	5618
23624	-1	53833
23624	-1	53832
23624	-1	50615
23624	-1	4352
23624	-1	3977
23624	-1	3953
23624	-1	3601
23624	-1	3598
23624	-1	3597
23624	-1	3595
23624	-1	3594
23624	-1	3590
23624	-1	3588
23624	-1	3587
23624	-1	3581
23624	-1	3575
23624	-1	3572

23624	-1	3570
23624	-1	3568
23624	-1	3566
23624	-1	3563
23624	-1	3561
23624	-1	3560
23624	-1	3559
23624	-1	3460
23624	-1	3459
23624	-1	3455
23624	-1	3454
23624	-1	2690
23624	-1	2057
23624	-1	163702
23624	-1	149233
23624	-1	1441
23624	-1	1439
23624	-1	1438
23624	-1	1271
23624	-1	116379
23624	1	8503
23624	1	5296
23624	1	5295
23624	1	5294
23624	1	5293
23624	1	5291
23624	1	5290
23624	1	23533
9181	1	387
102	1	1839
8517	-1	8660
8517	-1	8471
8517	-1	3667
8517	1	4792
1906	1	1910
4157	1	2778
5443	1	4157
4313	1	1839
2797	1	2798
2798	1	2776
2798	1	2767
2798	1	2778
2796	1	2798
8660	1	8503
8660	1	5296
8660	1	5295
8660	1	5294
8660	1	5293
8660	1	5291
8660	1	5290
8660	1	23533
8660	1	2885
8471	1	8503
8471	1	5296
8471	1	5295

8471	1	5294
8471	1	5293
8471	1	5291
8471	1	5290
8471	1	23533
8471	1	2885
3667	1	8503
3667	1	5296
3667	1	5295
3667	1	5294
3667	1	5293
3667	1	5291
3667	1	5290
3667	1	23533
3667	1	2885
3643	1	8660
3643	1	8471
3643	1	3667
3643	1	6464
3643	1	53358
3643	1	399694
3643	1	25759
3643	1	868
3643	1	867
3643	1	23624
3643	1	10603
3630	1	5159
3630	1	5156
3630	1	3645
3630	1	3480
3630	1	2263
3630	1	2260
3630	1	2064
3630	1	1956
3630	1	3643
7074	1	5881
7074	1	5880
7074	1	5879
26230	1	5881
26230	1	5880
26230	1	5879
28964	-1	5829
8874	-1	9138
8874	-1	23365
8874	1	5881
8874	1	5880
8874	1	5879
8874	1	28964
55845	1	81873
55845	1	653888
55845	1	10552
55845	1	10109
55845	1	10095
55845	1	10094
55845	1	10093

55845	1	10092
324	1	50649
50649	1	5881
50649	1	5880
50649	1	5879
10297	1	50649
81873	1	71
81873	1	648921
81873	1	646821
81873	1	646048
81873	1	60
10092	1	71
10092	1	648921
10092	1	646821
10092	1	646048
10092	1	60
81624	1	10458
89846	1	998
2245	1	998
1132	1	55970
3687	1	5747
3687	1	9564
3687	1	1399
3687	1	1398
3687	1	1445
3682	1	5747
3682	1	9564
3682	1	1399
3682	1	1398
3682	1	1445
3681	1	5747
3681	1	9564
3681	1	1399
3681	1	1398
3681	1	1445
1445	-1	6714
1445	-1	4067
9459	-1	9138
9459	-1	23365
9459	1	5881
9459	1	5880
9459	1	5879
9459	1	28964
3645	1	2885
3645	1	8503
3645	1	5296
3645	1	5295
3645	1	5294
3645	1	5293
3645	1	5291
3645	1	5290
3645	1	23533
3645	1	6655
3645	1	6654
1730	1	5217

1730	1	5216
1730	1	375189
1730	1	345456
9138	1	387
80834	1	2778
80834	1	346562
5726	1	346562
54429	1	346562
50840	1	346562
50839	1	346562
50838	1	346562
50837	1	346562
50836	1	346562
50835	1	346562
50834	1	346562
50833	1	346562
50832	1	346562
50831	1	346562
353164	1	346562
338398	1	346562
259296	1	346562
259295	1	346562
259294	1	346562
259292	1	346562
259291	1	346562
259290	1	346562
259289	1	346562
259287	1	346562
259286	1	346562
259285	1	346562
51764	1	196883
51764	1	114
51764	1	112
51764	1	5330
2785	1	196883
2785	1	114
2785	1	112
2785	1	5330
2784	1	196883
2784	1	114
2784	1	112
2784	1	5330
2782	1	196883
2782	1	114
2782	1	112
2782	1	5330
80835	1	346562
346562	1	5136
83756	1	2778
83756	1	346562
8590	1	2774
8392	1	2774
8390	1	2774
8388	1	2774
8387	1	2774

8386	1	2774
8385	1	2774
8383	1	2774
81797	1	2774
81697	1	2774
81696	1	2774
81472	1	2774
81470	1	2774
81469	1	2774
81448	1	2774
81442	1	2774
81399	1	2774
81392	1	2774
81328	1	2774
81327	1	2774
81318	1	2774
81309	1	2774
81300	1	2774
81285	1	2774
81282	1	2774
81168	1	2774
81127	1	2774
81099	1	2774
81061	1	2774
81050	1	2774
79544	1	2774
79541	1	2774
79501	1	2774
79473	1	2774
79345	1	2774
79339	1	2774
79324	1	2774
7932	1	2774
79317	1	2774
79310	1	2774
79295	1	2774
79290	1	2774
56656	1	2774
504189	1	2774
4995	1	2774
4994	1	2774
4993	1	2774
4992	1	2774
4991	1	2774
442361	1	2774
442194	1	2774
442191	1	2774
442186	1	2774
441933	1	2774
441911	1	2774
441670	1	2774
441669	1	2774
441639	1	2774
441608	1	2774
402317	1	2774

402135	1	2774
401994	1	2774
401993	1	2774
401992	1	2774
401667	1	2774
401666	1	2774
401665	1	2774
401427	1	2774
393046	1	2774
392392	1	2774
392391	1	2774
392390	1	2774
392376	1	2774
392309	1	2774
392138	1	2774
391211	1	2774
391196	1	2774
391195	1	2774
391194	1	2774
391192	1	2774
391191	1	2774
391190	1	2774
391189	1	2774
391114	1	2774
391112	1	2774
391109	1	2774
391107	1	2774
390892	1	2774
390883	1	2774
390882	1	2774
390649	1	2774
390648	1	2774
390538	1	2774
390445	1	2774
390442	1	2774
390439	1	2774
390437	1	2774
390436	1	2774
390433	1	2774
390431	1	2774
390429	1	2774
390327	1	2774
390326	1	2774
390323	1	2774
390321	1	2774
390275	1	2774
390265	1	2774
390264	1	2774
390261	1	2774
390260	1	2774
390201	1	2774
390199	1	2774
390197	1	2774
390195	1	2774
390191	1	2774

390181	1	2774
390174	1	2774
390168	1	2774
390167	1	2774
390162	1	2774
390157	1	2774
390155	1	2774
390154	1	2774
390152	1	2774
390151	1	2774
390144	1	2774
390142	1	2774
390113	1	2774
390093	1	2774
390084	1	2774
390083	1	2774
390081	1	2774
390079	1	2774
390078	1	2774
390077	1	2774
390075	1	2774
390072	1	2774
390067	1	2774
390066	1	2774
390064	1	2774
390063	1	2774
390061	1	2774
390059	1	2774
390058	1	2774
390054	1	2774
390038	1	2774
390037	1	2774
390036	1	2774
389090	1	2774
387748	1	2774
347468	1	2774
347169	1	2774
347168	1	2774
346528	1	2774
346525	1	2774
346517	1	2774
343563	1	2774
343406	1	2774
343173	1	2774
343172	1	2774
343171	1	2774
343169	1	2774
341799	1	2774
341568	1	2774
341418	1	2774
341416	1	2774
341276	1	2774
341152	1	2774
340980	1	2774
338755	1	2774

338751	1	2774
338675	1	2774
338674	1	2774
338662	1	2774
286365	1	2774
286362	1	2774
285659	1	2774
284532	1	2774
284521	1	2774
284433	1	2774
284383	1	2774
283694	1	2774
283365	1	2774
283297	1	2774
283189	1	2774
283162	1	2774
283160	1	2774
283159	1	2774
283111	1	2774
283093	1	2774
283092	1	2774
282775	1	2774
282770	1	2774
282763	1	2774
26740	1	2774
26737	1	2774
26735	1	2774
26716	1	2774
26707	1	2774
26696	1	2774
26692	1	2774
26689	1	2774
26686	1	2774
26683	1	2774
26682	1	2774
26664	1	2774
26659	1	2774
26658	1	2774
26539	1	2774
26538	1	2774
26534	1	2774
26533	1	2774
26532	1	2774
26531	1	2774
26529	1	2774
26496	1	2774
26494	1	2774
26493	1	2774
26492	1	2774
26476	1	2774
26339	1	2774
26338	1	2774
26333	1	2774
26248	1	2774
26246	1	2774

26245	1	2774
26219	1	2774
26212	1	2774
26211	1	2774
26189	1	2774
26188	1	2774
256892	1	2774
256148	1	2774
256144	1	2774
255725	1	2774
254973	1	2774
254879	1	2774
254786	1	2774
254783	1	2774
23538	1	2774
219986	1	2774
219983	1	2774
219982	1	2774
219981	1	2774
219968	1	2774
219965	1	2774
219960	1	2774
219959	1	2774
219958	1	2774
219957	1	2774
219956	1	2774
219954	1	2774
219952	1	2774
219875	1	2774
219874	1	2774
219873	1	2774
219870	1	2774
219869	1	2774
219865	1	2774
219858	1	2774
219493	1	2774
219487	1	2774
219484	1	2774
219482	1	2774
219479	1	2774
219477	1	2774
219473	1	2774
219469	1	2774
219464	1	2774
219453	1	2774
219447	1	2774
219438	1	2774
219437	1	2774
219436	1	2774
219432	1	2774
219431	1	2774
219429	1	2774
219428	1	2774
219417	1	2774
196335	1	2774

162998	1	2774
158131	1	2774
150681	1	2774
144125	1	2774
144124	1	2774
143503	1	2774
143502	1	2774
143496	1	2774
138883	1	2774
138882	1	2774
138881	1	2774
138805	1	2774
138804	1	2774
138803	1	2774
138802	1	2774
138799	1	2774
135948	1	2774
135946	1	2774
135941	1	2774
135924	1	2774
134083	1	2774
130075	1	2774
128372	1	2774
128371	1	2774
128368	1	2774
128367	1	2774
128360	1	2774
127623	1	2774
127385	1	2774
127077	1	2774
127074	1	2774
127069	1	2774
127068	1	2774
127066	1	2774
127064	1	2774
127062	1	2774
127059	1	2774
126541	1	2774
126370	1	2774
125963	1	2774
125962	1	2774
125958	1	2774
124538	1	2774
122748	1	2774
122742	1	2774
122740	1	2774
121364	1	2774
121275	1	2774
121130	1	2774
120796	1	2774
120793	1	2774
120787	1	2774
120776	1	2774
120775	1	2774
120586	1	2774

120066	1	2774
120065	1	2774
119774	1	2774
119772	1	2774
119765	1	2774
119764	1	2774
119749	1	2774
119695	1	2774
119694	1	2774
119692	1	2774
119687	1	2774
119682	1	2774
119679	1	2774
119678	1	2774
10798	1	2774
1394	1	2781
1394	1	2778
1394	1	2775
1394	1	2773
1394	1	2771
1394	1	2770
1394	1	2768
1394	1	10672
1392	1	1394
2892	1	4067
2891	1	4067
4067	1	2207
4067	1	2206
4067	1	5582
4067	1	5579
4067	1	5578
2890	1	4067
2781	1	8681
2781	1	84647
2781	1	8399
2781	1	8398
2781	1	81579
2781	1	64600
2781	1	5322
2781	1	5321
2781	1	5320
2781	1	5319
2781	1	50487
2781	1	30814
2781	1	26279
2781	1	1E+08
2775	1	1857
2775	1	1856
2775	1	1855
2775	1	5332
2775	1	5331
2775	1	5330
2775	1	23236
2775	1	8681
2775	1	84647

2775	1	8399
2775	1	8398
2775	1	81579
2775	1	64600
2775	1	5322
2775	1	5321
2775	1	5320
2775	1	5319
2775	1	50487
2775	1	30814
2775	1	26279
2775	1	1E+08
10672	1	9138
10672	1	23365
10672	1	8681
10672	1	84647
10672	1	8399
10672	1	8398
10672	1	81579
10672	1	64600
10672	1	5322
10672	1	5321
10672	1	5320
10672	1	5319
10672	1	50487
10672	1	30814
10672	1	26279
10672	1	1E+08
5593	1	109
5593	1	10842
10842	-1	5519
10842	-1	5518
10842	-1	5516
10842	-1	5515
5592	1	109
5592	1	10842
814	1	2033
814	1	1387
818	-1	109
818	1	2891
818	1	2890
818	1	2915
818	1	2911
817	-1	109
817	1	2891
817	1	2890
817	1	2915
817	1	2911
816	-1	109
816	1	2891
816	1	2890
816	1	2915
816	1	2911
815	-1	109
815	1	2891

815	1	2890
815	1	2915
815	1	2911
5616	-1	2737
5616	-1	2736
5616	-1	2735
5616	-1	572
5616	-1	5350
5616	-1	3745
5616	1	1080
5616	1	115
5616	1	109
5616	1	90993
5616	1	84699
5616	1	64764
5616	1	148327
5616	1	1385
5616	1	10488
5616	1	2033
5616	1	1387
5616	1	468
5616	1	3991
5616	1	5132
5616	1	5502
5616	1	2891
5616	1	2890
5616	1	5595
5616	1	5594
5613	-1	2737
5613	-1	2736
5613	-1	2735
5613	-1	572
5613	-1	5350
5613	-1	3745
5613	1	1080
5613	1	115
5613	1	109
5613	1	90993
5613	1	84699
5613	1	64764
5613	1	148327
5613	1	1385
5613	1	10488
5613	1	2033
5613	1	1387
5613	1	468
5613	1	3991
5613	1	5132
5613	1	5502
5613	1	2891
5613	1	2890
5613	1	5595
5613	1	5594
5568	-1	2737
5568	-1	2736

5568	-1	2735
5568	-1	572
5568	-1	5350
5568	-1	3745
5568	1	1080
5568	1	115
5568	1	109
5568	1	90993
5568	1	84699
5568	1	64764
5568	1	148327
5568	1	1385
5568	1	10488
5568	1	2033
5568	1	1387
5568	1	468
5568	1	3991
5568	1	5132
5568	1	5502
5568	1	2891
5568	1	2890
5568	1	5595
5568	1	5594
5567	-1	2737
5567	-1	2736
5567	-1	2735
5567	-1	572
5567	-1	5350
5567	-1	3745
5567	1	1080
5567	1	115
5567	1	109
5567	1	90993
5567	1	84699
5567	1	64764
5567	1	148327
5567	1	1385
5567	1	10488
5567	1	2033
5567	1	1387
5567	1	468
5567	1	3991
5567	1	5132
5567	1	5502
5567	1	2891
5567	1	2890
5567	1	5595
5567	1	5594
5595	-1	9475
5595	-1	6093
5595	-1	57369
5595	-1	2697
5595	-1	4093
5595	-1	4090
5595	-1	4086

5595	-1	4088
5595	-1	4087
5595	-1	4089
5595	-1	7249
5595	1	9252
5595	1	2113
5595	1	4286
5595	1	3725
5595	1	2353
5594	-1	9475
5594	-1	6093
5594	-1	57369
5594	-1	2697
5594	-1	4093
5594	-1	4090
5594	-1	4086
5594	-1	4088
5594	-1	4087
5594	-1	4089
5594	-1	7249
5594	1	9252
5594	1	2113
5594	1	4286
5594	1	3725
5594	1	2353
5566	-1	2737
5566	-1	2736
5566	-1	2735
5566	-1	572
5566	-1	5350
5566	-1	3745
5566	1	1080
5566	1	115
5566	1	109
5566	1	90993
5566	1	84699
5566	1	64764
5566	1	148327
5566	1	1385
5566	1	10488
5566	1	2033
5566	1	1387
5566	1	468
5566	1	3991
5566	1	5132
5566	1	5502
5566	1	2891
5566	1	2890
5566	1	5595
5566	1	5594
7294	1	7410
7294	1	7409
7294	1	10451
11069	1	83593
11069	1	5908

11069	1	5906
10411	1	83593
10411	1	5908
10411	1	5906
83593	1	3684
83593	1	3689
83593	1	3683
83593	1	3676
83593	1	3688
9846	1	5781
9846	1	8503
9846	1	5296
9846	1	5295
9846	1	5294
9846	1	5293
9846	1	5291
9846	1	5290
9846	1	23533
5609	1	6885
930	1	7410
930	1	7409
930	1	10451
930	1	8503
930	1	5296
930	1	5295
930	1	5294
930	1	5293
930	1	5291
930	1	5290
930	1	23533
84433	1	8517
84433	1	1147
84433	1	3551
8915	1	8517
8915	1	1147
8915	1	3551
1147	-1	8660
1147	-1	8471
1147	-1	3667
1147	1	4792
3551	-1	8660
3551	-1	8471
3551	-1	3667
3551	1	4792
10892	1	8517
10892	1	1147
10892	1	3551
27040	1	5336
27040	1	5335
27040	1	2885
3937	1	7410
3937	1	7409
3937	1	10451
3937	1	5336
3937	1	5335

3937	1	2885
10870	1	2885
10870	1	8503
10870	1	5296
10870	1	5295
10870	1	5294
10870	1	5293
10870	1	5291
10870	1	5290
10870	1	23533
10870	1	5336
10870	1	5335
919	1	6850
919	1	7535
2207	1	6850
2207	1	7535
6850	1	7410
6850	1	7409
6850	1	10451
6850	1	5335
6850	1	27040
6850	1	3937
6850	1	9846
6850	1	6464
6850	1	53358
6850	1	399694
6850	1	25759
6850	1	8503
6850	1	5296
6850	1	5295
6850	1	5294
6850	1	5293
6850	1	5291
6850	1	5290
6850	1	23533
7535	1	6300
7535	1	5603
7535	1	5600
7535	1	1432
7535	1	27040
7535	1	3937
7535	1	6464
7535	1	53358
7535	1	399694
7535	1	25759
7535	1	8503
7535	1	5296
7535	1	5295
7535	1	5294
7535	1	5293
7535	1	5291
7535	1	5290
7535	1	23533
7305	1	6850
7305	1	7535

4068	1	2534
2185	1	5881
2185	1	5880
2185	1	5879
5781	-1	27040
5781	-1	3937
5781	-1	7410
5781	-1	7409
5781	-1	10451
5777	-1	6850
5777	-1	695
5777	-1	919
5777	-1	7535
5777	-1	27040
5777	-1	3937
5777	-1	7410
5777	-1	7409
5777	-1	10451
5777	-1	7297
5777	-1	3718
5777	-1	3717
5777	-1	3716
5777	-1	9180
5777	-1	64109
5777	-1	58985
5777	-1	5618
5777	-1	53833
5777	-1	53832
5777	-1	50615
5777	-1	4352
5777	-1	3977
5777	-1	3953
5777	-1	3601
5777	-1	3598
5777	-1	3597
5777	-1	3595
5777	-1	3594
5777	-1	3590
5777	-1	3588
5777	-1	3587
5777	-1	3581
5777	-1	3575
5777	-1	3572
5777	-1	3570
5777	-1	3568
5777	-1	3566
5777	-1	3563
5777	-1	3561
5777	-1	3560
5777	-1	3559
5777	-1	3460
5777	-1	3459
5777	-1	3455
5777	-1	3454
5777	-1	2690

5777	-1	2057
5777	-1	163702
5777	-1	149233
5777	-1	1441
5777	-1	1439
5777	-1	1438
5777	-1	1271
5777	-1	116379
5777	1	1500
3002	1	637
3002	1	836
51744	1	27040
51744	1	4068
51744	1	117157
962	1	51744
8027	1	7297
8027	1	3718
8027	1	3717
8027	1	3716
10254	1	7297
10254	1	3718
10254	1	3717
10254	1	3716
9641	1	3661
9641	1	3665
29110	1	3661
29110	1	3665
7187	1	9641
7187	1	29110
7098	1	148022
54106	1	4615
7100	1	4615
148022	1	8737
148022	1	7189
148022	1	7187
353376	1	148022
7189	1	3665
7189	1	3663
7189	1	6885
7099	1	353376
7099	1	4615
929	1	2768
929	1	10672
929	1	7099
114609	1	7189
7097	1	114609
7097	1	54472
7097	1	4615
7097	1	5879
57292	1	5781
57292	1	5777
3824	1	5781
3824	1	5777
3821	1	5781
3821	1	5777

3812	1	5781
3812	1	5777
3811	1	5781
3811	1	5777
3805	1	5781
3805	1	5777
3804	1	5781
3804	1	5777
3803	1	5781
3803	1	5777
3802	1	5781
3802	1	5777
1385	-1	2033
1385	-1	1387
4261	1	4802
4261	1	4801
4261	1	4800
4261	1	1385
4261	1	8625
4261	1	5994
4261	1	5993
5327	1	5340
3827	1	2161
3818	1	3827
3818	1	5340
2161	1	3818
2161	1	5340
5328	1	5340
5328	1	5329
5054	-1	5328
5054	-1	5327
5624	-1	2147
5624	-1	2157
5624	-1	2153
5624	1	5054
7056	1	5624
2152	1	2159
2155	1	2159
2159	1	2147
2158	1	2159
2160	1	2158
1813	1	2773
1813	1	2771
1813	1	2770
10746	1	650832
10746	1	5608
10746	1	5606
10746	1	5607
1902	1	2773
1902	1	2771
1902	1	2770
5588	1	84433
5588	1	5602
5588	1	5601
5588	1	5599

5588	1	8517
5588	1	3551
5588	1	1147
5588	1	6885
5588	1	387
5588	1	71
5588	1	648921
5588	1	646821
5588	1	646048
5588	1	60
5583	1	387
5583	1	71
5583	1	648921
5583	1	646821
5583	1	646048
5583	1	60
5581	-1	8660
5581	-1	8471
5581	-1	3667
5581	1	387
5581	1	71
5581	1	648921
5581	1	646821
5581	1	646048
5581	1	60
5580	-1	8660
5580	-1	8471
5580	-1	3667
5580	1	5894
5580	1	387
5580	1	71
5580	1	648921
5580	1	646821
5580	1	646048
5580	1	60
9863	1	5728
5728	-1	5747
5728	-1	208
5728	-1	207
5728	-1	10000
260425	1	5728
1500	1	998
1500	1	5881
1500	1	5880
1500	1	5879
81607	1	56288
25945	1	56288
10458	1	10163
10458	1	55740
10458	1	71
10458	1	648921
10458	1	646821
10458	1	646048
10458	1	60
8936	1	81873

8936	1	653888
8936	1	10552
8936	1	10109
8936	1	10095
8936	1	10094
8936	1	10093
8936	1	10092
8936	1	71
8936	1	648921
8936	1	646821
8936	1	646048
8936	1	60
10810	1	71
10810	1	648921
10810	1	646821
10810	1	646048
10810	1	60
10163	1	81873
10163	1	653888
10163	1	10552
10163	1	10109
10163	1	10095
10163	1	10094
10163	1	10093
10163	1	10092
10163	1	71
10163	1	648921
10163	1	646821
10163	1	646048
10163	1	60
4301	1	71
4301	1	648921
4301	1	646821
4301	1	646048
4301	1	60
8826	-1	1499
8826	1	71
8826	1	648921
8826	1	646821
8826	1	646048
8826	1	60
7454	1	81873
7454	1	653888
7454	1	10552
7454	1	10109
7454	1	10095
7454	1	10094
7454	1	10093
7454	1	10092
7454	1	71
7454	1	648921
7454	1	646821
7454	1	646048
7454	1	60
8976	1	81873

8976	1	653888
8976	1	10552
8976	1	10109
8976	1	10095
8976	1	10094
8976	1	10093
8976	1	10092
8976	1	71
8976	1	648921
8976	1	646821
8976	1	646048
8976	1	60
5818	1	5818
5818	1	56288
5819	1	56288
5819	1	10666
5817	1	10666
3684	1	5747
3684	1	9564
3684	1	1399
3684	1	1398
3684	1	1445
3684	1	3383
3385	1	3683
3385	1	3689
3384	1	3683
3384	1	3689
3383	1	5336
3383	1	5335
3383	1	3683
3383	1	3689
3683	1	5747
3683	1	9564
3683	1	1399
3683	1	1398
3683	1	1445
3683	1	7410
3683	1	7409
3683	1	10451
3683	1	2185
3683	1	3385
3683	1	3384
3683	1	3383
3683	1	10666
3689	1	5747
3689	1	9564
3689	1	1399
3689	1	1398
3689	1	1445
3689	1	7410
3689	1	7409
3689	1	10451
3689	1	2185
3689	1	3385
3689	1	3384

3689	1	3383
3689	1	10666
29851	1	8503
29851	1	5296
29851	1	5295
29851	1	5294
29851	1	5293
29851	1	5291
29851	1	5290
29851	1	23533
29851	1	959
23308	1	29851
649853	1	8302
649853	1	57292
649853	1	3824
649853	1	3823
649853	1	3822
649853	1	3821
649853	1	3812
649853	1	3811
649853	1	3810
649853	1	3809
649853	1	3808
649853	1	3806
649853	1	3805
649853	1	3804
649853	1	3803
649853	1	3802
649853	1	115653
649853	1	926
649853	1	925
3135	1	8302
3135	1	57292
3135	1	3824
3135	1	3823
3135	1	3822
3135	1	3821
3135	1	3812
3135	1	3811
3135	1	3810
3135	1	3809
3135	1	3808
3135	1	3806
3135	1	3805
3135	1	3804
3135	1	3803
3135	1	3802
3135	1	115653
3135	1	926
3135	1	925
3134	1	8302
3134	1	57292
3134	1	3824
3134	1	3823
3134	1	3822

3134	1	3821
3134	1	3812
3134	1	3811
3134	1	3810
3134	1	3809
3134	1	3808
3134	1	3806
3134	1	3805
3134	1	3804
3134	1	3803
3134	1	3802
3134	1	115653
3134	1	926
3134	1	925
3133	1	8302
3133	1	57292
3133	1	3824
3133	1	3823
3133	1	3822
3133	1	3821
3133	1	3812
3133	1	3811
3133	1	3810
3133	1	3809
3133	1	3808
3133	1	3806
3133	1	3805
3133	1	3804
3133	1	3803
3133	1	3802
3133	1	115653
3133	1	926
3133	1	925
3107	1	8302
3107	1	57292
3107	1	3824
3107	1	3823
3107	1	3822
3107	1	3821
3107	1	3812
3107	1	3811
3107	1	3810
3107	1	3809
3107	1	3808
3107	1	3806
3107	1	3805
3107	1	3804
3107	1	3803
3107	1	3802
3107	1	115653
3107	1	926
3107	1	925
3106	1	8302
3106	1	57292
3106	1	3824

3106	1	3823
3106	1	3822
3106	1	3821
3106	1	3812
3106	1	3811
3106	1	3810
3106	1	3809
3106	1	3808
3106	1	3806
3106	1	3805
3106	1	3804
3106	1	3803
3106	1	3802
3106	1	115653
3106	1	926
3106	1	925
3105	1	8302
3105	1	57292
3105	1	3824
3105	1	3823
3105	1	3822
3105	1	3821
3105	1	3812
3105	1	3811
3105	1	3810
3105	1	3809
3105	1	3808
3105	1	3806
3105	1	3805
3105	1	3804
3105	1	3803
3105	1	3802
3105	1	115653
3105	1	926
3105	1	925
942	1	1493
942	1	940
940	1	8503
940	1	5296
940	1	5295
940	1	5294
940	1	5293
940	1	5291
940	1	5290
940	1	23533
940	1	2885
940	1	941
941	1	1493
941	1	940
3339	1	1605
375790	1	1605
7450	1	2814
7450	1	2811
7450	1	2812
7450	1	2815

7450	1	8516
7450	1	8515
7450	1	3685
7450	1	3680
7450	1	3679
7450	1	3678
7450	1	3676
7450	1	3675
7450	1	3674
7450	1	3673
7450	1	3672
7450	1	3655
7450	1	22801
7450	1	3696
7450	1	3695
7450	1	3694
7450	1	3693
7450	1	3691
7450	1	3690
7450	1	3688
7448	1	8516
7448	1	8515
7448	1	3685
7448	1	3680
7448	1	3679
7448	1	3678
7448	1	3676
7448	1	3675
7448	1	3674
7448	1	3673
7448	1	3672
7448	1	3655
7448	1	22801
7448	1	3696
7448	1	3695
7448	1	3694
7448	1	3693
7448	1	3691
7448	1	3690
7448	1	3688
7148	1	9672
7148	1	6385
7148	1	6383
7148	1	6382
7148	1	8516
7148	1	8515
7148	1	3685
7148	1	3680
7148	1	3679
7148	1	3678
7148	1	3676
7148	1	3675
7148	1	3674
7148	1	3673
7148	1	3672

7148	1	3655
7148	1	22801
7148	1	3696
7148	1	3695
7148	1	3694
7148	1	3693
7148	1	3691
7148	1	3690
7148	1	3688
7143	1	9672
7143	1	6385
7143	1	6383
7143	1	6382
7143	1	8516
7143	1	8515
7143	1	3685
7143	1	3680
7143	1	3679
7143	1	3678
7143	1	3676
7143	1	3675
7143	1	3674
7143	1	3673
7143	1	3672
7143	1	3655
7143	1	22801
7143	1	3696
7143	1	3695
7143	1	3694
7143	1	3693
7143	1	3691
7143	1	3690
7143	1	3688
7060	-1	4052
7060	1	961
7060	1	948
7060	1	9672
7060	1	6385
7060	1	6383
7060	1	6382
7060	1	8516
7060	1	8515
7060	1	3685
7060	1	3680
7060	1	3679
7060	1	3678
7060	1	3676
7060	1	3675
7060	1	3674
7060	1	3673
7060	1	3672
7060	1	3655
7060	1	22801
7060	1	3696
7060	1	3695

7060	1	3694
7060	1	3693
7060	1	3691
7060	1	3690
7060	1	3688
7059	-1	4052
7059	1	961
7059	1	948
7059	1	9672
7059	1	6385
7059	1	6383
7059	1	6382
7059	1	8516
7059	1	8515
7059	1	3685
7059	1	3680
7059	1	3679
7059	1	3678
7059	1	3676
7059	1	3675
7059	1	3674
7059	1	3673
7059	1	3672
7059	1	3655
7059	1	22801
7059	1	3696
7059	1	3695
7059	1	3694
7059	1	3693
7059	1	3691
7059	1	3690
7059	1	3688
7058	-1	4052
7058	1	961
7058	1	948
7058	1	9672
7058	1	6385
7058	1	6383
7058	1	6382
7058	1	8516
7058	1	8515
7058	1	3685
7058	1	3680
7058	1	3679
7058	1	3678
7058	1	3676
7058	1	3675
7058	1	3674
7058	1	3673
7058	1	3672
7058	1	3655
7058	1	22801
7058	1	3696
7058	1	3695
7058	1	3694

7058	1	3693
7058	1	3691
7058	1	3690
7058	1	3688
7057	-1	4052
7057	1	961
7057	1	948
7057	1	9672
7057	1	6385
7057	1	6383
7057	1	6382
7057	1	8516
7057	1	8515
7057	1	3685
7057	1	3680
7057	1	3679
7057	1	3678
7057	1	3676
7057	1	3675
7057	1	3674
7057	1	3673
7057	1	3672
7057	1	3655
7057	1	22801
7057	1	3696
7057	1	3695
7057	1	3694
7057	1	3693
7057	1	3691
7057	1	3690
7057	1	3688
6696	1	8516
6696	1	8515
6696	1	3685
6696	1	3680
6696	1	3679
6696	1	3678
6696	1	3676
6696	1	3675
6696	1	3674
6696	1	3673
6696	1	3672
6696	1	3655
6696	1	22801
6696	1	3696
6696	1	3695
6696	1	3694
6696	1	3693
6696	1	3691
6696	1	3690
6696	1	3688
63923	1	9672
63923	1	6385
63923	1	6383
63923	1	6382

63923	1	8516
63923	1	8515
63923	1	3685
63923	1	3680
63923	1	3679
63923	1	3678
63923	1	3676
63923	1	3675
63923	1	3674
63923	1	3673
63923	1	3672
63923	1	3655
63923	1	22801
63923	1	3696
63923	1	3695
63923	1	3694
63923	1	3693
63923	1	3691
63923	1	3690
63923	1	3688
5649	1	8516
5649	1	8515
5649	1	3685
5649	1	3680
5649	1	3679
5649	1	3678
5649	1	3676
5649	1	3675
5649	1	3674
5649	1	3673
5649	1	3672
5649	1	3655
5649	1	22801
5649	1	3696
5649	1	3695
5649	1	3694
5649	1	3693
5649	1	3691
5649	1	3690
5649	1	3688
50509	1	51206
50509	1	9672
50509	1	6385
50509	1	6383
50509	1	6382
50509	1	960
50509	1	8516
50509	1	8515
50509	1	3685
50509	1	3680
50509	1	3679
50509	1	3678
50509	1	3676
50509	1	3675
50509	1	3674

50509	1	3673
50509	1	3672
50509	1	3655
50509	1	22801
50509	1	3696
50509	1	3695
50509	1	3694
50509	1	3693
50509	1	3691
50509	1	3690
50509	1	3688
3918	1	1605
3918	1	9900
3918	1	9899
3918	1	22987
3918	1	960
3918	1	8516
3918	1	8515
3918	1	3685
3918	1	3680
3918	1	3679
3918	1	3678
3918	1	3676
3918	1	3675
3918	1	3674
3918	1	3673
3918	1	3672
3918	1	3655
3918	1	22801
3918	1	3696
3918	1	3695
3918	1	3694
3918	1	3693
3918	1	3691
3918	1	3690
3918	1	3688
3915	1	1605
3915	1	9900
3915	1	9899
3915	1	22987
3915	1	960
3915	1	8516
3915	1	8515
3915	1	3685
3915	1	3680
3915	1	3679
3915	1	3678
3915	1	3676
3915	1	3675
3915	1	3674
3915	1	3673
3915	1	3672
3915	1	3655
3915	1	22801
3915	1	3696

3915	1	3695
3915	1	3694
3915	1	3693
3915	1	3691
3915	1	3690
3915	1	3688
3914	1	1605
3914	1	9900
3914	1	9899
3914	1	22987
3914	1	960
3914	1	8516
3914	1	8515
3914	1	3685
3914	1	3680
3914	1	3679
3914	1	3678
3914	1	3676
3914	1	3675
3914	1	3674
3914	1	3673
3914	1	3672
3914	1	3655
3914	1	22801
3914	1	3696
3914	1	3695
3914	1	3694
3914	1	3693
3914	1	3691
3914	1	3690
3914	1	3688
3913	1	1605
3913	1	9900
3913	1	9899
3913	1	22987
3913	1	960
3913	1	8516
3913	1	8515
3913	1	3685
3913	1	3680
3913	1	3679
3913	1	3678
3913	1	3676
3913	1	3675
3913	1	3674
3913	1	3673
3913	1	3672
3913	1	3655
3913	1	22801
3913	1	3696
3913	1	3695
3913	1	3694
3913	1	3693
3913	1	3691
3913	1	3690

3913	1	3688
3912	1	1605
3912	1	9900
3912	1	9899
3912	1	22987
3912	1	960
3912	1	8516
3912	1	8515
3912	1	3685
3912	1	3680
3912	1	3679
3912	1	3678
3912	1	3676
3912	1	3675
3912	1	3674
3912	1	3673
3912	1	3672
3912	1	3655
3912	1	22801
3912	1	3696
3912	1	3695
3912	1	3694
3912	1	3693
3912	1	3691
3912	1	3690
3912	1	3688
3911	1	1605
3911	1	9900
3911	1	9899
3911	1	22987
3911	1	960
3911	1	8516
3911	1	8515
3911	1	3685
3911	1	3680
3911	1	3679
3911	1	3678
3911	1	3676
3911	1	3675
3911	1	3674
3911	1	3673
3911	1	3672
3911	1	3655
3911	1	22801
3911	1	3696
3911	1	3695
3911	1	3694
3911	1	3693
3911	1	3691
3911	1	3690
3911	1	3688
3910	1	1605
3910	1	9900
3910	1	9899
3910	1	22987

3910	1	960
3910	1	8516
3910	1	8515
3910	1	3685
3910	1	3680
3910	1	3679
3910	1	3678
3910	1	3676
3910	1	3675
3910	1	3674
3910	1	3673
3910	1	3672
3910	1	3655
3910	1	22801
3910	1	3696
3910	1	3695
3910	1	3694
3910	1	3693
3910	1	3691
3910	1	3690
3910	1	3688
3909	1	1605
3909	1	9900
3909	1	9899
3909	1	22987
3909	1	960
3909	1	8516
3909	1	8515
3909	1	3685
3909	1	3680
3909	1	3679
3909	1	3678
3909	1	3676
3909	1	3675
3909	1	3674
3909	1	3673
3909	1	3672
3909	1	3655
3909	1	22801
3909	1	3696
3909	1	3695
3909	1	3694
3909	1	3693
3909	1	3691
3909	1	3690
3909	1	3688
3908	1	1605
3908	1	9900
3908	1	9899
3908	1	22987
3908	1	960
3908	1	8516
3908	1	8515
3908	1	3685
3908	1	3680

3908	1	3679
3908	1	3678
3908	1	3676
3908	1	3675
3908	1	3674
3908	1	3673
3908	1	3672
3908	1	3655
3908	1	22801
3908	1	3696
3908	1	3695
3908	1	3694
3908	1	3693
3908	1	3691
3908	1	3690
3908	1	3688
3381	1	8516
3381	1	8515
3381	1	3685
3381	1	3680
3381	1	3679
3381	1	3678
3381	1	3676
3381	1	3675
3381	1	3674
3381	1	3673
3381	1	3672
3381	1	3655
3381	1	22801
3381	1	3696
3381	1	3695
3381	1	3694
3381	1	3693
3381	1	3691
3381	1	3690
3381	1	3688
3371	1	9672
3371	1	6385
3371	1	6383
3371	1	6382
3371	1	8516
3371	1	8515
3371	1	3685
3371	1	3680
3371	1	3679
3371	1	3678
3371	1	3676
3371	1	3675
3371	1	3674
3371	1	3673
3371	1	3672
3371	1	3655
3371	1	22801
3371	1	3696
3371	1	3695

3371	1	3694
3371	1	3693
3371	1	3691
3371	1	3690
3371	1	3688
284217	1	1605
284217	1	9900
284217	1	9899
284217	1	22987
284217	1	960
284217	1	8516
284217	1	8515
284217	1	3685
284217	1	3680
284217	1	3679
284217	1	3678
284217	1	3676
284217	1	3675
284217	1	3674
284217	1	3673
284217	1	3672
284217	1	3655
284217	1	22801
284217	1	3696
284217	1	3695
284217	1	3694
284217	1	3693
284217	1	3691
284217	1	3690
284217	1	3688
2335	1	9672
2335	1	6385
2335	1	6383
2335	1	6382
2335	1	960
2335	1	8516
2335	1	8515
2335	1	3685
2335	1	3680
2335	1	3679
2335	1	3678
2335	1	3676
2335	1	3675
2335	1	3674
2335	1	3673
2335	1	3672
2335	1	3655
2335	1	22801
2335	1	3696
2335	1	3695
2335	1	3694
2335	1	3693
2335	1	3691
2335	1	3690
2335	1	3688

22798	1	1605
22798	1	9900
22798	1	9899
22798	1	22987
22798	1	960
22798	1	8516
22798	1	8515
22798	1	3685
22798	1	3680
22798	1	3679
22798	1	3678
22798	1	3676
22798	1	3675
22798	1	3674
22798	1	3673
22798	1	3672
22798	1	3655
22798	1	22801
22798	1	3696
22798	1	3695
22798	1	3694
22798	1	3693
22798	1	3691
22798	1	3690
22798	1	3688
131873	1	51206
131873	1	9672
131873	1	6385
131873	1	6383
131873	1	6382
131873	1	960
131873	1	8516
131873	1	8515
131873	1	3685
131873	1	3680
131873	1	3679
131873	1	3678
131873	1	3676
131873	1	3675
131873	1	3674
131873	1	3673
131873	1	3672
131873	1	3655
131873	1	22801
131873	1	3696
131873	1	3695
131873	1	3694
131873	1	3693
131873	1	3691
131873	1	3690
131873	1	3688
1311	-1	4052
1311	1	961
1311	1	948
1311	1	9672

1311	1	6385
1311	1	6383
1311	1	6382
1311	1	8516
1311	1	8515
1311	1	3685
1311	1	3680
1311	1	3679
1311	1	3678
1311	1	3676
1311	1	3675
1311	1	3674
1311	1	3673
1311	1	3672
1311	1	3655
1311	1	22801
1311	1	3696
1311	1	3695
1311	1	3694
1311	1	3693
1311	1	3691
1311	1	3690
1311	1	3688
1302	1	51206
1302	1	9672
1302	1	6385
1302	1	6383
1302	1	6382
1302	1	960
1302	1	8516
1302	1	8515
1302	1	3685
1302	1	3680
1302	1	3679
1302	1	3678
1302	1	3676
1302	1	3675
1302	1	3674
1302	1	3673
1302	1	3672
1302	1	3655
1302	1	22801
1302	1	3696
1302	1	3695
1302	1	3694
1302	1	3693
1302	1	3691
1302	1	3690
1302	1	3688
1301	1	51206
1301	1	9672
1301	1	6385
1301	1	6383
1301	1	6382
1301	1	960

1301	1	8516
1301	1	8515
1301	1	3685
1301	1	3680
1301	1	3679
1301	1	3678
1301	1	3676
1301	1	3675
1301	1	3674
1301	1	3673
1301	1	3672
1301	1	3655
1301	1	22801
1301	1	3696
1301	1	3695
1301	1	3694
1301	1	3693
1301	1	3691
1301	1	3690
1301	1	3688
1293	1	51206
1293	1	9672
1293	1	6385
1293	1	6383
1293	1	6382
1293	1	960
1293	1	8516
1293	1	8515
1293	1	3685
1293	1	3680
1293	1	3679
1293	1	3678
1293	1	3676
1293	1	3675
1293	1	3674
1293	1	3673
1293	1	3672
1293	1	3655
1293	1	22801
1293	1	3696
1293	1	3695
1293	1	3694
1293	1	3693
1293	1	3691
1293	1	3690
1293	1	3688
1292	1	51206
1292	1	9672
1292	1	6385
1292	1	6383
1292	1	6382
1292	1	960
1292	1	8516
1292	1	8515
1292	1	3685

1292	1	3680
1292	1	3679
1292	1	3678
1292	1	3676
1292	1	3675
1292	1	3674
1292	1	3673
1292	1	3672
1292	1	3655
1292	1	22801
1292	1	3696
1292	1	3695
1292	1	3694
1292	1	3693
1292	1	3691
1292	1	3690
1292	1	3688
1291	1	51206
1291	1	9672
1291	1	6385
1291	1	6383
1291	1	6382
1291	1	960
1291	1	8516
1291	1	8515
1291	1	3685
1291	1	3680
1291	1	3679
1291	1	3678
1291	1	3676
1291	1	3675
1291	1	3674
1291	1	3673
1291	1	3672
1291	1	3655
1291	1	22801
1291	1	3696
1291	1	3695
1291	1	3694
1291	1	3693
1291	1	3691
1291	1	3690
1291	1	3688
1290	1	51206
1290	1	9672
1290	1	6385
1290	1	6383
1290	1	6382
1290	1	960
1290	1	8516
1290	1	8515
1290	1	3685
1290	1	3680
1290	1	3679
1290	1	3678

1290	1	3676
1290	1	3675
1290	1	3674
1290	1	3673
1290	1	3672
1290	1	3655
1290	1	22801
1290	1	3696
1290	1	3695
1290	1	3694
1290	1	3693
1290	1	3691
1290	1	3690
1290	1	3688
1289	1	51206
1289	1	9672
1289	1	6385
1289	1	6383
1289	1	6382
1289	1	960
1289	1	8516
1289	1	8515
1289	1	3685
1289	1	3680
1289	1	3679
1289	1	3678
1289	1	3676
1289	1	3675
1289	1	3674
1289	1	3673
1289	1	3672
1289	1	3655
1289	1	22801
1289	1	3696
1289	1	3695
1289	1	3694
1289	1	3693
1289	1	3691
1289	1	3690
1289	1	3688
1288	1	51206
1288	1	9672
1288	1	6385
1288	1	6383
1288	1	6382
1288	1	960
1288	1	8516
1288	1	8515
1288	1	3685
1288	1	3680
1288	1	3679
1288	1	3678
1288	1	3676
1288	1	3675
1288	1	3674

1288	1	3673
1288	1	3672
1288	1	3655
1288	1	22801
1288	1	3696
1288	1	3695
1288	1	3694
1288	1	3693
1288	1	3691
1288	1	3690
1288	1	3688
1286	1	51206
1286	1	9672
1286	1	6385
1286	1	6383
1286	1	6382
1286	1	960
1286	1	8516
1286	1	8515
1286	1	3685
1286	1	3680
1286	1	3679
1286	1	3678
1286	1	3676
1286	1	3675
1286	1	3674
1286	1	3673
1286	1	3672
1286	1	3655
1286	1	22801
1286	1	3696
1286	1	3695
1286	1	3694
1286	1	3693
1286	1	3691
1286	1	3690
1286	1	3688
1284	1	51206
1284	1	9672
1284	1	6385
1284	1	6383
1284	1	6382
1284	1	960
1284	1	8516
1284	1	8515
1284	1	3685
1284	1	3680
1284	1	3679
1284	1	3678
1284	1	3676
1284	1	3675
1284	1	3674
1284	1	3673
1284	1	3672
1284	1	3655

1284	1	22801
1284	1	3696
1284	1	3695
1284	1	3694
1284	1	3693
1284	1	3691
1284	1	3690
1284	1	3688
1282	1	51206
1282	1	9672
1282	1	6385
1282	1	6383
1282	1	6382
1282	1	960
1282	1	8516
1282	1	8515
1282	1	3685
1282	1	3680
1282	1	3679
1282	1	3678
1282	1	3676
1282	1	3675
1282	1	3674
1282	1	3673
1282	1	3672
1282	1	3655
1282	1	22801
1282	1	3696
1282	1	3695
1282	1	3694
1282	1	3693
1282	1	3691
1282	1	3690
1282	1	3688
1281	1	51206
1281	1	9672
1281	1	6385
1281	1	6383
1281	1	6382
1281	1	960
1281	1	8516
1281	1	8515
1281	1	3685
1281	1	3680
1281	1	3679
1281	1	3678
1281	1	3676
1281	1	3675
1281	1	3674
1281	1	3673
1281	1	3672
1281	1	3655
1281	1	22801
1281	1	3696
1281	1	3695

1281	1	3694
1281	1	3693
1281	1	3691
1281	1	3690
1281	1	3688
1280	1	51206
1280	1	9672
1280	1	6385
1280	1	6383
1280	1	6382
1280	1	960
1280	1	8516
1280	1	8515
1280	1	3685
1280	1	3680
1280	1	3679
1280	1	3678
1280	1	3676
1280	1	3675
1280	1	3674
1280	1	3673
1280	1	3672
1280	1	3655
1280	1	22801
1280	1	3696
1280	1	3695
1280	1	3694
1280	1	3693
1280	1	3691
1280	1	3690
1280	1	3688
1278	1	51206
1278	1	9672
1278	1	6385
1278	1	6383
1278	1	6382
1278	1	960
1278	1	8516
1278	1	8515
1278	1	3685
1278	1	3680
1278	1	3679
1278	1	3678
1278	1	3676
1278	1	3675
1278	1	3674
1278	1	3673
1278	1	3672
1278	1	3655
1278	1	22801
1278	1	3696
1278	1	3695
1278	1	3694
1278	1	3693
1278	1	3691

1278	1	3690
1278	1	3688
1277	1	51206
1277	1	9672
1277	1	6385
1277	1	6383
1277	1	6382
1277	1	960
1277	1	8516
1277	1	8515
1277	1	3685
1277	1	3680
1277	1	3679
1277	1	3678
1277	1	3676
1277	1	3675
1277	1	3674
1277	1	3673
1277	1	3672
1277	1	3655
1277	1	22801
1277	1	3696
1277	1	3695
1277	1	3694
1277	1	3693
1277	1	3691
1277	1	3690
1277	1	3688
1101	1	8516
1101	1	8515
1101	1	3685
1101	1	3680
1101	1	3679
1101	1	3678
1101	1	3676
1101	1	3675
1101	1	3674
1101	1	3673
1101	1	3672
1101	1	3655
1101	1	22801
1101	1	3696
1101	1	3695
1101	1	3694
1101	1	3693
1101	1	3691
1101	1	3690
1101	1	3688
8516	1	5747
8516	1	9564
8516	1	1399
8516	1	1398
8516	1	1445
8515	1	5747
8515	1	9564

8515	1	1399
8515	1	1398
8515	1	1445
3685	1	5747
3685	1	9564
3685	1	1399
3685	1	1398
3685	1	1445
3680	1	5747
3680	1	9564
3680	1	1399
3680	1	1398
3680	1	1445
3680	1	7412
3679	1	5747
3679	1	9564
3679	1	1399
3679	1	1398
3679	1	1445
3678	1	5747
3678	1	9564
3678	1	1399
3678	1	1398
3678	1	1445
3676	1	5747
3676	1	9564
3676	1	1399
3676	1	1398
3676	1	1445
3676	1	7412
3675	1	5747
3675	1	9564
3675	1	1399
3675	1	1398
3675	1	1445
3674	1	5747
3674	1	9564
3674	1	1399
3674	1	1398
3674	1	1445
3673	1	5747
3673	1	9564
3673	1	1399
3673	1	1398
3673	1	1445
3672	1	5747
3672	1	9564
3672	1	1399
3672	1	1398
3672	1	1445
3655	1	5747
3655	1	9564
3655	1	1399
3655	1	1398
3655	1	1445

22801	1	5747
22801	1	9564
22801	1	1399
22801	1	1398
22801	1	1445
10319	1	1605
10319	1	9900
10319	1	9899
10319	1	22987
10319	1	960
10319	1	8516
10319	1	8515
10319	1	3685
10319	1	3680
10319	1	3679
10319	1	3678
10319	1	3676
10319	1	3675
10319	1	3674
10319	1	3673
10319	1	3672
10319	1	3655
10319	1	22801
10319	1	3696
10319	1	3695
10319	1	3694
10319	1	3693
10319	1	3691
10319	1	3690
10319	1	3688
80310	1	3645
80310	1	2263
80310	1	2260
80310	1	5159
80310	1	5156
80310	1	4233
80310	1	3791
80310	1	3480
80310	1	2324
80310	1	2321
80310	1	2064
80310	1	1956
5228	1	5159
5228	1	5156
5228	1	4233
5228	1	3791
5228	1	3480
5228	1	2324
5228	1	2321
5228	1	2064
5228	1	1956
3479	1	3645
3479	1	2263
3479	1	2260
3479	1	5159

3479	1	5156
3479	1	4233
3479	1	3791
3479	1	3480
3479	1	2324
3479	1	2321
3479	1	2064
3479	1	1956
3480	-1	1499
3480	1	4893
3480	1	3845
3480	1	3265
3480	1	2885
3480	1	5336
3480	1	5335
3480	1	2781
3480	1	2778
3480	1	2775
3480	1	2773
3480	1	2771
3480	1	2770
3480	1	2768
3480	1	10672
3480	1	6464
3480	1	53358
3480	1	399694
3480	1	25759
3480	1	8503
3480	1	5296
3480	1	5295
3480	1	5294
3480	1	5293
3480	1	5291
3480	1	5290
3480	1	23533
3480	1	5747
2889	1	23433
2889	1	5908
2889	1	5906
1793	1	5881
1793	1	5880
1793	1	5879
9564	1	1793
9564	1	1399
9564	1	1398
859	1	2534
858	1	2534
857	1	2534
7410	1	998
7410	1	387
7410	1	5881
7410	1	5880
7410	1	5879
7409	1	998
7409	1	387

7409	1	5881
7409	1	5880
7409	1	5879
10451	1	998
10451	1	387
10451	1	5881
10451	1	5880
10451	1	5879
3696	1	9564
3696	1	1399
3696	1	1398
3696	1	1445
3696	1	5747
3696	1	6714
3695	1	9564
3695	1	1399
3695	1	1398
3695	1	1445
3695	1	5747
3695	1	6714
3694	1	9564
3694	1	1399
3694	1	1398
3694	1	1445
3694	1	5747
3694	1	6714
3693	1	9564
3693	1	1399
3693	1	1398
3693	1	1445
3693	1	5747
3693	1	6714
3691	1	9564
3691	1	1399
3691	1	1398
3691	1	1445
3691	1	5747
3691	1	6714
3690	1	9564
3690	1	1399
3690	1	1398
3690	1	1445
3690	1	5747
3690	1	6714
93408	1	71
93408	1	648921
93408	1	646821
93408	1	646048
93408	1	60
58498	1	71
58498	1	648921
58498	1	646821
58498	1	646048
58498	1	60
4636	1	71

4636	1	648921
4636	1	646821
4636	1	646048
4636	1	60
4633	1	71
4633	1	648921
4633	1	646821
4633	1	646048
4633	1	60
29895	1	71
29895	1	648921
29895	1	646821
29895	1	646048
29895	1	60
10627	1	71
10627	1	648921
10627	1	646821
10627	1	646048
10627	1	60
10398	1	71
10398	1	648921
10398	1	646821
10398	1	646048
10398	1	60
103910	1	71
103910	1	648921
103910	1	646821
103910	1	646048
103910	1	60
23396	-1	85477
23396	-1	2934
1729	1	5217
1729	1	5216
1729	1	375189
1729	1	345456
9855	1	998
9855	1	387
5582	1	72
5582	1	70
5582	1	59
5582	1	58
5582	1	5336
5582	1	5335
5582	1	5747
5582	1	8877
5582	1	56848
5582	1	673
5582	1	369
5582	1	387
5582	1	71
5582	1	648921
5582	1	646821
5582	1	646048
5582	1	60
5579	1	72

5579	1	70
5579	1	59
5579	1	58
5579	1	5336
5579	1	5335
5579	1	7299
5579	1	5747
5579	1	8877
5579	1	56848
5579	1	673
5579	1	369
5579	1	387
5579	1	71
5579	1	648921
5579	1	646821
5579	1	646048
5579	1	60
5578	1	72
5578	1	70
5578	1	59
5578	1	58
5578	1	5336
5578	1	5335
5578	1	5747
5578	1	8877
5578	1	56848
5578	1	673
5578	1	369
5578	1	387
5578	1	71
5578	1	648921
5578	1	646821
5578	1	646048
5578	1	60
63928	-1	5502
63928	1	572
5535	-1	5502
5535	1	572
5534	-1	5502
5534	1	572
5533	-1	5502
5533	1	572
5532	-1	5502
5532	1	572
5530	-1	5502
5530	1	572
11261	-1	5502
11261	1	572
1020	1	1808
64218	1	5365
64218	1	5364
64218	1	23654
57715	1	5365
57715	1	5364
57715	1	23654

54910	1	5365
54910	1	5364
54910	1	23654
10509	1	5365
10509	1	5364
10509	1	23654
10507	1	5365
10507	1	5364
10507	1	23654
10505	1	5365
10505	1	5364
10505	1	23654
3688	1	9564
3688	1	1399
3688	1	1398
3688	1	1445
3688	1	7412
3688	1	6714
8482	1	10154
8482	1	3688
5365	-1	5881
5365	-1	5880
5365	-1	5879
5365	1	27289
5365	1	23365
5364	-1	5881
5364	-1	5880
5364	-1	5879
5364	1	27289
5364	1	23365
23365	1	387
23654	-1	5881
23654	-1	5880
23654	-1	5879
23654	1	27289
23654	1	23365
27289	1	23365
55558	1	27289
55558	1	5881
55558	1	5880
55558	1	5879
5362	1	27289
5362	1	5881
5362	1	5880
5362	1	5879
5361	1	27289
5361	1	5881
5361	1	5880
5361	1	5879
9901	-1	998
57522	-1	998
23380	-1	998
9353	1	6091
9353	1	6092
6091	1	9901

6091	1	57522
6091	1	23380
6092	1	9901
6092	1	57522
6092	1	23380
6585	1	6091
6585	1	6092
2773	-1	196883
2773	-1	115
2773	-1	114
2773	-1	113
2773	-1	112
2773	-1	111
2773	-1	109
2773	-1	108
2773	-1	107
2773	1	5332
2773	1	5331
2773	1	5330
2773	1	23236
2773	1	8681
2773	1	84647
2773	1	8399
2773	1	8398
2773	1	81579
2773	1	64600
2773	1	5322
2773	1	5321
2773	1	5320
2773	1	5319
2773	1	50487
2773	1	30814
2773	1	26279
2773	1	1E+08
2773	1	8503
2773	1	5296
2773	1	5295
2773	1	5294
2773	1	5293
2773	1	5291
2773	1	5290
2773	1	23533
2773	1	4893
2773	1	3845
2773	1	3265
2771	-1	196883
2771	-1	115
2771	-1	114
2771	-1	113
2771	-1	112
2771	-1	111
2771	-1	109
2771	-1	108
2771	-1	107
2771	1	5332

2771	1	5331
2771	1	5330
2771	1	23236
2771	1	8681
2771	1	84647
2771	1	8399
2771	1	8398
2771	1	81579
2771	1	64600
2771	1	5322
2771	1	5321
2771	1	5320
2771	1	5319
2771	1	50487
2771	1	30814
2771	1	26279
2771	1	1E+08
2771	1	8503
2771	1	5296
2771	1	5295
2771	1	5294
2771	1	5293
2771	1	5291
2771	1	5290
2771	1	23533
2771	1	4893
2771	1	3845
2771	1	3265
2770	-1	196883
2770	-1	115
2770	-1	114
2770	-1	113
2770	-1	112
2770	-1	111
2770	-1	109
2770	-1	108
2770	-1	107
2770	1	5332
2770	1	5331
2770	1	5330
2770	1	23236
2770	1	8681
2770	1	84647
2770	1	8399
2770	1	8398
2770	1	81579
2770	1	64600
2770	1	5322
2770	1	5321
2770	1	5320
2770	1	5319
2770	1	50487
2770	1	30814
2770	1	26279
2770	1	1E+08

2770	1	8503
2770	1	5296
2770	1	5295
2770	1	5294
2770	1	5293
2770	1	5291
2770	1	5290
2770	1	23533
2770	1	4893
2770	1	3845
2770	1	3265
25791	-1	998
25791	-1	5881
25791	-1	5880
25791	-1	5879
25791	1	387
2051	1	5921
2051	1	25
2051	1	1949
2051	1	1948
2051	1	1947
2050	1	5921
2050	1	25
2050	1	1949
2050	1	1948
2050	1	1947
2049	1	5921
2049	1	25
2049	1	1949
2049	1	1948
2049	1	1947
2048	1	5921
2048	1	25
2048	1	1949
2048	1	1948
2048	1	1947
2047	1	5921
2047	1	25
2047	1	1949
2047	1	1948
2047	1	1947
285220	1	5921
285220	1	25791
285220	1	25
2046	1	5921
2046	1	25791
2046	1	25
2045	1	5921
2045	1	25791
2045	1	25
2044	1	5921
2044	1	25791
2044	1	25
2043	1	5921
2043	1	25791

2043	1	25
2042	1	5921
2042	1	25791
2042	1	25
2041	1	5921
2041	1	25791
2041	1	25
1969	1	5921
1969	1	25791
1969	1	25
5747	1	6464
5747	1	53358
5747	1	399694
5747	1	25759
5747	1	1793
5747	1	8503
5747	1	5296
5747	1	5295
5747	1	5294
5747	1	5293
5747	1	5291
5747	1	5290
5747	1	23533
1949	1	8440
1948	1	8440
1947	1	8440
2534	-1	1500
2534	-1	1499
2534	1	9846
2534	1	51744
2534	1	919
2534	1	2207
2534	1	7305
2534	1	6464
2534	1	53358
2534	1	399694
2534	1	25759
2534	1	55558
2534	1	5362
2534	1	5361
2534	1	1020
1946	1	285220
1946	1	2046
1946	1	2045
1946	1	2044
1946	1	2043
1946	1	2042
1946	1	2041
1946	1	1969
1946	1	2534
1946	1	5921
1945	1	285220
1945	1	2046
1945	1	2045
1945	1	2044

1945	1	2043
1945	1	2042
1945	1	2041
1945	1	1969
1945	1	2534
1945	1	5921
1944	1	285220
1944	1	2046
1944	1	2045
1944	1	2044
1944	1	2043
1944	1	2042
1944	1	2041
1944	1	1969
1944	1	2534
1944	1	5921
1943	1	285220
1943	1	2046
1943	1	2045
1943	1	2044
1943	1	2043
1943	1	2042
1943	1	2041
1943	1	1969
1943	1	2534
1943	1	5921
5921	-1	6237
5921	-1	4893
5921	-1	3845
5921	-1	3265
5921	-1	22808
5921	-1	22800
1942	1	285220
1942	1	2046
1942	1	2045
1942	1	2044
1942	1	2043
1942	1	2042
1942	1	2041
1942	1	1969
1942	1	2534
1942	1	5921
1630	-1	387
1630	1	4690
9423	1	90249
9423	1	8633
9423	1	219699
9423	1	137970
9423	1	1630
5519	-1	83700
5519	-1	58494
5519	-1	50848
5519	-1	150084
5519	-1	5590
5519	-1	5584

5519	-1	5582
5519	-1	5579
5519	-1	5578
5519	-1	5595
5519	-1	5594
5519	-1	5605
5519	-1	5604
5518	-1	83700
5518	-1	58494
5518	-1	50848
5518	-1	150084
5518	-1	5590
5518	-1	5584
5518	-1	5582
5518	-1	5579
5518	-1	5578
5518	-1	5595
5518	-1	5594
5518	-1	5605
5518	-1	5604
5516	-1	83700
5516	-1	58494
5516	-1	50848
5516	-1	150084
5516	-1	5590
5516	-1	5584
5516	-1	5582
5516	-1	5579
5516	-1	5578
5516	-1	5595
5516	-1	5594
5516	-1	5605
5516	-1	5604
5515	-1	83700
5515	-1	58494
5515	-1	50848
5515	-1	150084
5515	-1	5590
5515	-1	5584
5515	-1	5582
5515	-1	5579
5515	-1	5578
5515	-1	5595
5515	-1	5594
5515	-1	5605
5515	-1	5604
4088	1	2033
4088	1	1387
4088	1	6667
4088	1	5934
4088	1	5933
4088	1	1875
4088	1	1874
4088	1	7027
4088	1	4089

4087	1	2033
4087	1	1387
4087	1	6667
4087	1	5934
4087	1	5933
4087	1	1875
4087	1	1874
4087	1	7027
4087	1	4089
4093	1	4089
4090	1	4089
4086	1	4089
4838	1	93
4838	1	92
656	1	659
656	1	269
654	1	659
654	1	269
653	1	659
653	1	269
652	1	659
652	1	269
392255	1	659
392255	1	269
353500	1	659
353500	1	269
151449	1	659
151449	1	269
9794	1	3516
9794	1	11317
84441	1	3516
84441	1	11317
55534	1	3516
55534	1	11317
8850	1	3516
8850	1	11317
2648	1	3516
2648	1	11317
5986	1	4855
5986	1	4854
5986	1	4853
5986	1	4851
4242	1	4855
4242	1	4854
4242	1	4853
4242	1	4851
3955	1	4855
3955	1	4854
3955	1	4853
3955	1	4851
55851	1	4855
55851	1	4854
55851	1	4853
55851	1	4851
5664	-1	22926

5664	-1	2081
5664	-1	9451
5664	1	4855
5664	1	4854
5664	1	4853
5664	1	4851
51107	1	4855
51107	1	4854
51107	1	4853
51107	1	4851
23385	1	4855
23385	1	4854
23385	1	4853
23385	1	4851
6868	1	1839
6868	1	4855
6868	1	4854
6868	1	4853
6868	1	4851
54567	1	4855
54567	1	4854
54567	1	4853
54567	1	4851
28514	1	4855
28514	1	4854
28514	1	4853
28514	1	4851
4855	1	3516
4855	1	11317
4855	1	23220
4855	1	196403
4855	1	1840
4855	1	151636
4855	1	113878
4854	1	3516
4854	1	11317
4854	1	23220
4854	1	196403
4854	1	1840
4854	1	151636
4854	1	113878
4853	1	3516
4853	1	11317
4853	1	23220
4853	1	196403
4853	1	1840
4853	1	151636
4853	1	113878
4851	1	3516
4851	1	11317
4851	1	23220
4851	1	196403
4851	1	1840
4851	1	151636
4851	1	113878

10683	1	4855
10683	1	4854
10683	1	4853
10683	1	4851
5602	-1	8660
5602	-1	8471
5602	-1	3667
5602	-1	4776
5602	-1	4773
5601	-1	8660
5601	-1	8471
5601	-1	3667
5601	-1	4776
5601	-1	4773
5601	1	2353
5599	-1	8660
5599	-1	8471
5599	-1	3667
5599	-1	4776
5599	-1	4773
9475	-1	5501
9475	-1	5500
9475	-1	5499
9475	-1	4659
6093	-1	5501
6093	-1	5500
6093	-1	5499
6093	-1	4659
387	-1	5881
387	-1	5880
387	-1	5879
387	1	1730
387	1	71
387	1	648921
387	1	646821
387	1	646048
387	1	60
387	1	23396
387	1	1729
387	1	9475
387	1	6093
85409	1	1857
85409	1	1856
85409	1	1855
1857	-1	4855
1857	-1	4854
1857	-1	4853
1857	-1	4851
1857	-1	2932
1856	-1	4855
1856	-1	4854
1856	-1	4853
1856	-1	4851
1856	-1	2932
1855	-1	4855

1855	-1	4854
1855	-1	4853
1855	-1	4851
1855	-1	2932
85407	1	1857
85407	1	1856
85407	1	1855
4089	1	2033
4089	1	1387
4089	1	6667
4089	1	5934
4089	1	5933
4089	1	1875
4089	1	1874
4089	1	7027
4089	1	83439
4089	1	6934
4089	1	6932
4089	1	51176
1499	1	1499
1499	-1	2033
1499	-1	1387
1499	1	83439
1499	1	6934
1499	1	6932
1499	1	51176
5663	-1	22926
5663	-1	2081
5663	-1	9451
5663	1	4855
5663	1	4854
5663	1	4853
5663	1	4851
5663	1	1499
8326	1	2776
8326	1	2775
8325	1	2776
8325	1	2775
8324	1	2776
8324	1	2775
8323	1	2776
8323	1	2775
8322	1	2776
8322	1	2775
8321	1	2776
8321	1	2775
7976	1	2776
7976	1	2775
7855	1	2776
7855	1	2775
2535	1	2776
2535	1	2775
11211	1	2776
11211	1	2775
89780	1	8326

89780	1	8325
89780	1	8324
89780	1	8323
89780	1	8322
89780	1	8321
89780	1	7976
89780	1	7855
89780	1	2535
89780	1	11211
81029	1	8326
81029	1	8325
81029	1	8324
81029	1	8323
81029	1	8322
81029	1	8321
81029	1	7976
81029	1	7855
81029	1	2535
81029	1	11211
80326	1	8326
80326	1	8325
80326	1	8324
80326	1	8323
80326	1	8322
80326	1	8321
80326	1	7976
80326	1	7855
80326	1	2535
80326	1	11211
7484	1	8326
7484	1	8325
7484	1	8324
7484	1	8323
7484	1	8322
7484	1	8321
7484	1	7976
7484	1	7855
7484	1	2535
7484	1	11211
7483	1	8326
7483	1	8325
7483	1	8324
7483	1	8323
7483	1	8322
7483	1	8321
7483	1	7976
7483	1	7855
7483	1	2535
7483	1	11211
7482	1	8326
7482	1	8325
7482	1	8324
7482	1	8323
7482	1	8322
7482	1	8321

7482	1	7976
7482	1	7855
7482	1	2535
7482	1	11211
7481	1	8326
7481	1	8325
7481	1	8324
7481	1	8323
7481	1	8322
7481	1	8321
7481	1	7976
7481	1	7855
7481	1	2535
7481	1	11211
7480	1	8326
7480	1	8325
7480	1	8324
7480	1	8323
7480	1	8322
7480	1	8321
7480	1	7976
7480	1	7855
7480	1	2535
7480	1	11211
7479	1	8326
7479	1	8325
7479	1	8324
7479	1	8323
7479	1	8322
7479	1	8321
7479	1	7976
7479	1	7855
7479	1	2535
7479	1	11211
7478	1	8326
7478	1	8325
7478	1	8324
7478	1	8323
7478	1	8322
7478	1	8321
7478	1	7976
7478	1	7855
7478	1	2535
7478	1	11211
7477	1	8326
7477	1	8325
7477	1	8324
7477	1	8323
7477	1	8322
7477	1	8321
7477	1	7976
7477	1	7855
7477	1	2535
7477	1	11211
7476	1	8326

7476	1	8325
7476	1	8324
7476	1	8323
7476	1	8322
7476	1	8321
7476	1	7976
7476	1	7855
7476	1	2535
7476	1	11211
7475	1	8326
7475	1	8325
7475	1	8324
7475	1	8323
7475	1	8322
7475	1	8321
7475	1	7976
7475	1	7855
7475	1	2535
7475	1	11211
7474	1	8326
7474	1	8325
7474	1	8324
7474	1	8323
7474	1	8322
7474	1	8321
7474	1	7976
7474	1	7855
7474	1	2535
7474	1	11211
7473	1	8326
7473	1	8325
7473	1	8324
7473	1	8323
7473	1	8322
7473	1	8321
7473	1	7976
7473	1	7855
7473	1	2535
7473	1	11211
7472	1	8326
7472	1	8325
7472	1	8324
7472	1	8323
7472	1	8322
7472	1	8321
7472	1	7976
7472	1	7855
7472	1	2535
7472	1	11211
7471	1	8326
7471	1	8325
7471	1	8324
7471	1	8323
7471	1	8322
7471	1	8321

7471	1	7976
7471	1	7855
7471	1	2535
7471	1	11211
54361	1	8326
54361	1	8325
54361	1	8324
54361	1	8323
54361	1	8322
54361	1	8321
54361	1	7976
54361	1	7855
54361	1	2535
54361	1	11211
51384	1	8326
51384	1	8325
51384	1	8324
51384	1	8323
51384	1	8322
51384	1	8321
51384	1	7976
51384	1	7855
51384	1	2535
51384	1	11211
64840	1	89780
64840	1	81029
64840	1	80326
64840	1	7484
64840	1	7483
64840	1	7482
64840	1	7481
64840	1	7480
64840	1	7479
64840	1	7478
64840	1	7477
64840	1	7476
64840	1	7475
64840	1	7474
64840	1	7473
64840	1	7472
64840	1	7471
64840	1	54361
64840	1	51384
9020	1	8517
9020	1	3551
9020	1	1147
840	1	1676
843	1	836
4615	1	7189
4615	1	51135
4615	1	3654
4615	1	8772
51135	1	3654
51135	1	7189
3654	1	3665

3654	1	7189
8737	1	6885
8737	1	9020
8717	1	7186
8772	1	841
8772	1	843
2475	-1	9706
2475	-1	8408
2475	-1	25989
2475	-1	1978
2475	-1	8660
2475	-1	8471
2475	-1	3667
2475	1	6199
2475	1	6198
6009	-1	673
6009	1	2475
841	1	637
841	1	840
841	1	836
317	1	842
842	1	840
842	1	836
54205	1	842
4193	-1	7157
4193	-1	5925
4194	-1	7157
4194	1	4193
731751	1	7157
5591	1	7157
2033	1	3516
2033	1	11317
2033	1	7157
7157	1	4193
1387	1	3516
1387	1	11317
1387	1	7157
5111	-1	896
5111	-1	894
5111	-1	595
5111	-1	1021
5111	-1	1019
1442	1	2690
7252	1	7253
3972	1	3973
2488	1	2492
1081	1	7253
1081	1	3973
1081	1	2492
5646	1	9002
5646	1	56288
5646	1	2151
5646	1	2150
5646	1	2149
5645	1	9002

5645	1	56288
5645	1	2151
5645	1	2150
5645	1	2149
5644	1	9002
5644	1	56288
5644	1	2151
5644	1	2150
5644	1	2149
5340	1	9002
5340	1	56288
5340	1	2151
5340	1	2150
5340	1	2149
3001	1	9002
3001	1	56288
3001	1	2151
3001	1	2150
3001	1	2149
2147	1	2160
2147	1	2158
2147	1	7056
2147	1	2165
2147	1	2162
2147	1	2266
2147	1	2244
2147	1	2243
2147	1	9002
2147	1	56288
2147	1	2151
2147	1	2150
2147	1	2149
56288	1	83700
56288	1	58494
56288	1	50848
56288	1	150084
1511	1	9002
1511	1	56288
1511	1	2151
1511	1	2150
1511	1	2149
3707	1	810
3707	1	808
3707	1	805
3707	1	801
3707	1	51806
3707	1	163688
3706	1	810
3706	1	808
3706	1	805
3706	1	801
3706	1	51806
3706	1	163688
3556	1	4615
3556	1	51135

3556	1	3656
3556	1	3654
3556	1	11213
3556	1	8717
3556	1	8772
8200	1	659
8200	1	269
8200	1	93
8200	1	92
8200	1	657
655	1	659
655	1	269
655	1	93
655	1	92
655	1	658
650	1	269
650	1	657
650	1	658
650	1	659
650	1	94
650	1	90
268	1	659
268	1	269
268	1	94
268	1	90
83729	-1	4838
83729	-1	7043
83729	-1	7042
83729	-1	7040
83729	1	91
83729	1	93
83729	1	92
83729	1	94
83729	1	90
3626	-1	4838
3626	-1	7043
3626	-1	7042
3626	-1	7040
3626	1	91
3626	1	93
3626	1	92
3626	1	94
3626	1	90
3625	-1	4838
3625	-1	7043
3625	-1	7042
3625	-1	7040
3625	1	91
3625	1	93
3625	1	92
3625	1	94
3625	1	90
3624	-1	4838
3624	-1	7043
3624	-1	7042

3624	-1	7040
3624	1	91
3624	1	93
3624	1	92
3624	1	94
3624	1	90
959	1	958
8740	1	8771
8740	1	8764
8740	1	4055
4050	1	4055
4049	1	5721
4049	1	5720
4049	1	10197
4049	1	8764
4049	1	4055
4049	1	7132
4049	1	7133
7133	1	1616
7133	1	7186
8600	1	8792
8600	1	4982
8793	1	8772
8794	1	8772
8795	1	8772
8797	1	8772
8743	1	4982
8743	1	8793
8743	1	8794
8743	1	8795
8743	1	8797
282618	1	163702
282618	1	3588
282617	1	163702
282617	1	3588
282616	1	163702
282616	1	3588
50616	1	116379
50616	1	58985
50616	1	3588
11009	1	58985
11009	1	53832
50604	1	58985
50604	1	53832
29949	1	53832
3586	1	3587
3586	1	3588
3458	1	3455
3458	1	3454
3458	1	3459
3458	1	3460
338376	1	3454
338376	1	3455
56832	1	3454
56832	1	3455

3467	1	3454
3467	1	3455
3456	1	3460
3456	1	3459
3456	1	3454
3456	1	3455
3452	1	3460
3452	1	3459
3452	1	5721
3452	1	5720
3452	1	10197
3452	1	3454
3452	1	3455
3451	1	3460
3451	1	3459
3451	1	5721
3451	1	5720
3451	1	10197
3451	1	3454
3451	1	3455
3449	1	3460
3449	1	3459
3449	1	5721
3449	1	5720
3449	1	10197
3449	1	3454
3449	1	3455
3448	1	3460
3448	1	3459
3448	1	5721
3448	1	5720
3448	1	10197
3448	1	3454
3448	1	3455
3447	1	3460
3447	1	3459
3447	1	5721
3447	1	5720
3447	1	10197
3447	1	3454
3447	1	3455
3446	1	3460
3446	1	3459
3446	1	5721
3446	1	5720
3446	1	10197
3446	1	3454
3446	1	3455
3445	1	3460
3445	1	3459
3445	1	5721
3445	1	5720
3445	1	10197
3445	1	3454
3445	1	3455

3444	1	3460
3444	1	3459
3444	1	5721
3444	1	5720
3444	1	10197
3444	1	3454
3444	1	3455
3443	1	3460
3443	1	3459
3443	1	5721
3443	1	5720
3443	1	10197
3443	1	3454
3443	1	3455
3442	1	3460
3442	1	3459
3442	1	5721
3442	1	5720
3442	1	10197
3442	1	3454
3442	1	3455
3441	1	3460
3441	1	3459
3441	1	5721
3441	1	5720
3441	1	10197
3441	1	3454
3441	1	3455
3440	1	3460
3440	1	3459
3440	1	5721
3440	1	5720
3440	1	10197
3440	1	3454
3440	1	3455
3439	1	3460
3439	1	3459
3439	1	5721
3439	1	5720
3439	1	10197
3439	1	3454
3439	1	3455
2322	-1	6688
2322	-1	1050
2322	1	6777
2322	1	6776
2322	1	6774
2322	1	2885
2322	1	8503
2322	1	5296
2322	1	5295
2322	1	5294
2322	1	5293
2322	1	5291
2322	1	5290

2322	1	23533
2323	1	2322
3815	1	6777
3815	1	6776
3815	1	6774
3815	1	2885
3815	1	8503
3815	1	5296
3815	1	5295
3815	1	5294
3815	1	5293
3815	1	5291
3815	1	5290
3815	1	23533
3815	1	4893
3815	1	3845
3815	1	3265
4254	1	3815
4233	-1	1499
4233	-1	1500
4233	1	2549
4233	1	4893
4233	1	3845
4233	1	3265
4233	1	2885
4233	1	6464
4233	1	53358
4233	1	399694
4233	1	25759
4233	1	8503
4233	1	5296
4233	1	5295
4233	1	5294
4233	1	5293
4233	1	5291
4233	1	5290
4233	1	23533
4233	1	5747
3082	1	2260
3082	1	5159
3082	1	5156
3082	1	3791
3082	1	3480
3082	1	2324
3082	1	2321
3082	1	2064
3082	1	1956
3082	1	4233
2324	1	6464
2324	1	53358
2324	1	399694
2324	1	25759
2324	1	8503
2324	1	5296
2324	1	5295

2324	1	5294
2324	1	5293
2324	1	5291
2324	1	5290
2324	1	23533
2324	1	5747
7424	1	5159
7424	1	5156
7424	1	4233
7424	1	3480
7424	1	2321
7424	1	2064
7424	1	1956
7424	1	2324
7424	1	3791
2277	1	5159
2277	1	5156
2277	1	4233
2277	1	3480
2277	1	2321
2277	1	2064
2277	1	1956
2277	1	2324
2277	1	3791
3791	1	6464
3791	1	53358
3791	1	399694
3791	1	6714
3791	1	8503
3791	1	5296
3791	1	5295
3791	1	5294
3791	1	5293
3791	1	5291
3791	1	5290
3791	1	23533
3791	1	998
3791	1	5829
3791	1	5747
3791	1	25759
3791	1	5336
3791	1	5335
3791	1	9047
7422	1	5159
7422	1	5156
7422	1	4233
7422	1	3480
7422	1	2324
7422	1	2064
7422	1	1956
7422	1	3791
7422	1	2321
2321	1	6464
2321	1	53358
2321	1	399694

2321	1	25759
2321	1	8503
2321	1	5296
2321	1	5295
2321	1	5294
2321	1	5293
2321	1	5291
2321	1	5290
2321	1	23533
2321	1	5747
7423	1	5159
7423	1	5156
7423	1	4233
7423	1	3791
7423	1	3480
7423	1	2324
7423	1	2064
7423	1	1956
7423	1	2321
80301	1	5159
56034	1	3645
56034	1	2263
56034	1	2260
56034	1	5159
56034	1	4233
56034	1	3791
56034	1	3480
56034	1	2324
56034	1	2321
56034	1	2064
56034	1	1956
56034	1	5156
7173	1	4352
5617	1	5618
2689	1	2690
2688	1	2690
2056	1	2057
85480	1	3575
85480	1	64109
59067	1	50615
59067	1	3561
3600	1	3601
3600	1	3560
3600	1	3561
3578	1	3581
3578	1	3561
3574	1	3575
3574	1	3561
3558	1	3559
3558	1	3560
3558	1	3561
3567	1	3568
3567	1	1439
3562	1	3563
3562	1	1439

1437	1	1438
1437	1	1439
51561	1	149233
51561	1	3594
3593	1	3594
3593	1	3595
3592	1	3594
3592	1	3595
3596	1	3566
3596	1	3597
3565	1	3561
3565	1	3566
3565	1	3597
3952	1	3953
1440	1	1441
1489	1	3572
1489	1	3977
23529	1	1271
23529	1	3572
23529	1	3977
1270	1	1271
1270	1	3572
1270	1	3977
3976	1	3572
3976	1	3977
5008	1	9180
5008	1	3977
5008	1	3572
3589	1	3590
3589	1	3572
3569	1	3570
3569	1	3572
7852	1	2773
7852	1	2771
7852	1	2770
6387	1	7852
887	1	9630
887	1	2776
887	1	2769
887	1	2767
886	1	9630
886	1	2776
886	1	2769
886	1	2767
7201	1	9630
7201	1	2776
7201	1	2769
7201	1	2767
6915	1	9630
6915	1	2776
6915	1	2769
6915	1	2767
6870	1	9630
6870	1	2776
6870	1	2769

6870	1	2767
6869	1	9630
6869	1	2776
6869	1	2769
6869	1	2767
6865	1	9630
6865	1	2776
6865	1	2769
6865	1	2767
624	1	55970
624	1	9630
624	1	2776
624	1	2769
624	1	2767
623	1	55970
623	1	9630
623	1	2776
623	1	2769
623	1	2767
5737	1	9630
5737	1	2776
5737	1	2769
5737	1	2767
5733	1	9630
5733	1	2776
5733	1	2769
5733	1	2767
5731	1	9630
5731	1	2776
5731	1	2769
5731	1	2767
5724	1	9630
5724	1	2776
5724	1	2769
5724	1	2767
57105	1	9630
57105	1	2776
57105	1	2769
57105	1	2767
56413	1	9630
56413	1	2776
56413	1	2769
56413	1	2767
553	1	9630
553	1	2776
553	1	2769
553	1	2767
552	1	9630
552	1	2776
552	1	2769
552	1	2767
5021	1	9630
5021	1	2776
5021	1	2769
5021	1	2767

4923	1	9630
4923	1	2776
4923	1	2769
4923	1	2767
3973	1	9630
3973	1	2776
3973	1	2769
3973	1	2767
3358	1	9630
3358	1	2776
3358	1	2769
3358	1	2767
3357	1	9630
3357	1	2776
3357	1	2769
3357	1	2767
3356	1	9630
3356	1	2776
3356	1	2769
3356	1	2767
3269	1	9630
3269	1	2776
3269	1	2769
3269	1	2767
2925	1	9630
2925	1	2776
2925	1	2769
2925	1	2767
2915	1	2781
2915	1	2778
2915	1	2775
2915	1	2773
2915	1	2771
2915	1	2770
2915	1	2768
2915	1	10672
2915	1	9630
2915	1	2776
2915	1	2769
2915	1	2767
2911	1	2781
2911	1	2778
2911	1	2775
2911	1	2773
2911	1	2771
2911	1	2770
2911	1	2768
2911	1	10672
2911	1	9630
2911	1	2776
2911	1	2769
2911	1	2767
2149	1	2768
2149	1	10672
2149	1	9630

2149	1	2776
2149	1	2769
2149	1	2767
1910	1	2775
1910	1	2773
1910	1	2771
1910	1	2770
1910	1	9630
1910	1	2776
1910	1	2769
1910	1	2767
1909	1	9630
1909	1	2776
1909	1	2769
1909	1	2767
185	1	9630
185	1	2776
185	1	2769
185	1	2767
148	1	9630
148	1	2776
148	1	2769
148	1	2767
147	1	9630
147	1	2776
147	1	2769
147	1	2767
146	1	9630
146	1	2776
146	1	2769
146	1	2767
1129	1	55970
1129	1	9630
1129	1	2776
1129	1	2769
1129	1	2767
9630	1	5332
9630	1	5331
9630	1	5330
9630	1	23236
2776	1	1857
2776	1	1856
2776	1	1855
2776	1	5332
2776	1	5331
2776	1	5330
2776	1	23236
2769	1	5332
2769	1	5331
2769	1	5330
2769	1	23236
2767	1	5332
2767	1	5331
2767	1	5330
2767	1	23236

10800	1	9630
10800	1	2776
10800	1	2769
10800	1	2767
810	1	5837
810	1	5836
810	1	5834
810	1	814
810	1	4893
810	1	3845
810	1	3265
810	1	818
810	1	817
810	1	816
810	1	815
810	1	63928
810	1	5535
810	1	5534
810	1	5533
810	1	5532
810	1	5530
810	1	11261
810	1	114
810	1	109
810	1	107
810	1	5153
810	1	5137
810	1	5136
810	1	91807
810	1	85366
810	1	4638
808	1	5837
808	1	5836
808	1	5834
808	1	814
808	1	4893
808	1	3845
808	1	3265
808	1	818
808	1	817
808	1	816
808	1	815
808	1	63928
808	1	5535
808	1	5534
808	1	5533
808	1	5532
808	1	5530
808	1	11261
808	1	114
808	1	109
808	1	107
808	1	5153
808	1	5137
808	1	5136

808	1	91807
808	1	85366
808	1	4638
805	1	5837
805	1	5836
805	1	5834
805	1	814
805	1	4893
805	1	3845
805	1	3265
805	1	818
805	1	817
805	1	816
805	1	815
805	1	63928
805	1	5535
805	1	5534
805	1	5533
805	1	5532
805	1	5530
805	1	11261
805	1	114
805	1	109
805	1	107
805	1	5153
805	1	5137
805	1	5136
805	1	91807
805	1	85366
805	1	4638
801	1	5837
801	1	5836
801	1	5834
801	1	814
801	1	4893
801	1	3845
801	1	3265
801	1	818
801	1	817
801	1	816
801	1	815
801	1	63928
801	1	5535
801	1	5534
801	1	5533
801	1	5532
801	1	5530
801	1	11261
801	1	114
801	1	109
801	1	107
801	1	5153
801	1	5137
801	1	5136
801	1	91807

801	1	85366
801	1	4638
51806	1	5837
51806	1	5836
51806	1	5834
51806	1	814
51806	1	4893
51806	1	3845
51806	1	3265
51806	1	818
51806	1	817
51806	1	816
51806	1	815
51806	1	63928
51806	1	5535
51806	1	5534
51806	1	5533
51806	1	5532
51806	1	5530
51806	1	11261
51806	1	114
51806	1	109
51806	1	107
51806	1	5153
51806	1	5137
51806	1	5136
51806	1	91807
51806	1	85366
51806	1	4638
91807	1	93408
91807	1	58498
91807	1	4636
91807	1	4633
91807	1	29895
91807	1	10627
91807	1	10398
91807	1	103910
85366	1	93408
85366	1	58498
85366	1	4636
85366	1	4633
85366	1	29895
85366	1	10627
85366	1	10398
85366	1	103910
4638	1	93408
4638	1	58498
4638	1	4636
4638	1	4633
4638	1	29895
4638	1	10627
4638	1	10398
4638	1	103910
163688	1	5837
163688	1	5836

163688	1	5834
163688	1	814
163688	1	4893
163688	1	3845
163688	1	3265
163688	1	818
163688	1	817
163688	1	816
163688	1	815
163688	1	63928
163688	1	5535
163688	1	5534
163688	1	5533
163688	1	5532
163688	1	5530
163688	1	11261
163688	1	114
163688	1	109
163688	1	107
163688	1	5153
163688	1	5137
163688	1	5136
163688	1	91807
163688	1	85366
163688	1	4638
5336	1	7410
5336	1	7409
5336	1	10451
5336	1	4846
5336	1	8681
5336	1	84647
5336	1	8399
5336	1	8398
5336	1	81579
5336	1	64600
5336	1	5322
5336	1	5321
5336	1	5320
5336	1	5319
5336	1	50487
5336	1	30814
5336	1	26279
5336	1	1E+08
5336	1	5582
5336	1	5579
5336	1	5578
5336	1	63928
5336	1	5535
5336	1	5534
5336	1	5533
5336	1	5532
5336	1	5530
5336	1	11261
5335	1	7410
5335	1	7409

5335	1	10451
5335	1	4846
5335	1	8681
5335	1	84647
5335	1	8399
5335	1	8398
5335	1	81579
5335	1	64600
5335	1	5322
5335	1	5321
5335	1	5320
5335	1	5319
5335	1	50487
5335	1	30814
5335	1	26279
5335	1	1E+08
5335	1	5582
5335	1	5579
5335	1	5578
5335	1	63928
5335	1	5535
5335	1	5534
5335	1	5533
5335	1	5532
5335	1	5530
5335	1	11261
3363	1	2778
3363	1	2774
3362	1	2778
3362	1	2774
3361	1	2778
3361	1	2774
3360	1	2778
3360	1	2774
3274	1	2778
3274	1	2774
1816	1	2778
1816	1	2774
1812	1	2778
1812	1	2774
155	1	2778
155	1	2774
154	1	2778
154	1	2774
153	1	2778
153	1	2774
136	1	2778
136	1	2774
135	1	2778
135	1	2774
1133	1	55970
1133	1	2778
1133	1	2774
1131	1	55970
1131	1	9630

1131	1	2776
1131	1	2769
1131	1	2767
1131	1	2778
1131	1	2774
2778	1	8681
2778	1	84647
2778	1	8399
2778	1	8398
2778	1	81579
2778	1	64600
2778	1	5322
2778	1	5321
2778	1	5320
2778	1	5319
2778	1	50487
2778	1	30814
2778	1	26279
2778	1	1E+08
2778	1	112
2778	1	111
2778	1	196883
2778	1	115
2778	1	114
2778	1	113
2778	1	109
2778	1	108
2778	1	107
2774	1	196883
2774	1	115
2774	1	114
2774	1	113
2774	1	109
2774	1	108
2774	1	107
1128	1	55970
1128	1	9630
1128	1	2776
1128	1	2769
1128	1	2767
1128	1	2778
1128	1	2774
2549	1	2885
2549	1	5781
2549	1	1399
2549	1	1398
2549	1	8503
2549	1	5296
2549	1	5295
2549	1	5294
2549	1	5293
2549	1	5291
2549	1	5290
2549	1	23533
369	1	5605

369	1	5604
6464	1	2885
53358	1	2885
399694	1	2885
25759	1	2885
8440	1	5747
8440	1	57144
8440	1	56924
8440	1	5063
8440	1	5062
8440	1	5058
8440	1	10298
57144	-1	91807
57144	-1	85366
57144	-1	4638
57144	1	3985
57144	1	3984
56924	-1	91807
56924	-1	85366
56924	-1	4638
56924	1	3985
56924	1	3984
5063	-1	91807
5063	-1	85366
5063	-1	4638
5063	1	3985
5063	1	3984
10298	-1	91807
10298	-1	85366
10298	-1	4638
10298	1	3985
10298	1	3984
4690	1	5881
4690	1	5880
4690	1	5879
4690	1	57144
4690	1	56924
4690	1	5063
4690	1	5062
4690	1	5058
4690	1	10298
1399	1	8503
1399	1	5296
1399	1	5295
1399	1	5294
1399	1	5293
1399	1	5291
1399	1	5290
1399	1	23533
1399	1	23433
1399	1	5829
1399	1	2889
1399	1	1793
1399	1	27
1399	1	25

25	1	6464
25	1	53358
25	1	399694
25	1	25759
25	1	868
25	1	867
25	1	23624
25	1	1399
25	1	1398
25	1	2885
25	1	6777
25	1	6776
1398	1	8503
1398	1	5296
1398	1	5295
1398	1	5294
1398	1	5293
1398	1	5291
1398	1	5290
1398	1	23533
1398	1	23433
1398	1	5829
1398	1	2889
1398	1	1793
1398	1	27
1398	1	25
8503	1	5170
8503	1	7294
8503	1	3702
8503	1	5881
8503	1	5880
8503	1	5879
5296	1	5170
5296	1	7294
5296	1	3702
5296	1	5881
5296	1	5880
5296	1	5879
5295	1	5170
5295	1	7294
5295	1	3702
5295	1	5881
5295	1	5880
5295	1	5879
5294	1	5170
5294	1	7294
5294	1	3702
5294	1	5881
5294	1	5880
5294	1	5879
5293	1	5170
5293	1	7294
5293	1	3702
5293	1	5881
5293	1	5880

5293	1	5879
5291	1	5170
5291	1	7294
5291	1	3702
5291	1	5881
5291	1	5880
5291	1	5879
5290	1	5170
5290	1	7294
5290	1	3702
5290	1	5881
5290	1	5880
5290	1	5879
23533	1	5170
23533	1	7294
23533	1	3702
23533	1	5881
23533	1	5880
23533	1	5879
2064	-1	1499
2064	-1	1500
2064	1	6464
2064	1	53358
2064	1	399694
2064	1	25759
2064	1	3716
2064	1	5747
2064	1	5336
2064	1	5335
2064	1	8503
2064	1	5296
2064	1	5295
2064	1	5294
2064	1	5293
2064	1	5291
2064	1	5290
2064	1	23533
2064	1	2885
2885	1	9846
2885	1	2549
2885	1	6655
2885	1	6654
6714	-1	1499
6714	-1	387
6714	1	1956
6714	1	9855
6714	1	5747
6714	1	10746
6714	1	8503
6714	1	5296
6714	1	5295
6714	1	5294
6714	1	5293
6714	1	5291
6714	1	5290

6714	1	23533
145957	1	2066
10718	1	2066
9542	1	2066
9542	1	2065
2065	1	6464
2065	1	53358
2065	1	399694
2065	1	25759
2065	1	5336
2065	1	5335
2065	1	8503
2065	1	5296
2065	1	5295
2065	1	5294
2065	1	5293
2065	1	5291
2065	1	5290
2065	1	23533
2065	1	2885
3084	1	2066
3084	1	2065
2069	1	2066
2069	1	1956
1839	1	2066
1839	1	1956
2066	1	6777
2066	1	6776
2066	1	6464
2066	1	53358
2066	1	399694
2066	1	25759
2066	1	5336
2066	1	5335
2066	1	8503
2066	1	5296
2066	1	5295
2066	1	5294
2066	1	5293
2066	1	5291
2066	1	5290
2066	1	23533
2066	1	2885
685	1	2066
685	1	1956
374	1	1956
7039	1	5159
7039	1	5156
7039	1	3645
7039	1	3480
7039	1	2263
7039	1	2260
7039	1	2064
7039	1	1956
4616	-1	983

4616	-1	9133
4616	-1	891
4616	-1	85417
4616	1	5111
4616	1	4216
1647	-1	983
1647	-1	9133
1647	-1	891
1647	-1	85417
1647	1	5111
1647	1	4216
4216	1	650832
4216	1	5608
4216	1	5606
10912	-1	983
10912	-1	9133
10912	-1	891
10912	-1	85417
10912	1	5111
10912	1	4216
5062	-1	91807
5062	-1	85366
5062	-1	4638
5062	1	3985
5062	1	3984
5058	-1	91807
5058	-1	85366
5058	-1	4638
5058	1	5605
5058	1	3985
5058	1	3984
5058	1	9020
998	1	81624
998	1	128239
998	1	10788
998	1	84612
998	1	84552
998	1	50855
998	1	8826
998	1	7454
998	1	8976
998	1	6300
998	1	5603
998	1	5600
998	1	1432
998	1	57144
998	1	56924
998	1	5063
998	1	10298
998	1	5062
998	1	5058
998	1	4214
5881	-1	26999
5881	-1	23191
5881	-1	3071

5881	-1	10787
5881	-1	10152
5881	1	5970
5881	1	4790
5881	1	128239
5881	1	10788
5881	1	650832
5881	1	5608
5881	1	5606
5881	1	6416
5881	1	5609
5881	1	10458
5881	1	8936
5881	1	10810
5881	1	10163
5881	1	8826
5881	1	57144
5881	1	56924
5881	1	5063
5881	1	10298
5881	1	84448
5881	1	3983
5881	1	22885
5881	1	5602
5881	1	5601
5881	1	5599
5881	1	5062
5881	1	5058
5881	1	4214
5880	-1	26999
5880	-1	23191
5880	-1	3071
5880	-1	10787
5880	-1	10152
5880	1	5970
5880	1	4790
5880	1	128239
5880	1	10788
5880	1	650832
5880	1	5608
5880	1	5606
5880	1	6416
5880	1	5609
5880	1	10458
5880	1	8936
5880	1	10810
5880	1	10163
5880	1	8826
5880	1	57144
5880	1	56924
5880	1	5063
5880	1	10298
5880	1	84448
5880	1	3983
5880	1	22885

5880	1	5602
5880	1	5601
5880	1	5599
5880	1	5062
5880	1	5058
5880	1	4214
4214	1	5609
4214	1	650832
4214	1	5608
4214	1	5606
5879	-1	26999
5879	-1	23191
5879	-1	3071
5879	-1	10787
5879	-1	10152
5879	1	5970
5879	1	4790
5879	1	128239
5879	1	10788
5879	1	4689
5879	1	653361
5879	1	4688
5879	1	1535
5879	1	50508
5879	1	27035
5879	1	1536
5879	1	650832
5879	1	5608
5879	1	5606
5879	1	6416
5879	1	5609
5879	1	10458
5879	1	8936
5879	1	10810
5879	1	10163
5879	1	8826
5879	1	57144
5879	1	56924
5879	1	5063
5879	1	10298
5879	1	84448
5879	1	3983
5879	1	22885
5879	1	5602
5879	1	5601
5879	1	5599
5879	1	5062
5879	1	5058
5879	1	4214
5894	-1	572
6655	1	10125
6655	1	6237
6655	1	4893
6655	1	3845
6655	1	3265

6655	1	22808
6655	1	22800
6654	1	10125
6654	1	6237
6654	1	4893
6654	1	3845
6654	1	3265
6654	1	22808
6654	1	22800
1616	1	4217
7186	1	9020
7186	1	4214
7186	1	4217
7186	1	5871
836	1	1630
836	1	1676
836	1	839
836	1	4214
836	1	5062
836	1	5058
836	1	6789
836	1	6788
7043	1	91
7043	1	7048
7043	1	7046
7043	1	130399
7042	1	91
7042	1	7048
7042	1	7046
7042	1	130399
91	1	1616
7048	1	5519
7048	1	5518
7048	1	5516
7048	1	5515
7048	1	387
7048	1	1616
7046	1	1616
130399	1	1616
7040	1	91
7040	1	7048
7040	1	7046
7040	1	130399
355	1	8772
355	1	1616
356	1	8771
356	1	355
3553	1	3556
3553	1	7850
3553	1	3554
7850	1	836
3554	1	4615
3554	1	51135
3554	1	3656
3554	1	3654

3554	1	11213
3554	1	8717
3554	1	8772
3554	1	836
3552	1	3556
3552	1	7850
3552	1	3554
7132	1	1616
7132	1	8737
7132	1	8717
7132	1	8772
7132	1	7186
7132	1	836
7124	1	7133
7124	1	7132
5908	-1	387
5908	1	5894
5908	1	369
5908	1	3684
5908	1	3689
5908	1	3683
5908	1	3676
5908	1	3688
5908	1	5602
5908	1	5601
5908	1	5599
5908	1	673
5906	-1	387
5906	1	5894
5906	1	369
5906	1	3684
5906	1	3689
5906	1	3683
5906	1	3676
5906	1	3688
5906	1	5602
5906	1	5601
5906	1	5599
5906	1	673
25780	1	6237
25780	1	4893
25780	1	3845
25780	1	3265
25780	1	22808
25780	1	22800
115727	1	6237
115727	1	4893
115727	1	3845
115727	1	3265
115727	1	22808
115727	1	22800
10235	1	6237
10235	1	4893
10235	1	3845
10235	1	3265

10235	1	22808
10235	1	22800
10125	1	6237
10125	1	4893
10125	1	3845
10125	1	3265
10125	1	22808
10125	1	22800
5924	1	6237
5924	1	4893
5924	1	3845
5924	1	3265
5924	1	22808
5924	1	22800
5923	1	6237
5923	1	4893
5923	1	3845
5923	1	3265
5923	1	22808
5923	1	22800
6237	1	9459
6237	1	8503
6237	1	5296
6237	1	5295
6237	1	5294
6237	1	5293
6237	1	5291
6237	1	5290
6237	1	23533
6237	1	369
6237	1	4301
6237	1	4342
6237	1	5894
6237	1	673
4893	1	5900
4893	1	11186
4893	1	9459
4893	1	8503
4893	1	5296
4893	1	5295
4893	1	5294
4893	1	5293
4893	1	5291
4893	1	5290
4893	1	23533
4893	1	4301
4893	1	369
4893	1	4342
4893	1	5894
4893	1	673
3845	1	5900
3845	1	11186
3845	1	9459
3845	1	8503
3845	1	5296

3845	1	5295
3845	1	5294
3845	1	5293
3845	1	5291
3845	1	5290
3845	1	23533
3845	1	4301
3845	1	369
3845	1	4342
3845	1	5894
3845	1	673
3265	1	5900
3265	1	11186
3265	1	9459
3265	1	4301
3265	1	8503
3265	1	5296
3265	1	5295
3265	1	5294
3265	1	5293
3265	1	5291
3265	1	5290
3265	1	23533
3265	1	369
3265	1	4342
3265	1	5894
3265	1	673
22808	1	9459
22808	1	8503
22808	1	5296
22808	1	5295
22808	1	5294
22808	1	5293
22808	1	5291
22808	1	5290
22808	1	23533
22808	1	369
22808	1	4301
22808	1	4342
22808	1	5894
22808	1	673
22800	1	9459
22800	1	8503
22800	1	5296
22800	1	5295
22800	1	5294
22800	1	5293
22800	1	5291
22800	1	5290
22800	1	23533
22800	1	369
22800	1	4301
22800	1	4342
22800	1	5894
22800	1	673

9693	1	6237
9693	1	4893
9693	1	3845
9693	1	3265
9693	1	22808
9693	1	22800
55970	1	57144
55970	1	56924
55970	1	5063
55970	1	5062
55970	1	5058
55970	1	10298
55970	1	89846
55970	1	2245
55970	1	5922
5922	-1	6237
5922	-1	4893
5922	-1	3845
5922	-1	3265
5922	-1	22808
5922	-1	22800
2768	1	9138
2768	1	23365
2768	1	8681
2768	1	84647
2768	1	8399
2768	1	8398
2768	1	81579
2768	1	64600
2768	1	5322
2768	1	5321
2768	1	5320
2768	1	5319
2768	1	50487
2768	1	30814
2768	1	26279
2768	1	1E+08
2768	1	5922
5155	1	3645
5155	1	2263
5155	1	2260
5155	1	4233
5155	1	3791
5155	1	3480
5155	1	2324
5155	1	2321
5155	1	2064
5155	1	1956
5155	1	5159
5155	1	5156
5159	1	4893
5159	1	3845
5159	1	3265
5159	1	6655
5159	1	6654

5159	1	6714
5159	1	2885
5159	1	6464
5159	1	53358
5159	1	399694
5159	1	25759
5159	1	8503
5159	1	5296
5159	1	5295
5159	1	5294
5159	1	5293
5159	1	5291
5159	1	5290
5159	1	23533
5159	1	5747
5159	1	5336
5159	1	5335
5156	1	4893
5156	1	3845
5156	1	3265
5156	1	6655
5156	1	6654
5156	1	6714
5156	1	2885
5156	1	6464
5156	1	53358
5156	1	399694
5156	1	25759
5156	1	8503
5156	1	5296
5156	1	5295
5156	1	5294
5156	1	5293
5156	1	5291
5156	1	5290
5156	1	23533
5156	1	5747
5156	1	5336
5156	1	5335
5154	1	3645
5154	1	2263
5154	1	2260
5154	1	4233
5154	1	3791
5154	1	3480
5154	1	2324
5154	1	2321
5154	1	2064
5154	1	1956
5154	1	5159
5154	1	5156
9965	1	5159
9965	1	5156
9965	1	4233
9965	1	3480

9965	1	1956
9965	1	2264
9965	1	2263
9965	1	2261
9965	1	2260
8823	1	5159
8823	1	5156
8823	1	4233
8823	1	3480
8823	1	1956
8823	1	2264
8823	1	2263
8823	1	2261
8823	1	2260
8822	1	5159
8822	1	5156
8822	1	4233
8822	1	3480
8822	1	1956
8822	1	2264
8822	1	2263
8822	1	2261
8822	1	2260
8817	1	5159
8817	1	5156
8817	1	4233
8817	1	3480
8817	1	1956
8817	1	2264
8817	1	2263
8817	1	2261
8817	1	2260
8074	1	5159
8074	1	5156
8074	1	4233
8074	1	3480
8074	1	1956
8074	1	2264
8074	1	2263
8074	1	2261
8074	1	2260
27006	1	5159
27006	1	5156
27006	1	4233
27006	1	3480
27006	1	1956
27006	1	2264
27006	1	2263
27006	1	2261
27006	1	2260
26291	1	5159
26291	1	5156
26291	1	4233
26291	1	3480
26291	1	1956

26291	1	2264
26291	1	2263
26291	1	2261
26291	1	2260
26281	1	5159
26281	1	5156
26281	1	4233
26281	1	3480
26281	1	1956
26281	1	2264
26281	1	2263
26281	1	2261
26281	1	2260
2259	1	5159
2259	1	5156
2259	1	4233
2259	1	3480
2259	1	1956
2259	1	2264
2259	1	2263
2259	1	2261
2259	1	2260
2258	1	5159
2258	1	5156
2258	1	4233
2258	1	3480
2258	1	1956
2258	1	2264
2258	1	2263
2258	1	2261
2258	1	2260
2257	1	5159
2257	1	5156
2257	1	4233
2257	1	3480
2257	1	1956
2257	1	2264
2257	1	2263
2257	1	2261
2257	1	2260
2256	1	5159
2256	1	5156
2256	1	4233
2256	1	3480
2256	1	1956
2256	1	2264
2256	1	2263
2256	1	2261
2256	1	2260
2255	1	5159
2255	1	5156
2255	1	4233
2255	1	3480
2255	1	1956
2255	1	2264

2255	1	2263
2255	1	2261
2255	1	2260
2254	1	5159
2254	1	5156
2254	1	4233
2254	1	3480
2254	1	1956
2254	1	2264
2254	1	2263
2254	1	2261
2254	1	2260
2253	1	5159
2253	1	5156
2253	1	4233
2253	1	3480
2253	1	1956
2253	1	2264
2253	1	2263
2253	1	2261
2253	1	2260
2252	1	5159
2252	1	5156
2252	1	4233
2252	1	3480
2252	1	1956
2252	1	2264
2252	1	2263
2252	1	2261
2252	1	2260
2251	1	5159
2251	1	5156
2251	1	4233
2251	1	3480
2251	1	1956
2251	1	2264
2251	1	2263
2251	1	2261
2251	1	2260
2250	1	5159
2250	1	5156
2250	1	4233
2250	1	3480
2250	1	1956
2250	1	2264
2250	1	2263
2250	1	2261
2250	1	2260
2249	1	5159
2249	1	5156
2249	1	4233
2249	1	3480
2249	1	1956
2249	1	2264
2249	1	2263

2249	1	2261
2249	1	2260
2248	1	5159
2248	1	5156
2248	1	4233
2248	1	3480
2248	1	1956
2248	1	2264
2248	1	2263
2248	1	2261
2248	1	2260
2247	1	5159
2247	1	5156
2247	1	4233
2247	1	3480
2247	1	1956
2247	1	2264
2247	1	2263
2247	1	2261
2247	1	2260
2264	1	6655
2264	1	6654
2263	1	8503
2263	1	5296
2263	1	5295
2263	1	5294
2263	1	5293
2263	1	5291
2263	1	5290
2263	1	23533
2263	1	2885
2263	1	6655
2263	1	6654
2261	1	2885
2261	1	6655
2261	1	6654
2260	1	4893
2260	1	3845
2260	1	3265
2260	1	8503
2260	1	5296
2260	1	5295
2260	1	5294
2260	1	5293
2260	1	5291
2260	1	5290
2260	1	23533
2260	1	2885
2260	1	6655
2260	1	6654
2246	1	5159
2246	1	5156
2246	1	4233
2246	1	3480
2246	1	1956

2246	1	2264
2246	1	2263
2246	1	2261
2246	1	2260
1956	-1	1499
1956	-1	1500
1956	1	6464
1956	1	53358
1956	1	399694
1956	1	25759
1956	1	1399
1956	1	1398
1956	1	6777
1956	1	6776
1956	1	868
1956	1	867
1956	1	23624
1956	1	5336
1956	1	5335
1956	1	4893
1956	1	3845
1956	1	3265
1956	1	3716
1956	1	6655
1956	1	6654
1956	1	8503
1956	1	5296
1956	1	5295
1956	1	5294
1956	1	5293
1956	1	5291
1956	1	5290
1956	1	23533
1956	1	5747
1956	1	2885
1956	1	6714
1950	1	3645
1950	1	2263
1950	1	2260
1950	1	5159
1950	1	5156
1950	1	4233
1950	1	3791
1950	1	3480
1950	1	2324
1950	1	2321
1950	1	2064
1950	1	1956

Table S2. KEGG-HPO associated. Degree of pleiotropy here represents the number of associated phenotypes from HPO database.

Entrez Gene ID	Gene Name	Degree of pleiotropy
10000	AKT3	126
10095	ARPC1B	56
10125	RASGRP1	285
1019	CDK4	79
102	ADAM10	9
1020	CDK5	102
1021	CDK6	102
10235	RASGRP2	22
1026	CDKN1A	168
1027	CDKN1B	211
1028	CDKN1C	297
1029	CDKN2A	196
10297	APC2	272
1030	CDKN2B	200
1031	CDKN2C	168
10319	LAMC3	35
10452	TOMM40	84
1050	CEBPA	16
10683	DLL3	234
107	ADCY1	13
1073	CFL2	207
1080	CFTR	281
10842	PPP1R17	34
10892	MALT1	106
109	ADCY3	26
111	ADCY5	79
112	ADCY6	62
1131	CHRM3	188
114609	TIRAP	15
1147	CHUK	86
1277	COL1A1	606
1278	COL1A2	374
1280	COL2A1	864
1281	COL3A1	563
1282	COL4A1	374
1284	COL4A2	47
1286	COL4A4	79
1288	COL4A6	15
1289	COL5A1	364
1290	COL5A2	332
1291	COL6A1	302
1292	COL6A2	327
1293	COL6A3	325

1301	COL11A1	532
1302	COL11A2	394
1311	COMP	275
1385	CREB1	7
1387	CREBBP	572
1399	CRKL	320
1407	CRY1	8
1438	CSF2RA	71
1439	CSF2RB	72
1441	CSF3R	19
1453	CSNK1D	17
1457	CSNK2A1	170
148022	TICAM1	12
1487	CTBP1	491
149233	IL23R	287
1493	CTLA4	427
1499	CTNNB1	491
1500	CTNND1	101
1535	CYBA	196
1536	CYBB	203
154	ADRB2	28
155	ADRB3	20
1605	DAG1	283
1616	DAXX	136
1630	DCC	296
1634	DCN	24
1729	DIAPH1	82
1730	DIAPH2	21
1813	DRD2	35
1816	DRD5	30
182	JAG1	355
1848	DUSP6	286
185	AGTR1	77
1855	DVL1	494
1857	DVL3	526
1906	EDN1	177
1909	EDNRA	159
1910	EDNRB	232
1947	EFNB1	325
1950	EGF	84
1956	EGFR	90
1969	EPHA2	23
2	A2M	34
200576	PIKFYVE	16
2033	EP300	627
2043	EPHA4	83
2048	EPHB2	18

2050	EPHB4	80
2056	EPO	19
2057	EPOR	52
2064	ERBB2	54
2065	ERBB3	121
2066	ERBB4	98
207	AKT1	693
208	AKT2	128
2122	MECOM	90
2147	F2	146
2153	F5	171
2155	F7	90
2157	F8	37
2158	F9	49
2159	F10	112
2160	F11	50
2161	F12	59
2162	F13A1	157
2165	F13B	134
2206	MS4A2	36
2243	FGA	124
2244	FGB	78
2245	FGD1	321
2248	FGF3	226
2250	FGF5	23
2253	FGF8	408
2254	FGF9	141
2255	FGF10	250
2257	FGF12	216
2259	FGF14	107
2260	FGFR1	1043
2261	FGFR3	1010
2263	FGFR2	1137
2266	FGG	78
22926	ATF6	82
2308	FOXO1	14
2322	FLT3	21
23236	PLCB1	48
2324	FLT4	125
2335	FN1	186
23385	NCSTN	19
23396	PIP5K1C	26
23529	CLCF1	179
2353	FOS	312
23533	PIK3R5	127
2475	MTOR	333
2488	FSHB	117

2492	FSHR	186
25	ABL1	234
2535	FZD2	427
2549	GAB1	11
26281	FGF20	113
268	AMH	35
2688	GH1	46
269	AMHR2	35
2690	GHR	207
2697	GJA1	769
26999	CYFIP2	207
27040	LAT	49
2735	GLI1	244
2736	GLI2	407
2737	GLI3	714
2767	GNA11	245
2771	GNAI2	20
2773	GNAI3	193
2774	GNAL	28
2775	GNAO1	278
2776	GNAQ	286
2778	GNAS	638
2782	GNB1	153
2784	GNB3	55
2796	GNRH1	198
2798	GNRHR	184
2811	GP1BA	194
2812	GP1BB	638
2815	GP9	119
284217	LAMA1	84
28514	DLL1	189
2892	GRIA3	69
2911	GRM1	137
29110	TBK1	242
2934	GSN	219
29851	ICOS	204
2997	GYS1	40
2998	GYS2	70
3053	SERPIND1	22
3082	HGF	13
31	ACACA	14
3105	HLA-A	90
3106	HLA-B	456
3107	HLA-C	25
3135	HLA-G	11
324	APC	669
3265	HRAS	679

330	BIRC3	84
331	XIAP	164
3339	HSPG2	549
3356	HTR2A	43
3371	TNC	7
3455	IFNAR2	8
3458	IFNG	212
3459	IFNGR1	143
3460	IFNGR2	11
3479	IGF1	205
3480	IGF1R	261
3516	RBPJ	264
355	FAS	556
3551	IKBKB	61
3553	IL1B	34
3559	IL2RA	167
356	FASLG	319
3560	IL2RB	68
3561	IL2RG	273
3566	IL4R	36
3569	IL6	180
3574	IL7	45
3575	IL7R	175
3586	IL10	293
3587	IL10RA	48
3588	IL10RB	49
3590	IL11RA	92
3592	IL12A	395
3593	IL12B	145
3594	IL12RB1	149
3596	IL13	11
3624	INHBA	103
3630	INS	269
3643	INSR	369
3654	IRAK1	186
3655	ITGA6	164
3661	IRF3	22
3663	IRF5	258
3665	IRF7	6
3667	IRS1	22
3673	ITGA2	95
3674	ITGA2B	158
3675	ITGA3	158
3679	ITGA7	251
3689	ITGB2	65
3690	ITGB3	158
3691	ITGB4	266

3694	ITGB6	152
3702	ITK	77
3717	JAK2	331
3718	JAK3	173
3745	KCNB1	193
375790	AGRN	364
3784	KCNQ1	279
3791	KDR	10
3815	KIT	314
3818	KLKB1	6
3845	KRAS	1153
3908	LAMA2	278
3909	LAMA3	240
3910	LAMA4	54
3912	LAMB1	109
3913	LAMB2	296
3914	LAMB3	239
3918	LAMC2	224
392255	GDF6	285
3932	LCK	49
3952	LEP	161
3953	LEPR	164
3955	LFNG	215
3972	LHB	74
3973	LHCGR	85
3977	LIFR	372
3984	LIMK1	734
3991	LIPE	117
4068	SH2D1A	111
4088	SMAD3	400
4089	SMAD4	707
4091	SMAD6	77
4092	SMAD7	15
4093	SMAD9	21
4149	MAX	242
4157	MC1R	149
4193	MDM2	70
4214	MAP3K1	202
4233	MET	111
4254	KITLG	98
4261	CIITA	230
4286	MITF	305
4313	MMP2	250
4352	MPL	309
4609	MYC	93
4615	MYD88	235
462	SERPINC1	66

4633	MYL2	273
4638	MYLK	259
4688	NCF2	190
4689	NCF4	148
4747	NEFL	183
4763	NF1	383
4790	NFKB1	157
4792	NFKBIA	83
4838	NODAL	213
4846	NOS3	89
4851	NOTCH1	331
4853	NOTCH2	537
4854	NOTCH3	530
487	ATP2A1	6
488	ATP2A2	87
4893	NRAS	743
4982	TNFRSF11B	177
5054	SERPINE1	22
5058	PAK1	118
50615	IL21R	67
5063	PAK3	262
5111	PCNA	105
51135	IRAK4	31
51206	GP6	46
5137	PDE1C	7
5139	PDE3A	45
51422	PRKAG2	130
5155	PDGFB	467
5156	PDGFRA	138
5159	PDGFRB	440
51744	CD244	20
5216	PFN1	99
5255	PHKA1	37
5256	PHKA2	39
5257	PHKB	35
5261	PHKG2	65
5265	SERPINA1	60
5290	PIK3CA	750
5293	PIK3CD	81
5295	PIK3R1	411
5296	PIK3R2	185
5327	PLAT	11
5328	PLAU	81
5332	PLCB4	182
5336	PLCG2	105
5337	PLD1	74
5340	PLG	218

5345	SERPINF2	73
5350	PLN	81
5371	PML	167
54205	CYCS	7
54361	WNT4	232
5443	POMC	119
54567	DLL4	293
5495	PPM1B	108
5500	PPP1CB	299
5506	PPP1R3A	22
5515	PPP2CA	153
5518	PPP2R1A	153
5519	PPP2R1B	18
5521	PPP2R2B	106
5530	PPP3CA	265
5566	PRKACA	132
5568	PRKACG	42
5573	PRKAR1A	794
5575	PRKAR1B	68
5580	PRKCD	362
5582	PRKCG	121
5583	PRKCH	9
55851	PSENEN	37
5591	PRKDC	153
5592	PRKG1	193
5594	MAPK1	320
5602	MAPK10	58
5604	MAP2K1	477
5605	MAP2K2	413
5618	PRLR	72
5624	PROC	89
5627	PROS1	87
5644	PRSS1	99
5645	PRSS2	86
5649	RELN	81
5663	PSEN1	332
5664	PSEN2	162
57105	CYSLTR2	71
5728	PTEN	911
57522	SRGAP1	18
57680	CHD8	66
5770	PTPN1	22
5781	PTPN11	571
5792	PTPRF	38
58	ACTA1	530
5818	NECTIN1	375
5836	PYGL	42

5837	PYGM	48
5879	RAC1	294
5880	RAC2	50
5894	RAF1	466
59	ACTA2	254
5906	RAP1A	296
59067	IL21	52
5908	RAP1B	296
5914	RARA	178
5921	RASA1	65
5922	RASA2	267
5925	RB1	277
595	CCND1	257
596	BCL2	66
5970	RELA	84
5993	RFX5	216
5994	RFXAP	216
60	ACTB	520
6091	ROBO1	92
6092	ROBO2	17
613	BCR	378
6197	RPS6KA3	578
6237	RRAS	267
64218	SEMA4A	339
64221	ROBO3	66
6424	SFRP4	94
64321	SOX17	25
64840	PORCN	541
650	BMP2	482
652	BMP4	442
653361	NCF1	190
657	BMPR1A	435
658	BMPR1B	387
659	BMPR2	71
6647	SOD1	123
6654	SOS1	337
6655	SOS2	299
6696	SPP1	186
6714	SRC	86
673	BRAF	912
6772	STAT1	249
6773	STAT2	9
6774	STAT3	479
6775	STAT4	418
6777	STAT5B	187
6778	STAT6	128
6789	STK4	48

6868	ADAM17	75
6870	TACR3	276
6885	MAP3K7	532
6915	TBXA2R	30
6934	TCF7L2	22
695	BTK	253
70	ACTC1	80
7039	TGFA	30
7040	TGFB1	429
7042	TGFB2	264
7043	TGFB3	346
7046	TGFBR1	375
7048	TGFBR2	587
7056	THBD	59
7097	TLR2	6
7098	TLR3	47
7099	TLR4	287
71	ACTG1	324
7124	TNF	36
7132	TNFRSF1A	217
7133	TNFRSF1B	139
7148	TNXB	163
7157	TP53	510
7173	TPO	91
7189	TRAF6	66
72	ACTG2	242
7201	TRHR	76
7248	TSC1	401
7249	TSC2	385
7252	TSHB	104
7253	TSHR	179
7297	TYK2	33
7299	TYR	140
7305	TYROBP	190
7424	VEGFC	94
7450	VWF	96
7454	WAS	317
7471	WNT1	78
7473	WNT3	236
7474	WNT5A	467
7476	WNT7A	402
7480	WNT10B	117
7482	WNT2B	7
7535	ZAP70	194
7852	CXCR4	193
801	CALM1	64
80326	WNT10A	207

805	CALM2	81
8074	FGF23	82
808	CALM3	19
815	CAMK2A	66
816	CAMK2B	66
81607	NECTIN4	104
81624	DIAPH3	26
8200	GDF5	602
8302	KLRC4	287
8322	FZD4	84
8323	FZD6	10
83700	JAM3	86
8398	PLA2G6	304
841	CASP8	100
843	CASP10	331
84433	CARD11	77
8516	ITGA8	149
8517	IKBKG	437
85366	MYLK2	28
857	CAV1	497
859	CAV3	180
8600	TNFSF11	267
861	RUNX1	104
8625	RFXANK	216
8660	IRS2	22
867	CBL	183
8737	RIPK1	59
8772	FADD	56
8792	TNFRSF11A	288
8795	TNFRSF10B	9
8822	FGF17	286
8823	FGF16	28
8863	PER3	12
8864	PER2	13
8915	BCL10	163
894	CCND2	115
8945	BTRC	45
89780	WNT3A	29
90	ACVR1	199
90993	CREB3L1	67
9180	OSMR	38
9181	ARHGEF2	119
919	CD247	107
9241	NOG	372
925	CD8A	38
93	ACVR2B	32
930	CD19	194

94	ACVRL1	258
940	CD28	139
9423	NTN1	74
9451	EIF2AK3	393
9459	ARHGEF6	210
948	CD36	18
958	CD40	53
959	CD40LG	106
9630	GNA14	80
9672	SDC3	20
9863	MAGI2	37
990	CDC6	292
998	CDC42	308

Table S3. KEGG functional importance genes

Entrez Gene ID	Gene name	Drug-target (1: True, 0: False)	Drug ID (-: un-known)	Tumor suppres-sor (1: True, 0: False)	Oncogene (1: True, 0: False)
387	RHOA	1	DB04315	1	1
2885	GRB2	1	DB00061	0	0
2549	GAB1	0	-	0	1
25	ABL1	1	DB00171	0	1
6093	ROCK1	1	DB04707	0	1
9475	ROCK2	1	DB08162	0	0
1398	CRK	0	-	0	1
1399	CRKL	0	-	0	1
5879	RAC1	1	DB00993	0	1
5880	RAC2	1	DB00514	0	0
5881	RAC3	0	-	0	1
6714	SRC	1	DB01254	0	1
3667	IRS1	1	DB08513	0	0
8471	IRS4	0	-	0	0
8660	IRS2	0	-	0	1
7294	TXK	1	DB12010	0	0
5499	PPP1CA	1	DB02506	1	0
5500	PPP1CB	0	-	0	0
5501	PPP1CC	1	DB02169	0	0
2911	GRM1	1	DB00142	0	1
2915	GRM5	1	DB00659	0	0
2932	GSK3B	1	DB01356	1	0
5058	PAK1	1	DB12010	0	1
2776	GNAQ	0	-	0	1
1956	EGFR	1	DB00002	0	1
9020	MAP3K14	0	-	0	0
836	CASP3	1	DB01017	0	0
2889	RAPGEF1	0	-	0	0
2775	GNAO1	0	-	0	0
867	CBL	0	-	1	1
868	CBLB	0	-	0	1
23624	CBLC	0	-	0	0
2149	F2R	1	DB00086	0	0
998	CDC42	1	DB02623	0	0
5601	MAPK9	1	DB07020	1	0
5599	MAPK8	1	DB01782	0	0
5602	MAPK10	1	DB01782	1	0
4659	PPP1R12A	0	-	0	0
5335	PLCG1	0	-	0	0

Table S4. KEGG functional importance genes (continuous). *NuCancer* represents the number of KEGG genes associated with any cancer types.

Entrez Gene ID	*Gene name*	*Essential gene (1:True, 0:False)*	*NuCancer*	*Disease gene (1:True, 0:False)*
387	RHOA	1	16	1
2885	GRB2	1	0	1
2549	GAB1	1	18	1
25	ABL1	1	19	1
6093	ROCK1	1	0	1
9475	ROCK2	1	0	1
1398	CRK	1	0	1
1399	CRKL	1	11	1
5879	RAC1	1	12	1
5880	RAC2	0	16	1
5881	RAC3	1	0	1
6714	SRC	1	15	1
3667	IRS1	1	21	1
8471	IRS4	1	21	1
8660	IRS2	1	17	1
7294	TXK	0	0	1
5499	PPP1CA	1	0	1
5500	PPP1CB	1	11	1
5501	PPP1CC	1	0	1
2911	GRM1	1	0	1
2915	GRM5	1	0	1
2932	GSK3B	1	17	1
5058	PAK1	0	17	1
2776	GNAQ	1	15	1
1956	EGFR	1	21	1
9020	MAP3K14	0	6	1
836	CASP3	1	0	1
2889	RAPGEF1	1	0	1
2775	GNAO1	1	0	1
867	CBL	1	19	1
868	CBLB	1	20	1
23624	CBLC	0	20	1
2149	F2R	1	0	1
998	CDC42	1	16	1
5601	MAPK9	1	0	1
5599	MAPK8	1	0	1
5602	MAPK10	0	0	1
4659	PPP1R12A	1	0	1
5335	PLCG1	1	23	1
2534	FYN	1	18	1
4067	LYN	1	18	1
815	CAMK2A	1	0	1
816	CAMK2B	0	0	1
817	CAMK2D	0	0	1
818	CAMK2G	1	0	1
5900	RALGDS	0	18	1
5502	PPP1R1A	0	0	1
5781	PTPN11	1	17	1
1630	DCC	1	0	1
5336	PLCG2	1	20	1
23365	ARHGEF12	1	20	1

7409	VAV1	1	20	1
7410	VAV2	0	21	1
10451	VAV3	0	0	1
2768	GNA12	1	15	1
5747	PTK2	1	0	1
1855	DVL1	1	0	1
1856	DVL2	1	0	1
1857	DVL3	1	0	1
3265	HRAS	0	13	1
3845	KRAS	1	25	1
4893	NRAS	1	22	1
7048	TGFBR2	1	21	1
5290	PIK3CA	1	23	1
5291	PIK3CB	1	22	1
5293	PIK3CD	1	21	1
5294	PIK3CG	0	20	1
5906	RAP1A	1	0	1
5908	RAP1B	1	0	1
1499	CTNNB1	1	22	1
5295	PIK3R1	1	21	1
5296	PIK3R2	1	20	1
8503	PIK3R3	0	19	1
23533	PIK3R5	0	0	1
2147	F2	1	0	1
3688	ITGB1	1	0	1
9138	ARHGEF1	1	0	1
8772	FADD	1	0	1
10672	GNA13	1	15	1
27040	LAT	1	0	1
2475	MTOR	1	23	1
1445	CSK	1	0	1
1147	CHUK	1	0	1
3551	IKBKB	1	18	1
8517	IKBKG	1	0	1
3937	LCP2	1	0	1
5590	PRKCZ	1	0	1
5777	PTPN6	1	16	1
5579	PRKCB	1	0	1
3480	IGF1R	1	21	1
5340	PLG	1	0	1
8826	IQGAP1	1	0	1
5605	MAP2K2	0	16	1
6654	SOS1	1	22	1
6655	SOS2	1	0	1
9846	GAB2	1	16	1
2890	GRIA1	1	0	1
2891	GRIA2	1	0	1
5578	PRKCA	1	0	1
5582	PRKCG	1	□	1
6009	RHEB	1	12	1
5922	RASA2	0	0	1
7535	ZAP70	0	0	1
2770	GNAI1	1	0	1
2771	GNAI2	1	0	1
2773	GNAI3	0	0	1

9459	ARHGEF6	1	0	1
841	CASP8	1	19	1
6850	SYK	1	20	1
843	CASP10	0	0	1
5594	MAPK1	1	18	1
5595	MAPK3	1	13	1
25791	NGEF	1	0	1
2774	GNAL	1	0	1
55970	GNG12	1	0	0
7249	TSC2	1	22	1
3683	ITGAL	0	0	1
3689	ITGB2	1	0	1
1500	CTNND1	1	0	1
801	CALM1	0	0	1
805	CALM2	1	0	1
808	CALM3	0	0	1
810	CALML3	0	0	1
51806	CALML5	0	0	1
163688	CALML6	1	0	0
8936	WASF1	1	0	1
10163	WASF2	1	0	1
2159	F10	1	0	1
2207	FCER1G	1	0	1
2066	ERBB4	1	25	1
51744	CD244	0	0	1
3690	ITGB3	1	0	1
3691	ITGB4	1	0	1
3693	ITGB5	1	0	1
3694	ITGB6	0	0	1
3695	ITGB7	0	0	1
3696	ITGB8	1	0	1
5159	PDGFRB	1	22	1
2778	GNAS	1	22	1
7186	TRAF2	1	18	1
8737	RIPK1	1	0	1
1387	CREBBP	1	24	1
2033	EP300	1	27	1
7040	TGFB1	1	0	1
7042	TGFB2	1	0	1
7043	TGFB3	1	0	1
5156	PDGFRA	1	21	1
8440	NCK2	1	0	1
5921	RASA1	1	22	1
5062	PAK2	1	0	1
4690	NCK1	1	0	1
6464	SHC1	1	0	1
25759	SHC2	0	0	1
53358	SHC3	1	0	1
399694	SHC4	1	0	1
5515	PPP2CA	1	0	1
5516	PPP2CB	0	0	1
5518	PPP2R1A	1	18	1
5519	PPP2R1B	0	0	1
842	CASP9	1	0	1
6237	RRAS	0	10	1

22800	RRAS2	1	12	1
22808	MRAS	0	0	1
4615	MYD88	0	13	1
7157	TP53	1	26	1
1839	HBEGF	1	0	1
1729	DIAPH1	1	0	1
1730	DIAPH2	1	0	1
23396	PIP5K1C	1	0	1
3676	ITGA4	1	0	1
5170	PDPK1	1	15	1
4638	MYLK	0	0	1
85366	MYLK2	1	0	1
91807	MYLK3	0	0	1
5063	PAK3	1	19	1
10298	PAK4	1	0	1
56924	PAK6	1	0	1
57144	PAK5	0	21	1
27289	RND1	1	0	1
148022	TICAM1	0	0	1
3791	KDR	1	23	1
369	ARAF	1	19	1
4851	NOTCH1	1	23	1
4853	NOTCH2	1	20	1
4854	NOTCH3	0	23	1
4855	NOTCH4	0	23	1
5728	PTEN	1	21	1
10842	PPP1R17	0	0	1
4233	MET	1	20	1
5604	MAP2K1	1	16	1
1793	DOCK1	1	0	1
10928	RALBP1	0	0	1
5530	PPP3CA	1	0	1
5532	PPP3CB	1	0	1
5533	PPP3CC	0	0	1
5534	PPP3R1	1	0	1
5535	PPP3R2	0	0	0
11261	CHP1	1	0	1
63928	CHP2	0	0	1
6091	ROBO1	1	22	1
940	CD28	0	13	1
50649	ARHGEF4	1	0	1
5364	PLXNB1	0	0	1
5365	PLXNB3	0	0	1
23654	PLXNB2	1	0	1
9855	FARP2	0	0	1
7132	TNFRSF1A	1	0	1
7305	TYROBP	0	0	1
10746	MAP3K2	1	0	1
10458	BAIAP2	1	0	1
3684	ITGAM	0	0	1
9901	SRGAP3	1	0	1
23380	SRGAP2	0	0	1
57522	SRGAP1	1	0	1
3643	INSR	1	20	1
5898	RALA	1	0	1

5899	RALB	0	0	1
3984	LIMK1	1	0	1
3985	LIMK2	1	0	1
572	BAD	1	0	1
8717	TRADD	0	0	1
3383	ICAM1	0	0	1
5609	MAP2K7	1	0	1
5894	RAF1	1	19	1
4193	MDM2	1	19	1
5624	PROC	1	0	1
5361	PLXNA1	1	0	1
5362	PLXNA2	1	0	1
55558	PLXNA3	1	0	1
2260	FGFR1	1	19	1
4089	SMAD4	1	17	1
595	CCND1	1	14	1
894	CCND2	1	13	1
896	CCND3	1	14	1
2158	F9	1	0	1
3554	IL1R1	0	0	1
3680	ITGA9	1	0	1
355	FAS	1	15	1
3479	IGF1	1	15	1
2161	F12	0	0	1
3655	ITGA6	1	0	1
3672	ITGA1	1	0	1
3673	ITGA2	1	0	1
3674	ITGA2B	1	0	1
3675	ITGA3	1	0	1
3678	ITGA5	1	0	1
3679	ITGA7	1	0	1
3685	ITGAV	1	0	1
8515	ITGA10	0	0	1
8516	ITGA8	1	0	1
22801	ITGA11	0	0	1
2322	FLT3	0	20	1
6092	ROBO2	1	0	1
29851	ICOS	0	0	1
3815	KIT	1	23	1
7852	CXCR4	1	17	1
1910	EDNRB	1	0	1
1394	CRHR1	1	0	1
2065	ERBB3	1	21	1
2263	FGFR2	1	21	1
2064	ERBB2	1	22	1
7850	IL1R2	0	0	1
4301	AFDN	1	24	1
3932	LCK	1	17	1
3802	KIR2DL1	0	0	1
3803	KIR2DL2	0	0	1
3804	KIR2DL3	1	0	1
3805	KIR2DL4	0	0	1
3811	KIR3DL1	0	0	1
3812	KIR3DL2	0	0	1
3821	KLRC1	0	0	1

3824	KLRD1	0	0	1
57292	KIR2DL5A	0	0	1
2767	GNA11	1	17	1
2769	GNA15	0	0	1
9630	GNA14	0	0	1
2781	GNAZ	1	0	1
1020	CDK5	1	0	1
919	CD247	1	0	1
4214	MAP3K1	1	20	1
4052	LTBP1	1	0	1
4087	SMAD2	1	15	1
4088	SMAD3	1	18	1
5328	PLAU	1	0	1
7454	WAS	1	13	1
8976	WASL	1	0	1
5327	PLAT	1	0	1
1978	EIF4EBP1	0	0	1
394	ARHGAP5	1	0	1
2909	ARHGAP35	1	23	1
9564	BCAR1	1	0	1
353376	TICAM2	0	0	1
2535	FZD2	1	0	1
7855	FZD5	1	0	1
7976	FZD3	1	0	1
8321	FZD1	0	0	1
8322	FZD4	1	0	1
8323	FZD6	1	0	1
8324	FZD7	0	0	1
8325	FZD8	0	0	1
8326	FZD9	1	0	1
11211	FZD10	0	0	1
814	CAMK4	1	0	1
5566	PRKACA	1	17	1
5567	PRKACB	1	0	1
5568	PRKACG	0	0	1
5613	PRKX	0	0	1
5616	PRKY	0	0	1
1969	EPHA2	0	0	1
2041	EPHA1	0	0	1
2042	EPHA3	1	22	1
2043	EPHA4	1	0	1
2044	EPHA5	0	22	1
2045	EPHA7	1	22	1
2046	EPHA8	0	0	1
285220	EPHA6	1	0	1
2798	GNRHR	0	0	1
56288	PARD3	1	0	1
5255	PHKA1	0	0	1
5256	PHKA2	1	0	1
5257	PHKB	0	0	1
5260	PHKG1	0	0	1
5261	PHKG2	0	0	1
2245	FGD1	1	0	1
89846	FGD3	0	0	1
4068	SH2D1A	0	13	1

28964	GIT1	1	0	1
83593	RASSF5	0	0	1
10810	WASF3	1	0	1
3973	LHCGR	1	0	1
3624	INHBA	1	21	1
3625	INHBB	1	0	1
3626	INHBC	0	0	1
83729	INHBE	0	0	1
3071	NCKAP1L	1	0	1
10152	ABI2	1	0	1
10787	NCKAP1	1	0	1
23191	CYFIP1	1	0	1
26999	CYFIP2	1	0	1
8793	TNFRSF10D	0	0	1
8794	TNFRSF10C	0	0	1
8795	TNFRSF10B	0	0	1
8797	TNFRSF10A	0	0	1
207	AKT1	1	16	1
208	AKT2	1	19	1
10000	AKT3	1	18	1
2185	PTK2B	1	0	1
7133	TNFRSF1B	1	0	1
3556	IL1RAP	0	0	1
7056	THBD	1	0	1
2321	FLT1	1	21	1
2324	FLT4	1	22	1
695	BTK	1	18	1
1027	CDKN1B	1	14	1
1385	CREB1	1	13	1
3645	INSRR	1	0	1
468	ATF4	1	0	1
9586	CREB5	1	0	1
10488	CREB3	1	0	1
64764	CREB3L2	1	18	1
84699	CREB3L3	0	0	1
90993	CREB3L1	1	17	1
148327	CREB3L4	1	0	1
5054	SERPINE1	0	0	1
81624	DIAPH3	1	0	1
4157	MC1R	0	0	1
1947	EFNB1	1	0	1
1948	EFNB2	1	0	1
1949	EFNB3	0	0	1
1026	CDKN1A	1	17	1
4633	MYL2	1	0	1
4636	MYL5	1	0	0
10398	MYL9	0	0	1
10627	MYL12A	1	0	1
29895	MYLPF	1	0	1
58498	MYL7	1	0	1
93408	MYL10	0	0	0
103910	MYL12B	0	0	1
7189	TRAF6	1	0	1
7099	TLR4	0	0	1
5350	PLN	1	0	1

1616	DAXX	1	20	1
346562	GNAT3	0	0	1
2261	FGFR3	1	20	1
5111	PCNA	1	0	1
5584	PRKCI	1	17	1
1017	CDK2	1	0	1
55845	BRK1	1	0	1
2264	FGFR4	1	20	1
1028	CDKN1C	1	0	1
91	ACVR1B	1	18	1
983	CDK1	1	0	1
7187	TRAF3	1	17	1
890	CCNA2	1	0	1
8900	CCNA1	1	0	1
7471	WNT1	1	0	1
7472	WNT2	1	0	1
7473	WNT3	1	0	1
7474	WNT5A	1	0	1
7475	WNT6	1	0	1
7476	WNT7A	1	0	1
7477	WNT7B	1	0	1
7478	WNT8A	0	0	1
7479	WNT8B	0	0	1
7480	WNT10B	0	0	1
7481	WNT11	1	0	1
7482	WNT2B	0	0	1
7483	WNT9A	1	0	1
7484	WNT9B	1	0	1
51384	WNT16	0	0	1
54361	WNT4	1	0	1
80326	WNT10A	0	0	1
81029	WNT5B	0	0	1
89780	WNT3A	1	0	1
840	CASP7	1	0	1
3654	IRAK1	1	0	1
1019	CDK4	1	13	1
1021	CDK6	1	11	1
4216	MAP3K4	1	0	1
1030	CDKN2B	0	10	1
7046	TGFBR1	1	17	1
130399	ACVR1C	0	0	1
2241	FER	1	0	1
1487	CTBP1	1	0	1
1488	CTBP2	1	0	1
650	BMP2	1	0	1
4838	NODAL	1	0	1
51135	IRAK4	1	0	1
8554	PIAS1	1	0	1
9063	PIAS2	1	0	1
9641	IKBKE	1	18	1
10401	PIAS3	1	0	1
29110	TBK1	1	0	1
51588	PIAS4	1	0	1
655	BMP7	1	0	1
8200	GDF5	1	0	1

898	CCNE1	1	16	1
9134	CCNE2	1	0	1
596	BCL2	1	11	1
9088	PKMYT1	1	0	1
10092	ARPC5	0	0	1
81873	ARPC5L	0	0	0
268	AMH	1	0	1
959	CD40LG	1	0	1
4086	SMAD1	1	0	1
4090	SMAD5	1	0	1
4093	SMAD9	0	0	1
5627	PROS1	1	0	1
114609	TIRAP	0	0	1
941	CD80	0	0	1
3065	HDAC1	1	16	1
3066	HDAC2	1	0	1
3818	KLKB1	0	0	1
3827	KNG1	0	0	1
3384	ICAM2	0	0	1
3385	ICAM3	0	0	1
652	BMP4	1	0	1
653	BMP5	1	0	1
654	BMP6	0	0	1
656	BMP8B	1	0	1
151449	GDF7	0	0	1
353500	BMP8A	1	0	1
392255	GDF6	1	0	1
4991	OR1D2	0	0	0
4992	OR1F1	0	0	0
4993	OR2C1	0	0	0
4994	OR3A1	0	0	0
4995	OR3A2	0	0	1
7932	OR2H2	0	0	0
8383	OR1A1	0	0	0
8385	-	1	0	0
8386	OR1D5	0	0	0
8387	OR1E1	0	0	0
8388	OR1E2	0	0	1
8390	OR1G1	0	0	0
8392	OR3A3	0	0	0
8590	OR6A2	0	0	0
10798	OR5I1	0	0	0
23538	OR52A1	0	0	0
26188	OR1C1	0	0	0
26189	OR1A2	0	0	0
26211	OR2F1	0	0	0
26212	OR2B6	0	0	0
26219	OR1J4	0	0	0
26245	OR2M4	0	0	0
26246	OR2L2	0	0	0
26248	OR2K2	0	0	0
26333	OR7A17	0	0	0
26338	OR5L2	0	0	0
26339	OR5K1	0	0	0
26476	OR10J1	0	0	1

26492	OR8G2P	0	0	0
26493	OR8B8	0	0	0
26494	OR8G1	0	0	0
26496	OR10A3	0	0	0
26529	OR12D2	0	0	0
26531	OR11A1	0	0	0
26532	OR10H3	0	0	0
26533	OR10G3	0	0	0
26534	OR10G2	0	0	0
26538	OR10H2	0	0	0
26539	OR10H1	0	0	0
26658	OR7C2	0	0	0
26659	OR7A5	1	0	0
26664	OR7C1	0	0	0
26682	OR4F4	0	0	0
26683	OR4F3	0	0	0
26686	OR4E2	0	0	0
26689	OR4D1	0	0	0
26692	OR2W1	0	0	0
26696	OR2T1	0	0	0
26707	OR2J2	0	0	0
26716	OR2H1	0	0	0
26735	OR1L3	0	0	0
26737	OR1L1	0	0	0
26740	OR1J2	0	0	0
56656	OR2S2	1	0	0
79290	OR13A1	0	0	0
79295	OR5H6	0	0	0
79310	OR5H2	0	0	0
79317	OR4K5	0	0	0
79324	OR51G1	0	0	0
79339	OR51B4	0	0	0
79345	OR51B2	0	0	0
79473	OR52N1	0	0	0
79501	OR4F5	0	0	0
79541	OR2A4	0	0	0
79544	OR4K1	0	0	0
81050	OR5AC2	0	0	0
81061	OR11H1	0	0	0
81099	OR4F17	0	0	0
81127	OR4K15	0	0	0
81168	OR8J3	0	0	0
81282	OR51G2	0	0	0
81285	OR51E2	0	0	1
81300	OR4P4	0	0	0
81309	OR4C15	0	0	1
81318	OR4A5	0	0	0
81327	OR4A16	0	0	0
81328	OR4A15	0	0	0
81392	OR2AE1	0	0	0
81399	OR4F16	0	0	0
81442	OR6N2	0	0	0
81448	OR6K2	0	0	0
81469	OR2G3	0	0	0
81470	OR2G2	0	0	0

81472	OR2C3	0	0	0
81696	OR5V1	0	0	1
81697	OR2B2	0	0	0
81797	OR12D3	0	0	1
119678	OR52E2	0	0	0
119679	OR52J3	0	0	0
119682	OR51L1	0	0	0
119687	OR51A7	0	0	0
119692	OR51S1	0	0	0
119694	OR51F2	0	0	0
119695	OR52R1	0	0	0
119749	OR4C46	0	0	1
119764	OR4X2	0	0	0
119765	OR4B1	0	0	0
119772	OR52M1	0	0	0
119774	OR52K2	0	0	0
120065	OR5P2	0	0	0
120066	OR5P3	0	0	0
120586	OR8I2	1	0	0
120775	OR2D3	0	0	0
120776	OR2D2	0	0	0
120787	OR52W1	0	0	0
120793	OR56A4	0	0	0
120796	OR56A1	0	0	0
121130	OR10P1	0	0	0
121275	OR10AD1	0	0	0
121364	OR10A7	0	0	0
122740	OR4K14	0	0	0
122742	OR4L1	0	0	0
122748	OR11H6	0	0	0
124538	OR4D2	1	0	0
125958	OR7D4	0	0	1
125962	OR7G1	0	0	0
125963	OR1M1	0	0	0
126370	OR1I1	0	0	0
126541	OR10H4	0	0	0
127059	OR2M5	0	0	0
127062	OR2M3	0	0	0
127064	OR2T12	0	0	1
127066	OR14C36	0	0	0
127068	OR2T34	0	0	0
127069	OR2T10	0	0	0
127074	OR2T4	0	0	0
127077	OR2T11	0	0	0
127385	OR10J5	0	0	0
127623	OR2B11	0	0	0
128360	OR10T2	0	0	1
128367	OR10X1	0	0	0
128368	OR10Z1	0	0	1
128371	OR6K6	0	0	0
128372	OR6N1	0	0	1
130075	OR9A4	0	0	0
134083	OR2Y1	0	0	0
135924	OR9A2	0	0	0
135941	OR2A14	0	0	0

135946	OR6B1	0	0	0
135948	OR2F2	0	0	0
138799	OR13C5	0	0	0
138802	OR13C8	0	0	0
138803	OR13C3	0	0	0
138804	OR13C4	0	0	0
138805	OR13F1	0	0	0
138881	OR1L8	1	0	0
138882	OR1N2	0	0	0
138883	OR1N1	0	0	1
143496	OR52B4	0	0	0
143502	OR52I2	0	0	0
143503	OR51E1	0	0	1
144124	OR10A5	0	0	0
144125	OR2AG1	0	0	1
150681	OR6B3	0	0	0
158131	OR1Q1	0	0	0
162998	OR7D2	0	0	0
196335	OR56B4	0	0	0
219417	OR8U1	0	0	0
219428	OR4C16	0	0	0
219429	OR4C11	0	0	0
219431	OR4S2	0	0	0
219432	OR4C6	0	0	0
219436	OR5D14	0	0	0
219437	OR5L1	0	0	0
219438	OR5D18	1	0	0
219447	OR5AS1	0	0	0
219453	OR8K5	0	0	0
219464	OR5T2	0	0	0
219469	OR8H1	0	0	0
219473	OR8K3	0	0	0
219477	OR8J1	0	0	0
219479	OR5R1	0	0	0
219482	OR5M3	0	0	0
219484	OR5M8	0	0	0
219487	OR5M11	0	0	0
219493	OR5AR1	1	0	0
219858	OR8B12	0	0	0
219865	OR8G5	0	0	0
219869	OR10G8	0	0	0
219870	OR10G9	0	0	0
219873	OR10S1	0	0	0
219874	OR6T1	0	0	0
219875	OR4D5	0	0	0
219952	OR6Q1	0	0	0
219954	OR9I1	1	0	0
219956	OR9Q1	0	0	1
219957	OR9Q2	0	0	0
219958	OR1S2	0	0	0
219959	OR1S1	1	0	0
219960	OR10Q1	0	0	0
219965	OR5B17	0	0	0
219968	OR5B21	0	0	0
219981	OR5A2	0	0	0

219982	OR5A1	0	0	0
219983	OR4D6	0	0	0
219986	OR4D11	1	0	0
254783	OR6C74	0	0	0
254786	OR6C3	0	0	0
254879	OR2T6	1	0	0
254973	OR1L4	0	0	0
255725	OR52B2	0	0	0
256144	OR4C3	1	0	0
256148	OR4S1	0	0	0
256892	OR51F1	0	0	1
282763	OR51B5	0	0	1
282770	OR10AG1	0	0	0
282775	OR5J2	0	0	1
283092	OR4C13	0	0	1
283093	OR4C12	0	0	0
283111	OR51V1	0	0	0
283159	OR8D1	0	0	0
283160	OR8D2	0	0	0
283162	OR8B4	0	0	0
283189	OR9G4	0	0	0
283297	OR10A4	0	0	1
283365	OR6C6	0	0	0
283694	OR4N4	0	0	0
284383	OR2Z1	0	0	0
284433	OR10H5	0	0	0
284521	OR2L13	0	0	0
284532	OR14A16	0	0	0
285659	OR2V2	0	0	0
286362	OR13C9	0	0	0
286365	OR13D1	0	0	0
338662	OR8D4	0	0	0
338674	OR5F1	0	0	0
338675	OR5AP2	0	0	0
338751	OR52L1	0	0	0
338755	OR2AG2	0	0	0
340980	OR52B6	0	0	0
341152	OR2AT4	0	0	0
341276	OR10A2	0	0	0
341416	OR6C2	0	0	0
341418	OR6C4	0	0	0
341568	OR8S1	0	0	1
341799	OR6S1	0	0	0
343169	OR6F1	0	0	1
343171	OR2W3	0	0	0
343172	OR2T8	0	0	0
343173	OR2T3	0	0	0
343406	OR10R2	0	0	0
343563	OR2T29	0	0	0
346517	OR6V1	0	0	0
346525	OR2A12	0	0	0
346528	OR2A1	0	0	0
347168	OR1J1	1	0	0
347169	OR1B1	0	0	0
347468	OR13H1	0	0	0

387748	OR56B1	0	0	0
389090	OR6B2	0	0	0
390036	OR52K1	0	0	0
390037	OR52I1	0	0	0
390038	OR51D1	0	0	0
390054	OR52A5	0	0	0
390058	OR51B6	0	0	1
390059	OR51M1	0	0	0
390061	OR51Q1	0	0	0
390063	OR51I1	0	0	0
390064	OR51I2	0	0	0
390066	OR52D1	0	0	0
390067	OR52H1	0	0	0
390072	OR52N4	0	0	0
390075	OR52N5	0	0	0
390077	OR52N2	1	0	0
390078	OR52E6	0	0	0
390079	OR52E8	0	0	0
390081	OR52E4	0	0	0
390083	OR56A3	0	0	0
390084	OR56A5	0	0	0
390093	OR10A6	0	0	0
390113	OR4X1	0	0	1
390142	OR5D13	0	0	0
390144	OR5D16	0	0	0
390151	OR8H2	0	0	0
390152	OR8H3	0	0	0
390154	OR5T3	0	0	0
390155	OR5T1	0	0	0
390157	OR8K1	0	0	0
390162	OR5M9	0	0	0
390167	OR5M10	0	0	0
390168	OR5M1	0	0	1
390174	OR9G1	0	0	0
390181	OR5AK2	0	0	0
390191	OR5B12	0	0	0
390195	OR5AN1	0	0	0
390197	OR4D10	0	0	0
390199	OR4D9	0	0	0
390201	OR10V1	0	0	0
390260	OR6X1	0	0	0
390261	OR6M1	0	0	0
390264	OR10G4	0	0	0
390265	OR10G7	0	0	0
390275	OR8A1	0	0	0
390321	OR6C1	0	0	0
390323	OR6C75	0	0	0
390326	OR6C76	1	0	0
390327	OR6C70	0	0	0
390429	OR4N2	0	0	0
390431	OR4K2	0	0	0
390433	OR4K13	0	0	0
390436	OR4K17	0	0	0
390437	OR4N5	0	0	0
390439	OR11G2	0	0	0

390442	OR11H4	0	0	0
390445	OR5AU1	1	0	0
390538	OR4M2	0	0	0
390648	OR4F6	0	0	0
390649	OR4F15	0	0	0
390882	OR7G2	0	0	0
390883	OR7G3	1	0	0
390892	OR7A10	0	0	0
391107	OR10K2	0	0	0
391109	OR10K1	0	0	0
391112	OR6Y1	0	0	1
391114	OR6K3	0	0	0
391189	OR11L1	0	0	0
391190	OR2L8	0	0	0
391191	OR2AK2	0	0	0
391192	OR2L3	0	0	0
391194	OR2M2	0	0	1
391195	OR2T33	0	0	0
391196	OR2M7	0	0	1
391211	OR2G6	0	0	0
392138	OR2A25	0	0	0
392309	OR13J1	0	0	1
392376	OR13C2	0	0	0
392390	OR1L6	0	0	0
392391	OR5C1	0	0	0
392392	OR1K1	0	0	0
393046	OR2A5	0	0	0
401427	OR2A7	0	0	0
401665	OR51T1	0	0	0
401666	OR51A4	0	0	0
401667	OR51A2	0	0	0
401992	OR2T2	0	0	0
401993	OR2T5	0	0	0
401994	OR14I1	0	0	0
402135	OR5K2	0	0	0
402317	OR2A42	0	0	0
441608	OR5B3	0	0	0
441639	OR9K2	1	0	0
441669	OR4Q3	0	0	0
441670	OR4M1	0	0	0
441911	OR10J3	0	0	0
441933	OR13G1	0	0	1
442186	OR2J3	0	0	1
442191	OR14J1	0	0	1
442194	OR10C1	0	0	0
442361	OR2A2	0	0	0
504189	OR8U8	0	0	0
2	A2M	0	0	1
27	ABL2	1	17	1
31	ACACA	1	0	1
32	ACACB	0	0	1
52	ACP1	1	0	1
58	ACTA1	1	0	1
59	ACTA2	0	0	1
60	ACTB	1	20	1

70	ACTC1	1	0	1
71	ACTG1	1	18	1
72	ACTG2	1	0	1
90	ACVR1	1	15	1
92	ACVR2A	1	20	1
93	ACVR2B	1	0	1
94	ACVRL1	1	0	1
102	ADAM10	1	0	1
107	ADCY1	1	0	1
108	ADCY2	1	0	1
109	ADCY3	1	0	1
111	ADCY5	1	0	1
112	ADCY6	0	0	1
113	ADCY7	1	0	1
114	ADCY8	0	0	1
115	ADCY9	1	0	1
135	ADORA2A	0	0	1
136	ADORA2B	0	0	1
146	ADRA1D	0	0	1
147	ADRA1B	0	0	1
148	ADRA1A	0	0	1
153	ADRB1	1	0	1
154	ADRB2	0	0	1
155	ADRB3	0	0	1
157	GRK3	0	0	1
182	JAG1	1	0	1
185	AGTR1	1	0	1
269	AMHR2	1	0	1
317	APAF1	1	0	1
324	APC	1	25	1
329	BIRC2	1	0	1
330	BIRC3	1	14	1
331	XIAP	1	14	1
356	FASLG	1	0	1
374	AREG	1	0	1
406	ARNTL	1	0	1
409	ARRB2	0	0	1
434	ASIP	1	0	1
462	SERPINC1	1	0	1
487	ATP2A1	1	0	1
488	ATP2A2	1	0	1
489	ATP2A3	0	0	1
552	AVPR1A	0	0	1
553	AVPR1B	0	0	1
598	BCL2L1	1	12	1
613	BCR	1	21	1
623	BDKRB1	0	0	1
624	BDKRB2	1	0	1
637	BID	1	0	1
657	BMPR1A	1	17	1
658	BMPR1B	1	0	1
659	BMPR2	1	0	1
673	BRAF	1	22	1
685	BTC	0	0	1
839	CASP6	0	0	1

857	CAV1	0	0	1
858	CAV2	0	0	1
859	CAV3	0	0	1
861	RUNX1	1	18	1
862	RUNX1T1	1	20	1
886	CCKAR	0	0	1
887	CCKBR	0	0	1
891	CCNB1	1	0	1
925	CD8A	1	0	1
926	CD8B	0	0	1
929	CD14	0	0	1
930	CD19	1	0	1
942	CD86	1	0	1
948	CD36	0	16	1
958	CD40	0	0	1
960	CD44	0	0	1
961	CD47	0	0	1
962	CD48	0	0	1
990	CDC6	1	0	1
1022	CDK7	1	0	1
1029	CDKN2A	1	20	1
1031	CDKN2C	1	12	1
1032	CDKN2D	1	0	1
1050	CEBPA	1	9	1
1072	CFL1	1	0	1
1073	CFL2	0	0	1
1080	CFTR	1	0	1
1081	CGA	1	0	1
1101	CHAD	1	0	1
1128	CHRM1	1	0	1
1129	CHRM2	0	0	1
1131	CHRM3	1	0	1
1132	CHRM4	1	0	1
1133	CHRM5	0	0	1
1154	CISH	1	0	1
1270	CNTF	1	0	1
1271	CNTFR	1	0	1
1277	COL1A1	1	21	1
1278	COL1A2	1	0	1
1280	COL2A1	1	22	1
1281	COL3A1	1	0	1
1282	COL4A1	1	0	1
1284	COL4A2	1	0	1
1286	COL4A4	0	0	1
1288	COL4A6	1	0	1
1289	COL5A1	1	0	1
1290	COL5A2	1	0	1
1291	COL6A1	1	0	1
1292	COL6A2	0	0	1
1293	COL6A3	0	0	1
1301	COL11A1	1	0	1
1302	COL11A2	1	0	1
1311	COMP	0	0	1
1392	CRH	1	0	1
1407	CRY1	0	0	1

1408	CRY2	1	0	1
1432	MAPK14	1	0	1
1437	CSF2	1	0	1
1438	CSF2RA	0	0	1
1439	CSF2RB	1	0	1
1440	CSF3	0	0	1
1441	CSF3R	1	21	1
1442	CSH1	0	0	1
1452	CSNK1A1	1	0	1
1453	CSNK1D	1	0	1
1454	CSNK1E	1	0	1
1455	CSNK1G2	1	0	1
1456	CSNK1G3	1	0	1
1457	CSNK2A1	1	0	1
1459	CSNK2A2	1	0	1
1460	CSNK2B	1	0	1
1489	CTF1	1	0	1
1493	CTLA4	1	15	1
1511	CTSG	0	0	1
1535	CYBA	0	0	1
1536	CYBB	1	0	1
1605	DAG1	1	0	1
1634	DCN	1	0	1
1647	GADD45A	1	0	1
1676	DFFA	0	0	1
1808	DPYSL2	1	0	1
1812	DRD1	1	0	1
1813	DRD2	1	0	1
1816	DRD5	1	0	1
1840	DTX1	0	21	1
1843	DUSP1	0	0	1
1844	DUSP2	0	14	1
1845	DUSP3	0	0	1
1846	DUSP4	0	13	1
1847	DUSP5	0	0	1
1848	DUSP6	1	0	1
1849	DUSP7	0	0	1
1850	DUSP8	0	0	1
1852	DUSP9	1	14	1
1869	E2F1	1	0	1
1870	E2F2	1	0	1
1871	E2F3	1	19	1
1874	E2F4	1	0	1
1875	E2F5	1	0	1
1902	LPAR1	1	0	1
1906	EDN1	1	0	1
1909	EDNRA	1	0	1
1942	EFNA1	0	0	1
1943	EFNA2	1	0	1
1944	EFNA3	1	0	1
1945	EFNA4	0	0	1
1946	EFNA5	1	0	1
1950	EGF	1	0	1
1977	EIF4E	1	14	1
2047	EPHB1	1	22	1

2048	EPHB2	1	0	1
2049	EPHB3	1	0	1
2050	EPHB4	1	20	1
2051	EPHB6	0	0	1
2056	EPO	1	0	1
2057	EPOR	1	18	1
2069	EREG	0	0	1
2081	ERN1	1	0	1
2113	ETS1	1	19	1
2122	MECOM	1	22	1
2150	F2RL1	1	0	1
2151	F2RL2	0	0	1
2152	F3	1	0	1
2153	F5	1	0	1
2155	F7	1	0	1
2157	F8	1	0	1
2160	F11	1	0	1
2162	F13A1	1	0	1
2165	F13B	0	0	1
2206	MS4A2	0	0	1
2242	FES	1	17	1
2243	FGA	1	0	1
2244	FGB	0	0	1
2246	FGF1	0	0	1
2247	FGF2	0	0	1
2248	FGF3	1	16	1
2249	FGF4	1	11	1
2250	FGF5	0	0	1
2251	FGF6	0	18	1
2252	FGF7	0	0	1
2253	FGF8	1	0	1
2254	FGF9	1	0	1
2255	FGF10	1	14	1
2256	FGF11	0	0	1
2257	FGF12	0	16	1
2258	FGF13	1	0	1
2259	FGF14	0	16	1
2266	FGG	1	0	1
2277	VEGFD	0	0	1
2308	FOXO1	1	19	1
2309	FOXO3	1	18	1
2323	FLT3LG	1	0	1
2335	FN1	1	0	1
2353	FOS	1	0	1
2488	FSHB	1	0	1
2492	FSHR	1	0	1
2648	KAT2A	1	0	1
2688	GH1	0	0	1
2689	GH2	0	0	1
2690	GHR	1	0	1
2697	GJA1	1	0	1
2735	GLI1	1	22	1
2736	GLI2	1	0	1
2737	GLI3	1	0	1
2782	GNB1	1	21	1

2784	GNB3	1	0	1
2785	GNG3	1	0	1
2796	GNRH1	0	0	1
2797	GNRH2	0	0	1
2810	SFN	1	0	1
2811	GP1BA	0	0	1
2812	GP1BB	1	0	1
2814	GP5	0	0	1
2815	GP9	0	0	1
2872	MKNK2	0	0	1
2892	GRIA3	1	0	1
2925	GRPR	0	0	1
2934	GSN	1	0	1
2997	GYS1	1	0	1
2998	GYS2	0	0	1
3001	GZMA	0	0	1
3002	GZMB	0	0	1
3053	SERPIND1	1	0	1
3082	HGF	1	19	1
3084	NRG1	1	17	1
3105	HLA-A	0	15	1
3106	HLA-B	0	16	1
3107	HLA-C	0	12	1
3133	HLA-E	0	0	1
3134	HLA-F	0	0	1
3135	HLA-G	0	0	1
3269	HRH1	0	0	1
3274	HRH2	0	0	1
3303	HSPA1A	0	0	1
3304	HSPA1B	0	0	1
3305	HSPA1L	0	0	1
3306	HSPA2	1	0	1
3310	HSPA6	0	0	1
3312	HSPA8	1	0	1
3339	HSPG2	1	0	1
3356	HTR2A	0	0	1
3357	HTR2B	1	0	1
3358	HTR2C	1	0	1
3360	HTR4	1	0	1
3361	HTR5A	0	0	1
3362	HTR6	0	0	1
3363	HTR7	0	0	1
3371	TNC	0	0	1
3381	IBSP	0	0	1
3439	IFNA1	0	0	1
3440	IFNA2	0	0	1
3441	IFNA4	0	0	1
3442	IFNA5	0	0	1
3443	IFNA6	0	0	1
3444	IFNA7	0	0	1
3445	IFNA8	0	0	1
3446	IFNA10	0	0	1
3447	IFNA13	0	0	1
3448	IFNA14	0	0	1
3449	IFNA16	0	0	0

3451	IFNA17	0	0	1
3452	IFNA21	0	0	0
3454	IFNAR1	0	0	1
3455	IFNAR2	0	0	1
3456	IFNB1	0	0	1
3458	IFNG	1	0	1
3459	IFNGR1	0	18	1
3460	IFNGR2	0	0	1
3467	IFNW1	1	0	0
3486	IGFBP3	1	0	1
3516	RBPJ	1	0	1
3552	IL1A	1	0	1
3553	IL1B	1	0	1
3558	IL2	0	8	1
3559	IL2RA	1	0	1
3560	IL2RB	1	0	1
3561	IL2RG	1	0	1
3562	IL3	1	10	1
3563	IL3RA	0	0	1
3565	IL4	1	0	1
3566	IL4R	1	0	1
3567	IL5	1	0	1
3568	IL5RA	0	0	1
3569	IL6	0	0	1
3570	IL6R	0	0	1
3572	IL6ST	1	18	1
3574	IL7	0	0	1
3575	IL7R	0	16	1
3578	IL9	1	0	1
3581	IL9R	1	0	1
3586	IL10	0	13	1
3587	IL10RA	1	0	1
3588	IL10RB	0	0	1
3589	IL11	0	0	1
3590	IL11RA	1	0	1
3592	IL12A	0	0	1
3593	IL12B	0	0	1
3594	IL12RB1	1	0	1
3595	IL12RB2	0	0	1
3596	IL13	0	0	1
3597	IL13RA1	1	0	1
3598	IL13RA2	0	0	1
3600	IL15	1	0	1
3601	IL15RA	0	0	1
3630	INS	1	0	1
3656	IRAK2	0	0	1
3661	IRF3	0	0	1
3663	IRF5	0	0	1
3665	IRF7	0	0	1
3681	ITGAD	0	0	1
3682	ITGAE	0	0	1
3687	ITGAX	0	0	1
3702	ITK	0	20	1
3706	ITPKA	0	0	1
3707	ITPKB	1	0	1

3714	JAG2	1	0	1
3716	JAK1	1	21	1
3717	JAK2	1	21	1
3718	JAK3	1	21	1
3725	JUN	1	16	1
3745	KCNB1	1	0	1
3784	KCNQ1	0	0	1
3806	KIR2DS1	0	0	1
3808	KIR2DS3	0	0	1
3809	KIR2DS4	0	0	1
3810	KIR2DS5	0	0	1
3822	KLRC2	1	0	1
3823	KLRC3	0	0	1
3908	LAMA2	1	0	1
3909	LAMA3	1	0	1
3910	LAMA4	1	0	1
3911	LAMA5	1	0	1
3912	LAMB1	1	0	1
3913	LAMB2	1	0	1
3914	LAMB3	1	0	1
3915	LAMC1	1	0	1
3918	LAMC2	1	0	1
3952	LEP	1	0	1
3953	LEPR	1	0	1
3955	LFNG	1	0	1
3972	LHB	1	0	1
3976	LIF	1	0	1
3977	LIFR	1	19	1
3983	ABLIM1	1	0	1
3991	LIPE	1	0	1
4049	LTA	0	0	1
4050	LTB	0	9	1
4055	LTBR	0	0	1
4091	SMAD6	1	0	1
4092	SMAD7	1	0	1
4149	MAX	1	14	1
4194	MDM4	1	13	1
4215	MAP3K3	1	0	1
4217	MAP3K5	1	0	1
4242	MFNG	0	0	1
4254	KITLG	1	0	1
4261	CIITA	0	24	1
4286	MITF	1	17	1
4313	MMP2	1	0	1
4342	MOS	0	0	1
4352	MPL	1	17	1
4609	MYC	1	17	1
4616	GADD45B	1	3	1
4688	NCF2	0	0	1
4689	NCF4	1	0	1
4747	NEFL	0	0	1
4763	NF1	1	26	1
4772	NFATC1	1	0	1
4773	NFATC2	1	20	1
4775	NFATC3	1	0	1

4776	NFATC4	1	0	1
4790	NFKB1	1	0	1
4792	NFKBIA	1	18	1
4800	NFYA	1	0	1
4801	NFYB	1	0	1
4802	NFYC	1	0	1
4846	NOS3	1	0	1
4862	NPAS2	1	0	1
4923	NTSR1	1	0	1
4982	TNFRSF11B	1	0	1
5008	OSM	0	0	1
5021	OXTR	1	0	1
5104	SERPINA5	1	0	1
5132	PDC	0	0	1
5136	PDE1A	0	0	1
5137	PDE1C	0	0	1
5139	PDE3A	1	0	1
5140	PDE3B	0	0	1
5153	PDE1B	1	0	1
5154	PDGFA	1	0	1
5155	PDGFB	1	13	1
5187	PER1	0	23	1
5216	PFN1	1	0	1
5217	PFN2	0	0	1
5228	PGF	0	0	1
5265	SERPINA1	0	0	1
5305	PIP4K2A	1	0	1
5319	PLA2G1B	0	0	1
5320	PLA2G2A	0	0	1
5321	PLA2G4A	1	0	1
5322	PLA2G5	0	0	1
5329	PLAUR	1	0	1
5330	PLCB2	1	0	1
5331	PLCB3	1	0	1
5332	PLCB4	1	0	1
5337	PLD1	0	0	1
5345	SERPINF2	0	0	1
5347	PLK1	1	0	1
5371	PML	1	22	1
5443	POMC	1	0	1
5494	PPM1A	1	0	1
5495	PPM1B	1	0	1
5506	PPP1R3A	0	0	1
5507	PPP1R3C	1	0	1
5509	PPP1R3D	0	0	0
5520	PPP2R2A	1	22	1
5521	PPP2R2B	1	0	1
5522	PPP2R2C	0	0	1
5536	PPP5C	1	0	1
5562	PRKAA1	1	0	1
5563	PRKAA2	0	0	1
5564	PRKAB1	1	0	1
5565	PRKAB2	1	0	1
5571	PRKAG1	1	0	1
5573	PRKAR1A	1	15	1

5575	PRKAR1B	0	0	1
5576	PRKAR2A	1	0	1
5577	PRKAR2B	1	0	1
5580	PRKCD	1	0	1
5581	PRKCE	1	0	1
5583	PRKCH	1	0	1
5588	PRKCQ	1	0	1
5591	PRKDC	1	26	1
5592	PRKG1	1	0	1
5593	PRKG2	0	0	1
5598	MAPK7	1	0	1
5600	MAPK11	0	0	1
5603	MAPK13	0	0	1
5606	MAP2K3	1	0	1
5607	MAP2K5	1	0	1
5608	MAP2K6	1	0	1
5617	PRL	1	0	1
5618	PRLR	1	0	1
5644	PRSS1	0	19	1
5645	PRSS2	0	0	1
5646	PRSS3	0	0	1
5649	RELN	1	24	1
5663	PSEN1	1	0	1
5664	PSEN2	1	0	1
5720	PSME1	0	0	1
5721	PSME2	0	0	1
5724	PTAFR	0	0	1
5726	TAS2R38	0	0	1
5731	PTGER1	0	0	1
5733	PTGER3	0	0	1
5737	PTGFR	1	0	1
5770	PTPN1	1	14	1
5778	PTPN7	0	0	1
5787	PTPRB	1	21	1
5792	PTPRF	1	0	1
5795	PTPRJ	1	0	1
5797	PTPRM	1	0	1
5801	PTPRR	0	0	1
5817	PVR	0	0	1
5818	NECTIN1	0	0	1
5819	NECTIN2	1	0	1
5829	PXN	1	0	1
5834	PYGB	0	0	1
5836	PYGL	1	0	1
5837	PYGM	1	0	1
5871	MAP4K2	1	0	1
5914	RARA	1	19	1
5923	RASGRF1	1	0	1
5924	RASGRF2	1	0	1
5925	RB1	1	21	1
5933	RBL1	1	0	1
5934	RBL2	1	0	1
5970	RELA	1	0	1
5986	RFNG	1	0	1
5993	RFX5	1	0	1

5994	RFXAP	1	0	1
5998	RGS3	0	0	1
6195	RPS6KA1	0	0	1
6196	RPS6KA2	0	0	1
6197	RPS6KA3	1	0	1
6198	RPS6KB1	1	0	1
6199	RPS6KB2	0	18	1
6300	MAPK12	0	0	1
6382	SDC1	1	0	1
6383	SDC2	0	0	1
6385	SDC4	0	17	1
6387	CXCL12	1	0	1
6416	MAP2K4	1	17	1
6422	SFRP1	1	0	1
6423	SFRP2	1	0	1
6424	SFRP4	0	15	1
6425	SFRP5	1	0	1
6494	SIPA1	1	0	1
6500	SKP1	1	0	1
6558	SLC12A2	1	0	1
6585	SLIT1	1	0	1
6647	SOD1	1	0	1
6667	SP1	1	0	1
6688	SPI1	1	0	1
6696	SPP1	1	0	1
6772	STAT1	1	0	1
6773	STAT2	0	0	1
6774	STAT3	1	19	1
6775	STAT4	1	20	1
6776	STAT5A	1	20	1
6777	STAT5B	1	21	1
6778	STAT6	1	23	1
6788	STK3	0	0	1
6789	STK4	0	0	1
6865	TACR2	1	0	1
6868	ADAM17	1	0	1
6869	TACR1	0	0	1
6870	TACR3	0	0	1
6885	MAP3K7	1	18	1
6915	TBXA2R	1	0	1
6932	TCF7	1	0	1
6934	TCF7L2	1	20	1
7027	TFDP1	1	0	1
7035	TFPI	1	0	1
7039	TGFA	1	0	1
7044	LEFTY2	1	0	1
7057	THBS1	1	0	1
7058	THBS2	1	0	1
7059	THBS3	0	0	1
7060	THBS4	0	0	1
7070	THY1	0	0	1
7074	TIAM1	1	0	1
7097	TLR2	0	0	1
7098	TLR3	0	0	1
7100	TLR5	0	0	1

7124	TNF	1	0	1
7143	TNR	1	0	1
7148	TNXB	0	0	1
7173	TPO	0	0	1
7201	TRHR	0	0	1
7248	TSC1	1	21	1
7252	TSHB	0	0	1
7253	TSHR	1	20	1
7297	TYK2	0	23	1
7299	TYR	1	0	1
7412	VCAM1	1	0	1
7422	VEGFA	1	14	1
7423	VEGFB	0	0	1
7424	VEGFC	1	0	1
7448	VTN	0	0	1
7450	VWF	1	0	1
7465	WEE1	1	0	1
7525	YES1	1	19	1
8027	STAM	1	0	1
8074	FGF23	0	17	1
8302	KLRC4	0	0	1
8394	PIP5K1A	0	0	1
8395	PIP5K1B	0	0	1
8396	PIP4K2B	1	0	1
8398	PLA2G6	1	0	1
8399	PLA2G10	0	0	1
8408	ULK1	1	0	1
8454	CUL1	1	0	1
8482	SEMA7A	0	0	1
8569	MKNK1	0	17	1
8600	TNFSF11	0	0	1
8625	RFXANK	0	0	1
8633	UNC5C	1	0	1
8646	CHRD	1	0	1
8650	NUMB	1	0	1
8651	SOCS1	1	10	1
8681	JMJD7-PLA2G4B	0	0	0
8740	TNFSF14	0	0	1
8743	TNFSF10	0	0	1
8764	TNFRSF14	0	12	1
8771	TNFRSF6B	1	0	1
8792	TNFRSF11A	0	18	1
8817	FGF18	1	0	1
8822	FGF17	0	0	1
8823	FGF16	1	0	1
8835	SOCS2	0	12	1
8837	CFLAR	1	0	1
8850	KAT2B	1	0	1
8863	PER3	0	0	1
8864	PER2	1	0	1
8874	ARHGEF7	1	0	1
8877	SPHK1	1	0	1
8915	BCL10	1	10	1
8945	BTRC	1	0	1
9002	F2RL3	0	0	1

9021	SOCS3	1	11	1
9047	SH2D2A	0	0	1
9133	CCNB2	1	0	1
9180	OSMR	0	0	1
9181	ARHGEF2	1	0	1
9241	NOG	1	0	1
9252	RPS6KA5	1	0	1
9253	NUMBL	1	0	1
9350	CER1	0	0	1
9353	SLIT2	1	0	1
9423	NTN1	1	0	1
9451	EIF2AK3	1	0	1
9470	EIF4E2	1	0	1
9541	CIR1	1	0	1
9542	NRG2	1	0	1
9575	CLOCK	1	0	1
9612	NCOR2	1	23	1
9655	SOCS5	0	0	1
9672	SDC3	0	0	1
9693	RAPGEF2	1	0	1
9706	ULK2	0	0	1
9794	MAML1	1	0	1
9863	MAGI2	1	0	1
9899	SV2B	1	0	1
9900	SV2A	1	0	1
9965	FGF19	1	11	1
9978	RBX1	1	0	1
10093	ARPC4	1	0	0
10094	ARPC3	1	0	1
10095	ARPC1B	0	0	1
10109	ARPC2	1	0	1
10125	RASGRP1	0	0	1
10154	PLXNC1	1	0	1
10197	PSME3	1	0	1
10235	RASGRP2	1	0	1
10254	STAM2	1	0	1
10297	APC2	1	0	1
10319	LAMC3	1	0	1
10411	RAPGEF3	0	0	1
10452	TOMM40	1	0	1
10468	FST	1	0	1
10505	SEMA4F	0	0	1
10507	SEMA4D	1	0	1
10509	SEMA4B	0	0	1
10552	ARPC1A	0	0	1
10603	SH2B2	1	0	1
10637	LEFTY1	1	0	1
10666	CD226	0	0	1
10683	DLL3	1	0	1
10718	NRG3	0	0	1
10725	NFAT5	1	0	1
10788	IQGAP2	0	0	1
10800	CYSLTR1	0	0	1
10870	HCST	0	0	1
10892	MALT1	1	15	1

10912	GADD45G	0	0	1
11009	IL24	0	0	1
11069	RAPGEF4	1	0	1
11072	DUSP14	1	0	1
11186	RASSF1	1	0	1
11197	WIF1	1	20	1
11213	IRAK3	0	0	1
11221	DUSP10	1	0	1
11317	RBPJL	1	0	1
22798	LAMB4	0	0	1
22885	ABLIM3	0	0	1
22926	ATF6	0	0	1
22987	SV2C	1	0	1
23220	DTX4	1	0	1
23236	PLCB1	1	0	1
23291	FBXW11	1	0	1
23308	ICOSLG	0	13	1
23385	NCSTN	1	16	1
23433	RHOQ	0	0	1
23529	CLCF1	1	0	1
25780	RASGRP3	0	0	1
25945	NECTIN3	1	0	1
25989	ULK3	0	0	1
26230	TIAM2	0	0	1
26279	PLA2G2D	0	0	1
26281	FGF20	0	0	1
26291	FGF21	0	0	1
27006	FGF22	0	0	0
27035	NOX1	1	0	1
27330	RPS6KA6	0	0	1
28514	DLL1	1	0	1
29949	IL19	0	0	1
29984	RHOD	0	0	1
30814	PLA2G2E	0	0	1
30837	SOCS7	1	0	1
50487	PLA2G3	1	0	1
50508	NOX3	0	0	1
50509	COL5A3	0	0	1
50604	IL20	0	0	1
50615	IL21R	0	21	1
50616	IL22	0	0	1
50831	TAS2R3	0	0	0
50832	TAS2R4	0	0	0
50833	TAS2R16	0	0	1
50834	TAS2R1	0	0	1
50835	TAS2R9	0	0	1
50836	TAS2R8	0	0	0
50837	TAS2R7	0	0	0
50838	TAS2R13	1	0	1
50839	TAS2R10	0	0	1
50840	TAS2R14	0	0	1
50848	F11R	1	0	1
50855	PARD6A	0	0	1
51107	APH1A	1	14	1
51176	LEF1	1	15	1

51206	GP6	0	0	1
51422	PRKAG2	1	0	1
51561	IL23A	0	0	1
51701	NLK	1	0	1
51764	GNG13	0	0	0
53632	PRKAG3	0	0	1
53832	IL20RA	0	0	1
53833	IL20RB	0	0	1
53944	CSNK1G1	1	0	1
54106	TLR9	1	0	1
54205	CYCS	1	0	1
54429	TAS2R5	0	0	0
54472	TOLLIP	0	0	1
54567	DLL4	1	0	1
54910	SEMA4C	1	0	1
55534	MAML3	0	0	1
55740	ENAH	1	0	1
55844	PPP2R2D	0	0	1
55851	PSENEN	1	0	1
56034	PDGFC	1	0	1
56413	LTB4R2	0	0	1
56832	IFNK	0	0	1
56848	SPHK2	1	0	1
56998	CTNNBIP1	1	0	1
57105	CYSLTR2	0	19	1
57154	SMURF1	1	0	1
57369	GJD2	1	0	1
57680	CHD8	1	0	1
57715	SEMA4G	0	0	0
58494	JAM2	0	0	1
58985	IL22RA1	0	0	1
59067	IL21	0	0	1
59343	SENP2	1	0	1
63923	TNN	0	0	1
64109	CRLF2	0	9	1
64218	SEMA4A	0	0	1
64221	ROBO3	1	0	1
64321	SOX17	1	15	1
64600	PLA2G2F	0	0	0
64750	SMURF2	1	0	1
64840	PORCN	1	0	1
79139	DERL1	1	0	1
79660	PPP1R3B	0	0	1
79837	PIP4K2C	0	0	1
80301	PLEKHO2	0	0	0
80310	PDGFD	0	0	1
80319	CXXC4	0	0	1
80824	DUSP16	0	0	1
80834	TAS1R2	0	0	1
80835	TAS1R1	0	0	1
81579	PLA2G12A	1	0	1
81607	NECTIN4	0	0	1
83439	TCF7L1	1	0	1
83700	JAM3	1	0	1
83756	TAS1R3	1	0	1

84134	TOMM40L	0	0	1
84433	CARD11	1	22	1
84441	MAML2	1	18	1
84448	ABLIM2	0	0	1
84552	PARD6G	0	0	0
84612	PARD6B	1	0	0
84647	PLA2G12B	0	0	0
84867	PTPN5	0	0	1
85407	NKD1	1	0	1
85409	NKD2	0	0	1
85417	CCNB3	0	19	1
85477	SCIN	0	0	1
85480	TSLP	0	0	1
90249	UNC5A	1	0	1
113878	DTX2	0	0	1
115653	KIR3DL3	0	0	1
115727	RASGRP4	0	0	1
116379	IL22RA2	0	0	1
117157	SH2D1B	0	0	1
122011	CSNK1A1L	0	0	1
122809	SOCS4	0	0	1
128239	IQGAP3	1	0	1
131873	COL6A6	0	0	0
137970	UNC5D	0	0	1
145957	NRG4	0	0	1
149233	IL23R	0	0	1
150084	IGSF5	0	0	1
151636	DTX3L	0	0	1
163702	IFNLR1	0	0	1
196403	DTX3	1	0	1
196883	ADCY4	1	0	0
200576	PIKFYVE	1	0	1
219699	UNC5B	1	0	1
253314	EIF4E1B	0	0	0
259285	TAS2R39	0	0	0
259286	TAS2R40	0	0	0
259287	TAS2R41	0	0	0
259289	TAS2R43	0	0	1
259290	TAS2R31	0	0	0
259291	TAS2R45	0	0	0
259292	TAS2R46	0	0	1
259294	TAS2R19	0	0	0
259295	TAS2R20	0	0	0
259296	TAS2R50	0	0	1
260425	MAGI3	0	0	1
282616	IFNL2	0	0	1
282617	IFNL3	0	0	1
282618	IFNL1	0	0	1
284217	LAMA1	1	0	1
338376	IFNE	0	0	1
338398	TAS2R60	0	0	1
345456	PFN3	0	0	0
353164	TAS2R42	0	0	1
375189	PFN4	0	0	0
375790	AGRN	1	0	1

646048	-	1	0	0
646821	-	1	0	0
648921	-	1	0	0
649853	-	1	0	0
650832	-	1	0	0
652346	-	1	0	0
652671	-	1	0	0
653361	NCF1	1	0	1
653888	-	1	0	0
728622	SKP1P2	0	0	0
731751	-	1	0	0
1E+08	-	1	0	0
1E+08	PLA2G4B	0	0	1

References

1. Kauffman, S.; Peterson, C.; Samuelsson, B.; Troein, C. Genetic networks with canalyzing Boolean rules are always stable. *Proceedings of the National Academy of Sciences of the United States of America* **2004**, *101*, 17102-17107, doi:10.1073/pnas.0407783101.
2. Kauffman, S.; Peterson, C.; Samuelsson, B.; Troein, C. Random Boolean network models and the yeast transcriptional network. *Proceedings of the National Academy of Sciences* **2003**, *100*, 14796-14799, doi:10.1073/pnas.2036429100.
3. Samal, A.; Jain, S. The regulatory network of E. coli metabolism as a Boolean dynamical system exhibits both homeostasis and flexibility of response. *BMC Systems Biology* **2008**, *2*, 21, doi:10.1186/1752-0509-2-21.
4. Naldi, A.; Carneiro, J.; Chaouiya, C.; Thieffry, D. Diversity and Plasticity of Th Cell Types Predicted from Regulatory Network Modelling. *Plos Computational Biology* **2010**, *6*, e1000912, doi:10.1371/journal.pcbi.1000912.
5. Harris, S.E.; Sawhill, B.K.; Wuensche, A.; Kauffman, S. A model of transcriptional regulatory networks based on biases in the observed regulation rules. *Complexity* **2002**, *7*, 23-40, doi:10.1002/cplx.10022.
6. Trinh, H.C.; Kwon, Y.K. Edge-based sensitivity analysis of signaling networks by using Boolean dynamics. *Bioinformatics* **2016**, *32*, i763-i771. doi:10.1093/bioinformatics/btw464.
7. Trinh, H.-C.; Kwon, Y.-K. Effective Boolean dynamics analysis to identify functionally important genes in large-scale signaling networks. *Biosystems* **2015**, *137*, 64-72, doi:https://doi.org/10.1016/j.biosystems.2015.07.007.
8. Seoane, J.A.; Campbell, C.; Day, I.N.M.; Casas, J.P.; Gaunt, T.R. Canonical Correlation Analysis for Gene-Based Pleiotropy Discovery. *PLOS Computational Biology* **2014**, *10*, e1003876, doi:10.1371/journal.pcbi.1003876.
9. Mazaya, M.; Trinh, H.-C.; Kwon, Y.-K. Construction and analysis of gene-gene dynamics influence networks based on a Boolean model. *BMC Systems Biology* **2017**, *11*, 133, doi:10.1186/s12918-017-0509-y.
